# Deployment-oriented evaluation of stage-specific information for rice yield prediction across years, sites and site-year environments in southern China

**DOI:** 10.3389/fpls.2026.1915306

**Published:** 2026-09-08

**Authors:** Jinshuai Zhang, Yongquan Xiong, Shuyao Zhou, Qing Ouyang, Ming Huang, Tao Guo, Chun Chen, Yongzhu Liu

**Affiliations:** 1 National Engineering Research Center of Plant Space Breeding, College of Agriculture, South China Agricultural University, Guangzhou, China; 2 School of Accounting, Anyang Institute of Technology, Anyang, China; 3 College of Artificial Intelligence and Low Altitude Technology, South China Agricultural University, Guangzhou, China

**Keywords:** BLUP, cultivar genetic potential, external validation, field survey, machine learning, rice yield prediction, stage-specific climate

## Abstract

**Background:**

Reliable rice yield prediction requires machine-learning models with inputs that match the intended decision point and evaluation schemes that reflect the target deployment setting. Random record-level cross-validation remains common in crop-yield machine learning, but it can overestimate performance when models are transferred to future seasons, new sites or new site-year environments.

**Methods:**

We developed a scenario-aware machine-learning framework using 3,204 yield records from 168 cultivars evaluated across 10 sites, 7 years and 67 site-year environments in southern China. Five modeling feature sets were compared across random five-fold cross-validation, leave-one-year-out validation, forward-year prediction, leave-one-site-out validation and leave-one-site-year-out validation. The feature sets represented whole-season weather, stage-specific climate information, leakage-controlled cultivar yield BLUP, field-survey traits and heading-stage updates.

**Results:**

Random five-fold cross-validation produced the highest apparent accuracy, with the best field-survey-enhanced model reaching R^2^ = 0.844 and RMSE = 340.59 kg ha^−1^. Deployment-oriented performance was lower. The same information scenario achieved a macro mean R^2^ of 0.359 in forward-year prediction, 0.290 in leave-one-site-out validation and 0.131 in leave-one-site-year-out validation. Stage-specific climate information and leakage-controlled cultivar potential improved prediction before field-survey traits were available, whereas field-survey traits provided the strongest later-stage accuracy. Reduced training size lowered accuracy and increased unseen-cultivar burden. Climate-risk environments showed risk-specific error shifts rather than a uniform model failure.

**Conclusions:**

This study supports a deployment-oriented framework for rice yield prediction, rather than a general claim of universal out-of-sample improvement. Its main value is to show how prediction skill, feature utility and model interpretation change when validation is aligned with realistic temporal, spatial and site-year deployment settings.

## 1 Introduction

Accurate crop yield prediction is important for variety evaluation, production planning and agricultural risk management. Rice (*Oryza sativa L.*) is one of the world’s most important staple crops, and its yield is determined by interactions among cultivar characteristics, environmental conditions and crop-development dynamics that vary across locations and seasons (Liakos et al., 2018; Kamilaris and Prenafeta-Boldu, 2018). Machine learning, deep learning and explainable artificial intelligence have expanded the toolkit for crop-yield modeling, including rice-yield prediction reviews, multimodal neural-network approaches for genotype, environment and management interactions, interpretable remote-sensing yield models and AI-enabled envirotyping or multi-environment prediction frameworks (van Klompenburg et al., 2020; Ajay et al., 2025; De Clercq and Mahdi, 2025; Petropoulos et al., 2025; Sajid et al., 2025; Liang et al., 2026; Yue et al., 2026). Despite this rapid methodological development, an unresolved issue is whether reported model performance remains credible when the validation split matches the deployment task faced by breeding programs.

A recurring limitation in crop-yield machine learning is the mismatch between model evaluation and real-world deployment. Randomly splitting field-trial records into training and test sets can place the same cultivars, sites or closely related environments in both partitions. This design is suitable for internal model comparison, but it can overestimate predictive performance when the operational objective is to forecast a future season or generalize to a new location. Structured and blocked cross-validation strategies are therefore recommended when datasets contain spatial or temporal dependence and the modeling goal is extrapolation (Roberts et al., 2017; Meyer et al., 2019; Sweet et al., 2023). Morales and Villalobos (2023) demonstrated this random-versus-chronological partitioning effect using simulated crop data. The present study extends that question to real multi-cultivar rice trial data and adds spatial and site-year extrapolation tests.

The timing of information availability creates an additional challenge. Whole-season weather summaries are useful for retrospective analysis but are unavailable during early-season forecasting, when predictions may have greater management value. Field-survey traits can improve accuracy near harvest, but they require in-season observations and therefore represent a later decision point. Previous rice-yield studies have shown that integrating phenological and climate information can improve prediction accuracy (Guo et al., 2021), and large-scale multi-source approaches have explored timely prediction before maturity (Cao et al., 2021). However, these application scenarios answer different practical questions and should not be pooled into a single model evaluation. Combining inputs from multiple crop-development stages without distinguishing their availability windows may misrepresent when reliable predictions can be obtained.

Historical cultivar performance provides another source of predictive information. Multi-environment trials accumulate evidence about cultivar performance across heterogeneous conditions, and this information can support yield prediction. Best linear unbiased prediction (BLUP) provides an environment-adjusted summary of cultivar performance and has long been used in regional yield-trial analysis (Piepho, 1994). Reaction-norm and multi-environment modeling studies have further shown the value of explicitly representing environmental variation and genotype-by-environment interactions for prediction (Jarquín et al., 2014; Crossa et al., 2017; Westhues et al., 2022). Recent crop-prediction studies now combine BLUP, machine learning, deep learning, envirotyping and phenomic or meteorological features, highlighting both the promise of richer information streams and the need to avoid information leakage when cultivar history is used as a predictor (Kick and Washburn, 2023; Sajid et al., 2025; Liang et al., 2026; Yue et al., 2026).

Here, we developed a deployment-oriented machine-learning framework for rice yield prediction using multi-year, multi-site field-trial data from South China. We separated baseline, weather-only, cultivar-history, heading-stage and field-survey-enhanced prediction scenarios. We then evaluated these scenarios under validation schemes that represented internal accuracy, temporal transfer, spatial transfer and unseen site-year environments. The analysis was designed to identify which information sources are useful at different decision points and where model generalization remains limited.

## 2 Materials and methods

### 2.1 Study data and quality control

The dataset was derived from two sources. Yield, phenological and agronomic-trait records were obtained from the official national variety registration trial reports for the Southern Rice Region of China, administered by the National Agricultural Technology Extension and Service Center under the Ministry of Agriculture and Rural Affairs. Daily meteorological data were obtained from the China Meteorological Data Service Centre.

Field trials were conducted at 10 test sites in southern China: Gaozhou, Guangzhou, Huizhou, Qingyuan and Zhaoqing in Guangdong Province; Nanning, Qinzhou and Yulin in Guangxi Zhuang Autonomous Region; Haikou in Hainan Province; and Zhangzhou in Fujian Province. The dataset covered seven growing seasons from 2016 to 2022. Trials included early-season rice, sown in February or March, and late-season rice, sown in July. Because the two sowing seasons experience distinct thermal and radiation conditions, sowing season was retained as an explicit model feature. A site-year combination was defined as an environment throughout this study. The final dataset contained 67 site-year environments.

The raw dataset included 3,205 records from 205 raw cultivar labels. Cultivar names were standardized by resolving spacing differences and trailing control markers, yielding 168 standardized cultivars. Yield values were converted from kg mu^−1^ to kg ha^−1^ using a factor of 15. Phenological dates were parsed to derive year, site-year environment, sowing day of year and sowing month. Days to heading, grain-filling duration and days to maturity were recalculated from reported dates, while the original recorded values were retained for audit purposes. Four rows required numeric cleaning, eight rows contained inconsistencies between recorded and date-derived phenological durations, and five biologically implausible trait values were set to missing. One record had missing yield and was excluded from model evaluation, resulting in 3,204 modeling records. No climate values were missing after quality control.

### 2.2 Climate, phenology and field-survey features

The input data included 27 stage-specific climate variables. Nine metrics were summarized for three crop-development intervals: sowing to heading, heading to maturity and sowing to maturity. The metrics were mean temperature, maximum temperature, minimum temperature, active growing degree days, effective growing degree days, total precipitation, mean relative humidity, total solar radiation and sunshine duration. No climate values were missing after quality control.

Sowing season was classified as early season for February or March sowing and late season for July sowing. Heading day of year was derived from the observed heading date and used only in the heading-stage update scenario. Field-survey traits included growth duration, days to heading, grain-filling duration, effective panicles, plant height, panicle length, total grains per panicle, filled grains per panicle, seed-setting rate and thousand-grain weight.

### 2.3 Leakage-controlled cultivar genetic potential

Historical cultivar yield potential was represented using a training-partition-specific BLUP feature. Within each training partition, we fitted a linear mixed model:


$$Y_{ij}=\mu +E_{j}+G_{i}+\epsilon_{ij}$$

where Y_ij_ is the observed yield of cultivar i in environment j, μ is the intercept, E_j_ is the fixed effect of the site-year environment, G_i_ is the random cultivar intercept and ϵ_ij_ is the residual. The derived cultivar feature was the training-partition mean plus the estimated cultivar random effect.

To prevent information leakage, the BLUP feature for each outer test partition was estimated exclusively from the corresponding outer training partition. Training-row BLUP values were generated by five-fold inner cross-fitting, so each downstream training row received a BLUP estimate from a model that excluded its inner validation fold. If a cultivar in the outer test partition had not appeared in the training partition, it received the training-partition intercept and a zero cultivar effect. These fallback events were recorded for audit. The BLUP feature definitions are summarized in **Supplementary Table 1**.

### 2.4 Prediction scenarios and feature sets

**Figure 1:**
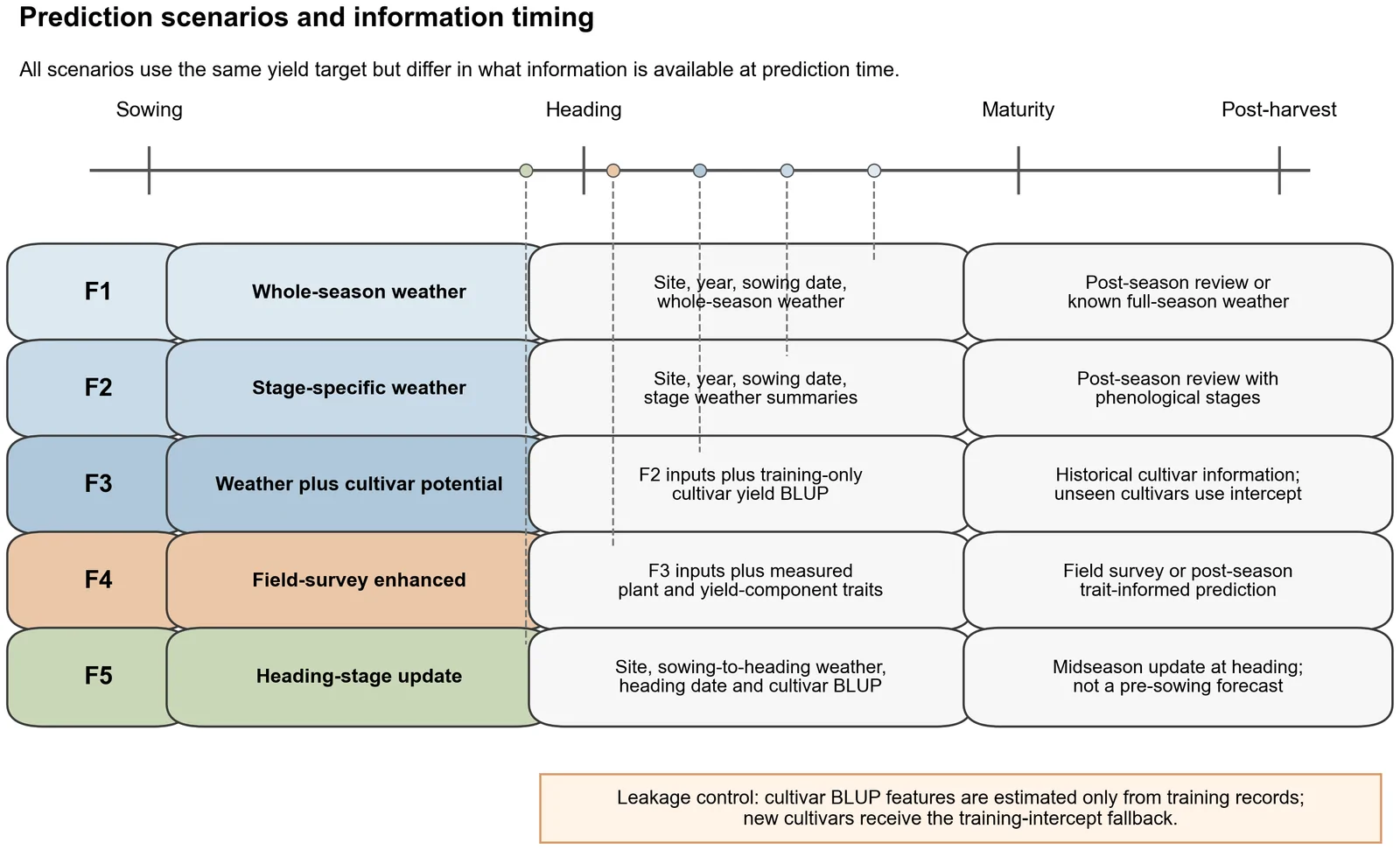
Prediction scenarios and information timing for F1–F5.

We constructed six feature sets to represent distinct application scenarios (**Table 1**; **Figure 1**). The schematic shows when each input type becomes available and clarifies that F4 is a field-survey or post-season estimation scenario rather than an early-season forecast.

**Table 1:** Feature sets and application scenarios.

Feature set	Description	Application scenario	Required features	Optional features	Application boundary
F0	Simple baselines	Benchmark	0	0	No machine-learning predictor matrix. | All baseline statistics must be estimated from the training partition only.
F1	Whole-season weather baseline	Known whole-season weather or postseason review	14	0	Uses weather aggregated from sowing to maturity. | Not a true pre-sowing forecast without phenology prediction and weather forecast inputs. | Year encoding requires cautious interpretation in forward-year validation.
F2	Stage-specific weather	Known stage weather or postseason review	23	0	Uses observed stage boundaries and weather through maturity. | Not a true pre-sowing forecast without phenology prediction and weather forecast inputs. | Year encoding requires cautious interpretation in forward-year validation.
F3	Stage-specific weather plus genetic potential	Historical cultivar information scenario	24	5	BLUP features must be estimated from the training partition only. | Training rows require inner out-of-fold BLUP construction. | Unseen test varieties receive the training-fold intercept fallback.
F4	Field-survey-enhanced model	Field-survey or postseason enhanced prediction	34	5	Contains measured agronomic traits and must be reported separately from F1-F3. | BLUP leakage controls remain mandatory.
F5	Heading-stage update model	Midseason update at heading	15	5	Uses actual Heading DOY and observed sowing-to-heading weather. | Represents an update at heading, not a pre-sowing forecast. | BLUP leakage controls remain mandatory.

- F0 contained training-partition-only baselines, including the overall mean, environment mean with fallback and an environment-random-effect mixed model.
- F1 contained test site, year, sowing information and nine whole-season climate variables.
- F2 replaced whole-season climate variables with 18 stage-specific climate variables for the sowing-to-heading and heading-to-maturity periods.
- F3 added the leakage-controlled cultivar yield BLUP to F2.
- F4 added 10 observed agronomic traits to F3 and represented a field-survey-enhanced prediction scenario.
- F5 represented a heading-stage update scenario using test site, sowing information, sowing-to-heading climate, cultivar yield BLUP and heading day of year.

F4 and F5 were interpreted separately because their inputs become available at different crop development stages. F4 should not be interpreted as a pre-sowing or early-season forecast.

To make these distinctions clear, we added a prediction-scenario schematic that maps F1-F5 to the timing of information availability from sowing through heading, maturity and post-harvest assessment (**Figure 1**).

### 2.5 Machine-learning models and preprocessing

We compared linear regression, ridge regression (Hoerl and Kennard, 1970), ElasticNet (Zou and Hastie, 2005), random forest (Breiman, 2001), XGBoost (Chen and Guestrin, 2016), LightGBM (Ke et al., 2017) and CatBoost (Prokhorenkova et al., 2018). These algorithms cover regularized linear models, bagging and gradient boosting, which are widely used for tabular crop-yield prediction. Numeric variables were imputed using the training-partition median and standardized. Categorical variables were imputed using the most frequent training-partition value and one-hot encoded, with unknown categories ignored. All preprocessing operations were fitted within the relevant training partition. The outcome variable was continuous yield, so class imbalance was not applicable.

The main model parameters were fixed across validation schemes to support comparable scenario contrasts. Ridge regression used α = 10. ElasticNet used α = 0.01 and an l_1_ ratio of 0.20. Random forest used 300 trees, a minimum leaf size of 2 and square-root feature sampling. XGBoost and LightGBM used 350 boosting iterations and a learning rate of 0.04. LightGBM used 20 leaves and 15 minimum child samples. CatBoost used 350 iterations, a learning rate of 0.04 and tree depth 6. The random seed was fixed at 42.

To test whether fixed hyperparameters affected the comparison between ElasticNet and LightGBM, we added a nested tuning sensitivity check for F4 in forward-year and leave-one-site-out validation. Hyperparameters were selected by inner three-fold cross-validation within each outer training partition and then evaluated on the corresponding held-out partition.

### 2.6 Validation schemes

We used five validation schemes with progressively stricter generalization requirements. This design follows recommendations that validation partitions should reflect the intended extrapolation task when observations contain temporal or spatial structure (Roberts et al., 2017; Meyer et al., 2019).

1. Random five-fold cross-validation, assessed internal discrimination when related records could occur in both training and test folds.
2. Leave-one-year-out validation, held out each of the seven years in turn.
3. Forward-year prediction, trained on all prior years and predicted the next target year, producing six temporal splits.
4. Leave-one-site-out validation, held out each of the 10 test sites in turn.
5. Leave-one-site-year-out validation, held out each of the 67 site-year environments in turn.

For each split, preprocessing and BLUP estimation were repeated after partitioning. Metrics included R^2^, RMSE, MAE, normalized RMSE (nRMSE), Spearman correlation and recall among the top 20% of observed yields. We report both macro means across held-out units and pooled micro metrics where appropriate.

For the revision, we added two additional baselines: a cultivar BLUP-only predictor and a simple multiple linear regression model using a small set of climate and sowing variables. These baselines were evaluated under the same validation schemes as the main models.

### 2.7 Sample-size robustness

To quantify sensitivity to training sample size, we created a fixed random external test set containing 641 records and a training pool containing 2,563 records. The training pool was reduced to 20%, 40%, 60%, 80% and 100% of its original size. Each reduced-training analysis was repeated 20 times. This experiment was used only to assess sample-size sensitivity and should not be interpreted as a deployment-validation scheme.

### 2.8 Extreme-environment analysis

We audited robustness under climate-risk environments using predictions from leave-one-site-year-out validation. Five heading-to-maturity risk metrics were considered: high maximum temperature, heavy rainfall, high relative humidity, low sunshine duration and low solar radiation. For each metric, risk environments were defined using the most extreme 10% of site-year environments.

The extreme-environment analysis focused on F4 with ElasticNet because this configuration achieved the best leave-one-site-year-out performance. We also tested differences between flagged and unflagged environments using Welch’s t-test and the Mann-Whitney U test and repeated the analysis using a stricter definition that required at least two risk flags.

### 2.9 Model interpretation

We fitted descriptive final models for three scenarios: F3 with LightGBM, F4 with LightGBM and F5 with random forest. The cultivar yield BLUP used for interpretation was generated by five-fold out-of-fold cross-fitting before final model fitting. Model interpretation used SHAP values because they provide additive local explanations that can be aggregated to feature and information-group levels (Lundberg and Lee, 2017). This made the interpretation comparable across tree-based models and scenario-specific feature sets.

### 2.10 Sensitivity analysis without the numeric year feature

Because F1-F4 included numeric year as an input, we repeated forward-year prediction after removing this variable. Training years, target years, preprocessing rules, algorithms and BLUP estimates were otherwise held constant. This sensitivity analysis assessed whether temporal performance depended on simple extrapolation of the numeric year trend.

## 3 Results

### 3.1 Multi-environment trials provided heterogeneous conditions for model evaluation

**Figure 2:**
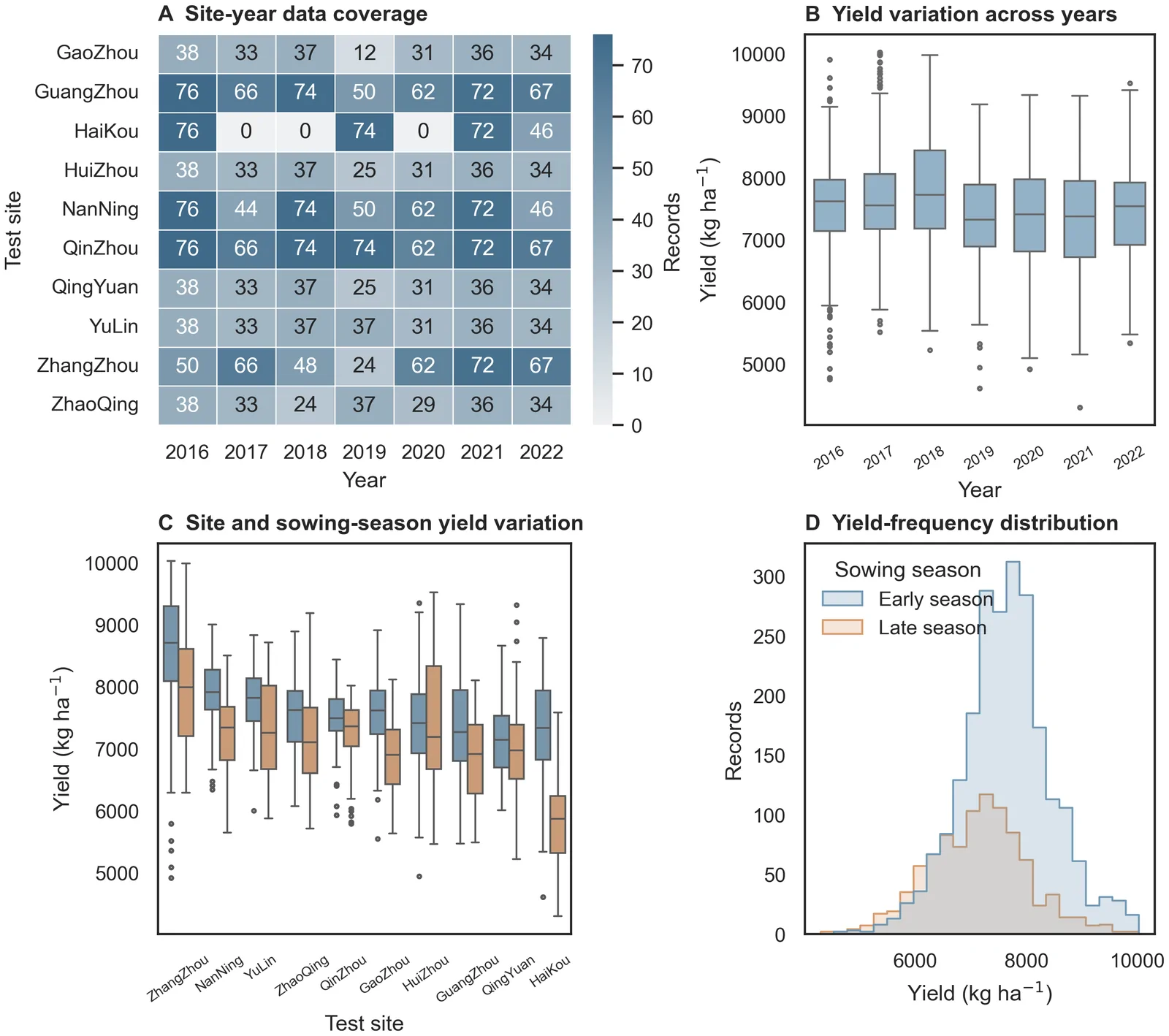
Dataset overview and yield variation. **(A)** Site-year data coverage across test sites and years. **(B)** Annual yield distribution. **(C)** Yield distribution by test site and sowing season. **(D)** Yield-frequency distribution for early- and late-season records.

The final dataset contained 3,204 records with non-missing yield from 168 cultivars across 10 sites, 7 years and 67 site-year environments (**Table 2**). The dataset was imbalanced across sites and years, with 12–76 records per site-year environment. Early-season trials accounted for 2,262 records and late-season trials for 943 records. Yield distributions varied across years, sites and sowing seasons (**Figure 2**). Several climate variables were strongly correlated within and across crop-development stages (**Supplementary Figure 1**), supporting the use of regularized and tree-based models alongside feature-group interpretation.

**Table 2:** Dataset overview.

Dataset characteristic	Value
Raw records	3205
Records with non-missing yield	3204
Standardized cultivars	168
Raw cultivar labels	205
Test sites	10
Years	7
Site-year environments	67
Stage-specific climate variables	27
Early-season records	2262
Late-season records	943

### 3.2 Stage-specific climate and cultivar genetic potential improved internal prediction

**Figure 3:**
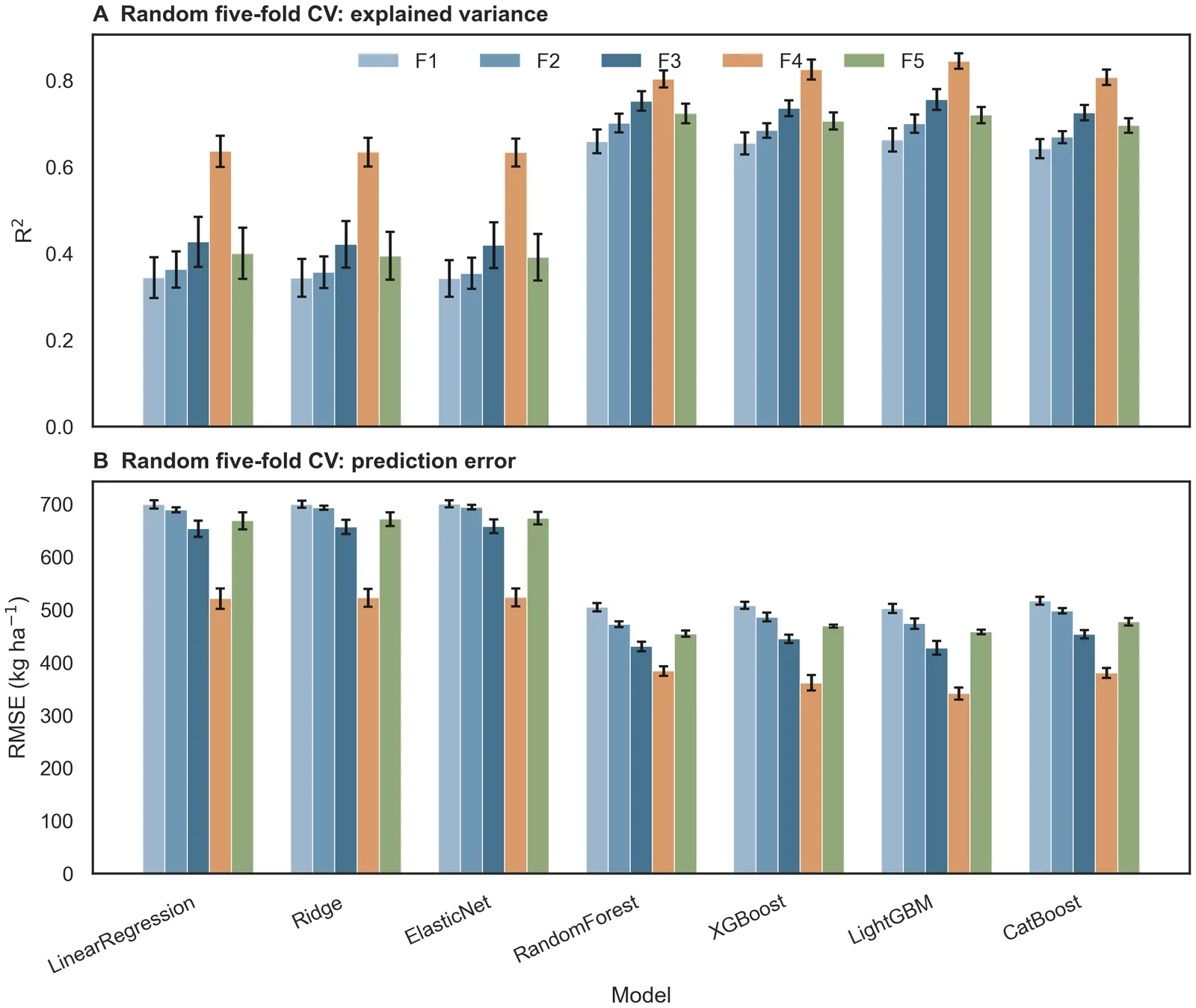
Random five-fold cross-validation performance. **(A)** Mean R^2^ across folds for different feature sets and machine-learning algorithms. **(B)** Mean RMSE across folds for different feature sets and machine-learning algorithms. Error bars indicate variability among folds.

To quantify the incremental value of different information sources, we compared the feature sets under random five-fold cross-validation (**Figure 3**; **Supplementary Table 2**). Among the F0 baselines, the environment-random-effect mixed model performed best, with R^2^ = 0.402 and RMSE = 667.43 kg ha^−1^. The best F1 model, which used whole-season climate information, achieved R^2^ = 0.662 and RMSE = 501.43 kg ha^−1^. Replacing whole-season climate with stage-specific climate variables improved the best F2 R^2^ to 0.701. Adding leakage-controlled cultivar yield BLUP further increased the best F3 R^2^ to 0.755, with RMSE = 426.97 kg ha^−1^.

The field-survey-enhanced F4 scenario produced the strongest internal performance. LightGBM achieved R^2^ = 0.844, RMSE = 340.59 kg ha^−1^ and MAE = 252.52 kg ha^−1^. Because F4 includes traits closely related to yield formation, we added a no-leakage multiplicative yield-component baseline. Under leave-one-site-year-out validation, the training-calibrated index reached micro R^2^ = 0.339 and RMSE = 702.91 kg ha^−1^, whereas F4 ElasticNet reached micro R^2^ = 0.523 and RMSE = 596.99 kg ha^−1^. Thus, F4 should be interpreted as a field-survey-enhanced estimation scenario, but the multivariate model still added value beyond a simple yield-component reconstruction.

### 3.3 Random cross-validation overestimated temporal generalization

**Figure 4:**
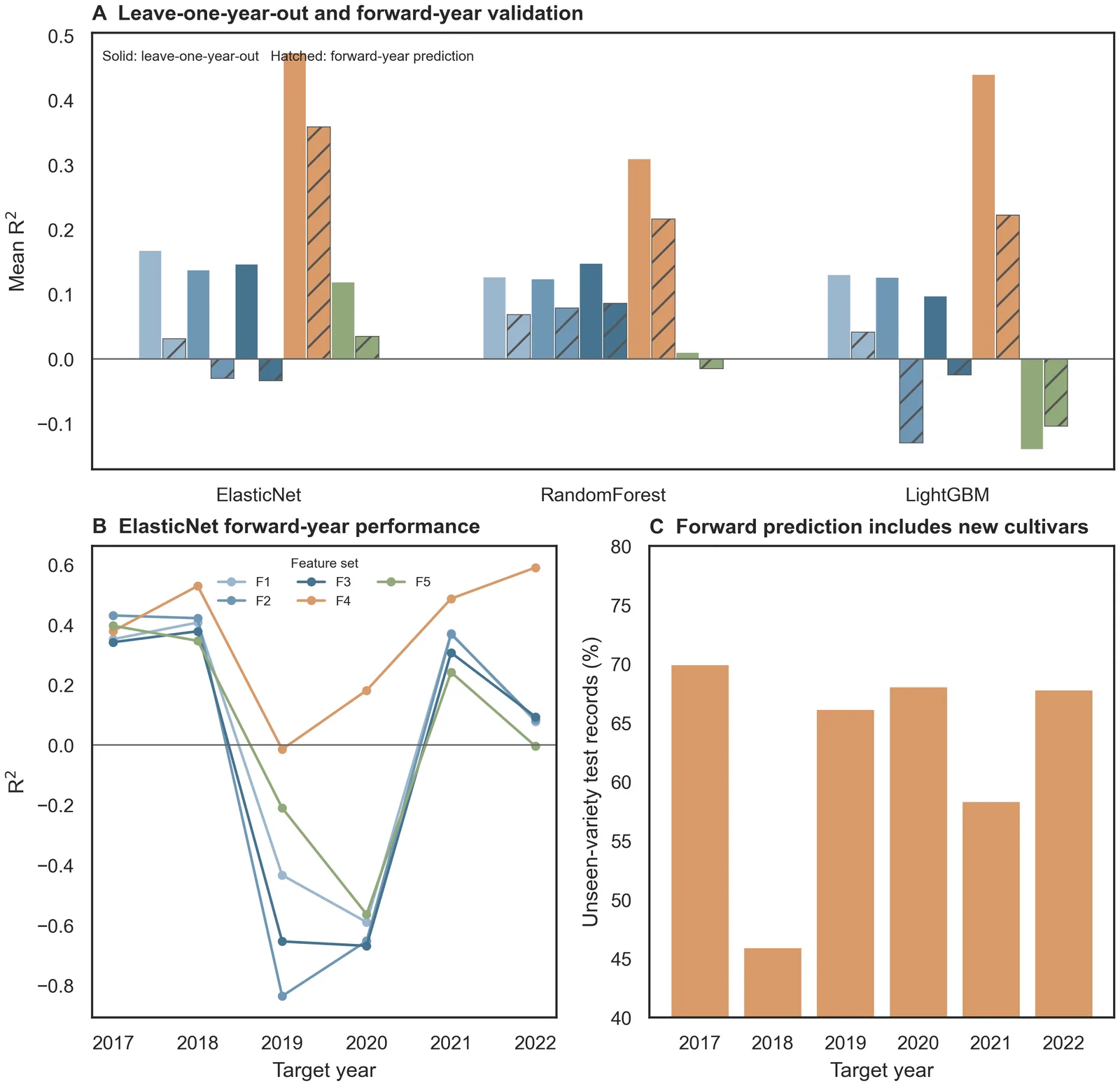
Temporal generalization of rice yield prediction models. **(A)** Mean R^2^ under leave-one-year-out and forwardyear validation across feature sets and algorithms. **(B)** Forward-year R² by target year and feature set. **(C)** Proportion of unseen-cultivar test records by target year.

To examine deployment-oriented performance, we evaluated models across withheld years and forward-year splits (**Figure 4**; **Supplementary Tables 3**, **4**). Under leave-one-year-out validation, F4 with ElasticNet achieved the best mean R^2^ of 0.474 and an RMSE of 605.26 kg ha^−1^. Under the stricter forward-year design, in which only prior years were used for training, F4 with ElasticNet remained the best configuration but its mean R^2^ decreased to 0.359 and RMSE increased to 673.61 kg ha^−1^.

The F0 environment-random-effect baseline also deteriorated under temporal transfer, with mean R^2^ values of -0.044 in leave-one-year-out validation and -0.050 in forward-year prediction. Because target-year site-year environments were absent from the corresponding training partitions, environment effects could not be directly transferred. Forward-year prediction additionally included substantial cultivar turnover. Across six target years, 1,659 test records (62.37%) belonged to cultivars absent from the corresponding training periods and therefore received the training-intercept BLUP fallback. This burden ranged from 45.93% to 69.95% across target years. The reduction from random cross-validation to forward-year prediction indicated that internal cross-validation substantially overstated future-season generalization.

Removing numeric year from F1-F4 did not weaken the best field-survey-enhanced model (**Supplementary Figure 4**; **Supplementary Table 13**). For F4 with ElasticNet, mean forward-year R^2^ changed from 0.359 to 0.365. The corresponding values were 0.068 to 0.055 for F1 with random forest, 0.106 to 0.035 for F2 with CatBoost and 0.086 to 0.071 for F3 with random forest. Thus, the F4 advantage was not attributable to simple extrapolation of the numeric year feature.

### 3.4 Spatial and site-year extrapolation exposed clear deployment boundaries

**Figure 5:**
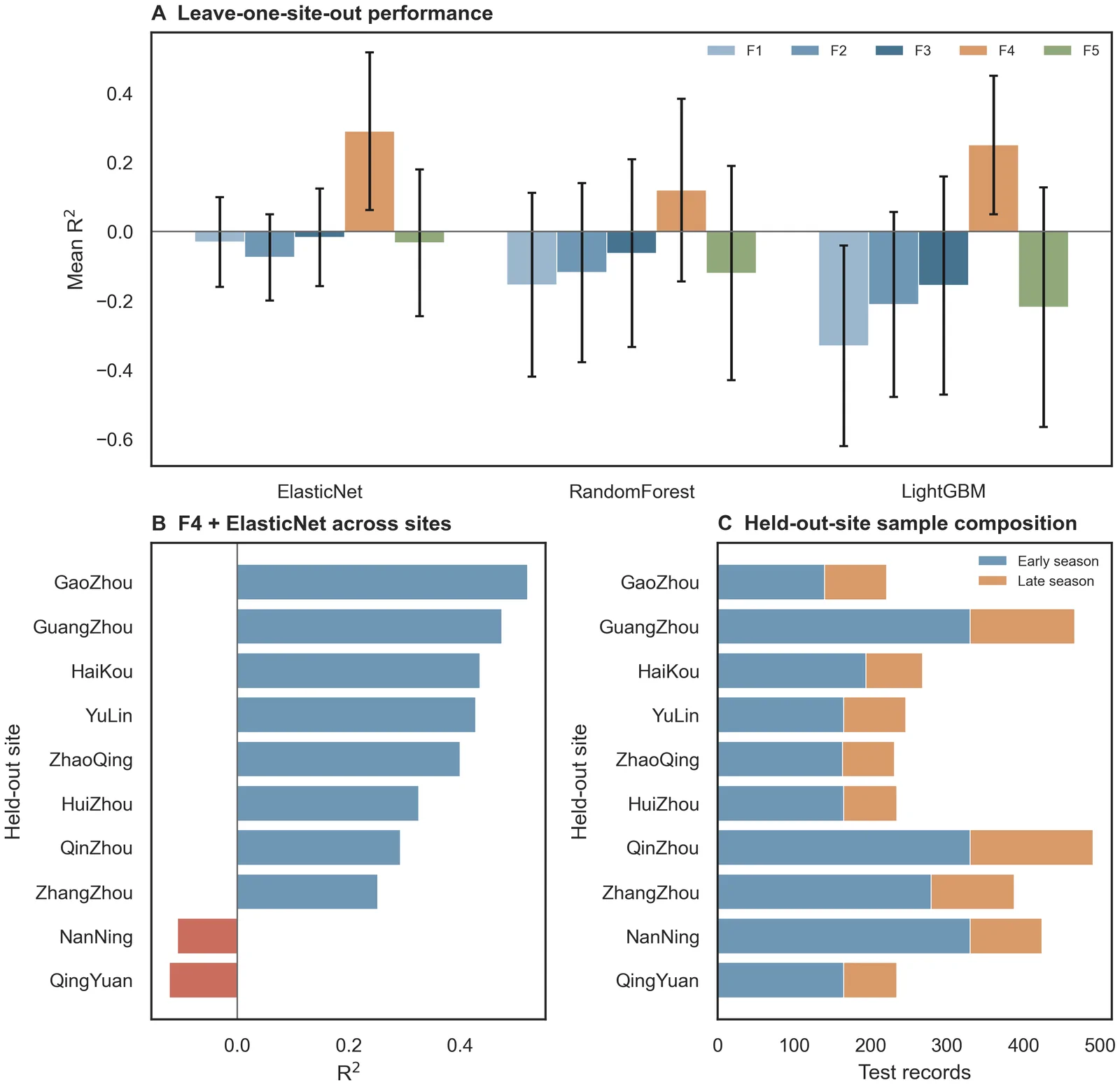
Generalization to unseen sites. **(A)** Mean R^2^ across feature sets and algorithms under leave-one-site-out validation. **(B)** Site-specific R^2^ values for the best-performing configuration. **(C)** Held-out-site sample composition.

We next held out test sites to assess spatial transfer (**Figure 5**; **Supplementary Tables 5**, **6**). F4 with ElasticNet again provided the best mean performance, with an R^2^ of 0.290 and an RMSE of 618.03 kg ha^−1^. The configuration achieved positive R^2^ values in 8 of the 10 held-out sites. In contrast, the best F1-F3 and F5 models had mean R^2^ values close to or below zero, showing that field-survey traits were particularly important when models were transferred to new locations.

**Figure 6:**
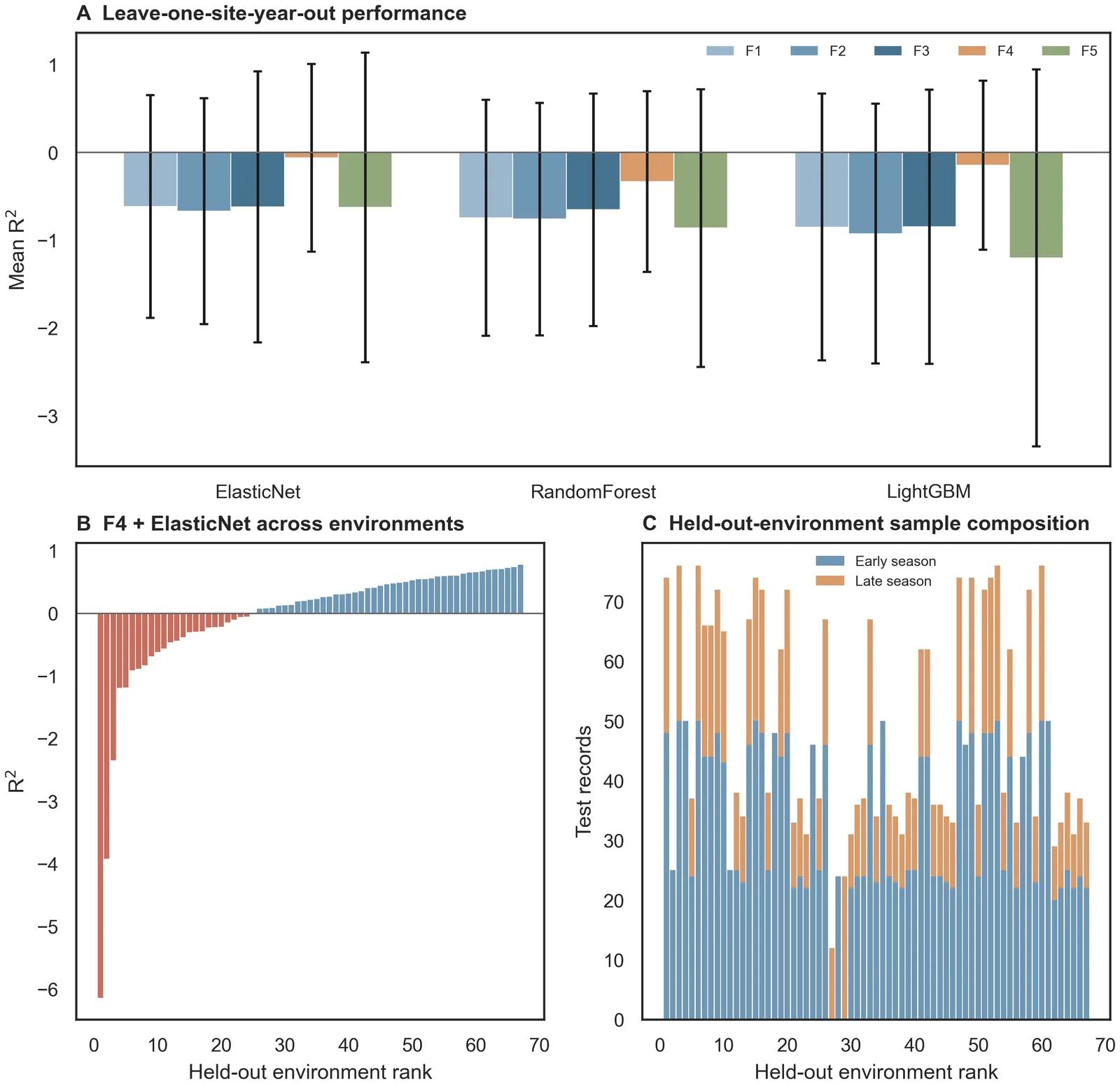
Generalization to unseen site-year combinations. **(A)** Mean R^2^ across feature sets and algorithms under leave-one-site-year-out validation. **(B)** Environmentspecific R^2^ values for the best-performing configuration. **(C)** Held-out-environment sample composition.

The leave-one-site-year-out analysis represented a harder setting in which each specific site-year combination was withheld in turn (**Figure 6**; **Supplementary Tables 7**, **8**, **14**, **16**). F4 with ElasticNet had a macro mean R^2^ of -0.063, but this average was affected by small or low-variance held-out environments. The same predictions gave a micro-averaged R^2^ of 0.523, RMSE of 596.99 kg ha^−1^ and nRMSE of 0.080. Environment-level R^2^ was positively associated with within-environment yield standard deviation (Spearman ρ = 0.41), supporting the interpretation that the model estimated site-year yield level better than it ranked cultivars within every environment.

The best-performing configurations under all five validation schemes are summarized in **Table 3**.

**Table 3:** Comparison of validation schemes.

Validation scheme	Feature set	Model	R^2^	RMSE	MAE	Spearman correlation	Top-20% recall
Random five-fold CV	F4	LightGBM	0.844	340.593	252.518	0.9	0.803
Leave-one-year-out	F4	ElasticNet	0.474	605.263	468.191	0.668	0.587
Forward-year prediction	F4	ElasticNet	0.359	673.605	525.059	0.61	0.578
Leave-one-site-out	F4	ElasticNet	0.29	618.026	493.874	0.648	0.506
Leave-one-site-year-out	F4	ElasticNet	-0.063	550.016	454.502	0.628	0.502

Additional paired tests across outer folds or held-out units are provided in **Supplementary Table 15**. The F3 to F4 improvement was statistically supported in forward-year validation, while smaller differences among leading algorithms were less stable across splits.

### 3.5 Training sample size affected both accuracy and cultivar coverage

**Figure 7:**
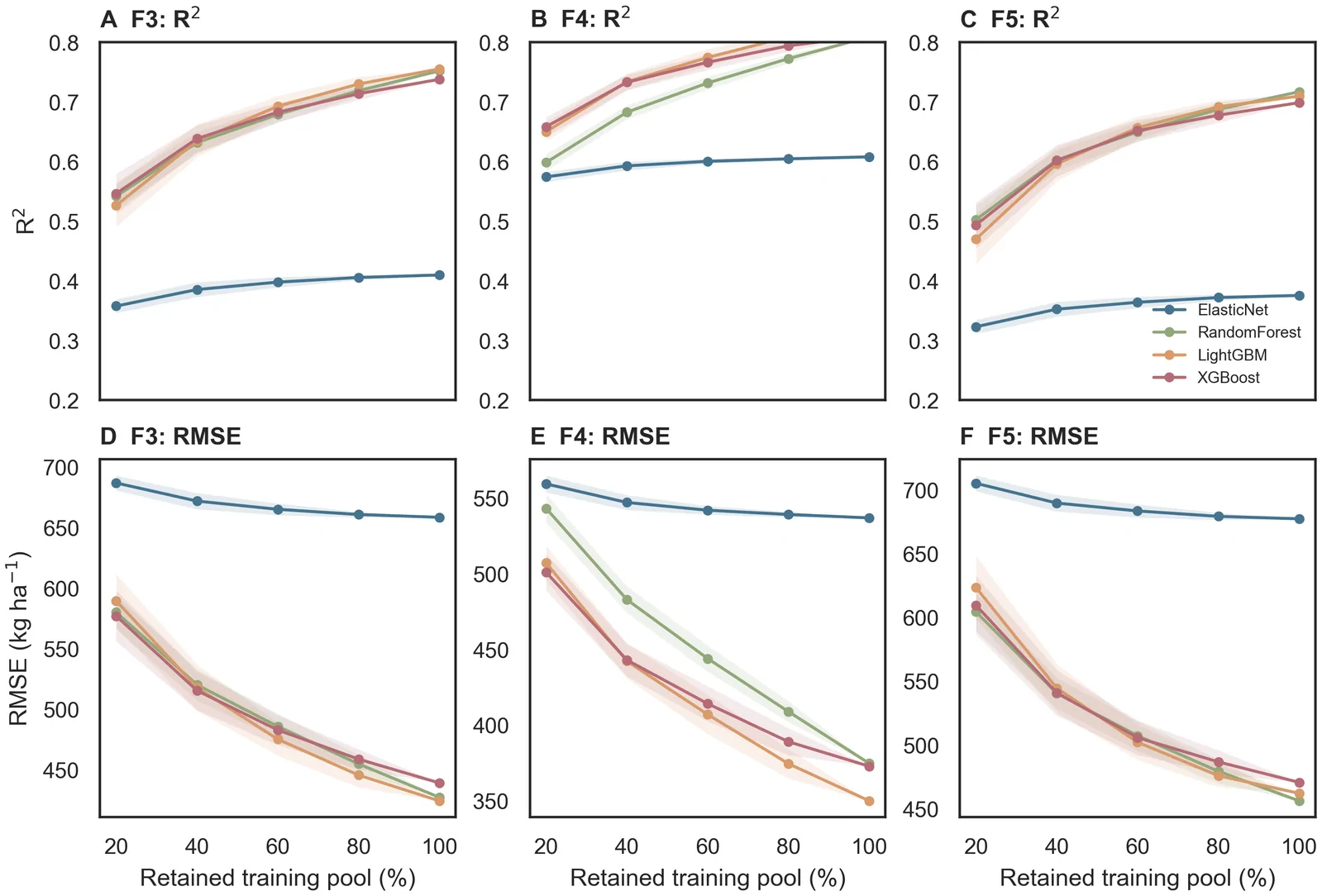
Robustness to reduced training sample size. **(A–C)** R^2^ across retained training fractions for F3, F4 and F5 scenarios. **(D–F)** RMSE across retained training fractions for F3, F4 and F5 scenarios.

Sample-reduction experiments showed that model performance improved consistently as the retained training fraction increased (**Figure 7**; **Table 4**; **Supplementary Table 9**). At 20% of the training pool, the best F3, F4 and F5 models achieved mean R^2^ values of 0.546, 0.658 and 0.502, respectively. With the full training pool, the corresponding values increased to 0.755, 0.833 and 0.717. Because this analysis used a random fixed external test set, we use it only to describe sample-size sensitivity, while deployment conclusions rely on the temporal, spatial and site-year validation schemes.

**Table 4:** Full-training anchors for sample-reduction experiments.

Feature set	Model	R^2^	RMSE	MAE	Top-20% recall
F3	ElasticNet	0.41	658.511	508.08	0.527
F3	LightGBM	0.755	424.083	317.746	0.682
F3	Random forest	0.752	426.958	312.511	0.729
F3	XGBoost	0.738	438.909	338.631	0.651
F4	ElasticNet	0.608	536.796	415.526	0.605
F4	LightGBM	0.833	349.914	259.919	0.806
F4	Random forest	0.809	374.626	278.871	0.76
F4	XGBoost	0.811	372.752	284.961	0.783
F5	ElasticNet	0.375	677.343	520.969	0.504
F5	LightGBM	0.709	461.936	338.955	0.674
F5	Random forest	0.717	456.018	328.924	0.705
F5	XGBoost	0.699	470.536	354.387	0.643

Reduced sample size also affected cultivar coverage. At 20% of the training pool, the external test set contained an average of 37.8 records from cultivars absent from the reduced training data, with a maximum of 63 records across repeated subsamples. This decreased to 2.7 records at 40%, and no outer-test BLUP fallback was required at 60% or higher. These results linked sample-size sensitivity to both statistical information and historical cultivar coverage.

Forward-year and leave-one-site-out predictions were also stratified by whether test cultivars had appeared in the corresponding training set (**Supplementary Table 17**). In forward-year validation, 1,659 of 2,660 test records were from cultivars unseen during training, confirming that cultivar turnover was a major deployment constraint.

### 3.6 Climate-risk environments showed limited and risk-specific error shifts

**Figure 8:**
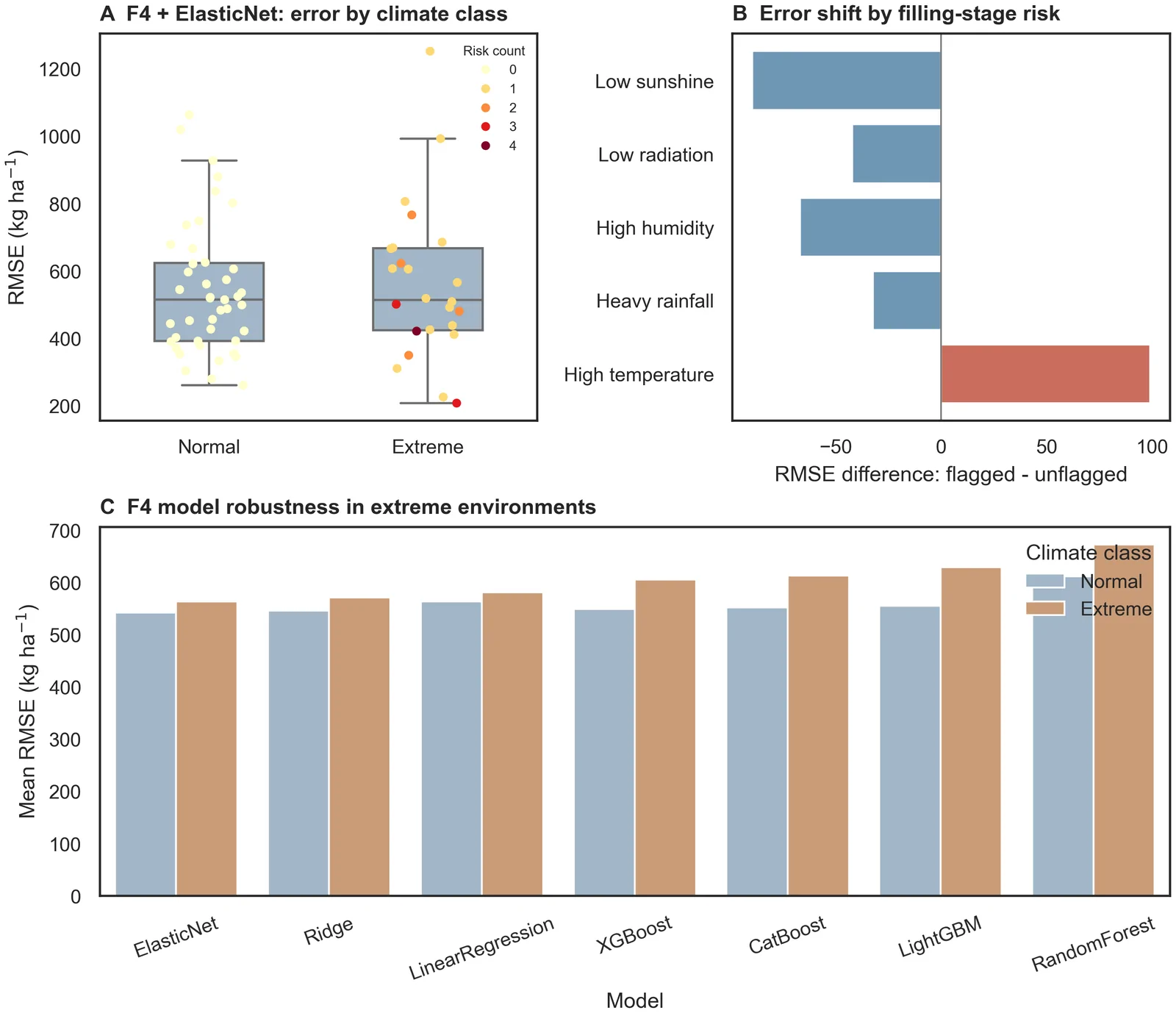
Prediction robustness under climate-risk environments. **(A)** Error distribution comparison between normal and climate-risk environments. **(B)** Risk-specific RMSE differences. **(C)** Model performance under normal and climate-risk environments.

Among the 67 site-year environments, 24 were classified as extreme because they met at least one heading-to-maturity climate-risk criterion (**Figure 8**; **Table 5**; **Supplementary Tables 10**, **11**, **18**). For F4 with ElasticNet, mean RMSE was 542.44 kg ha^−1^ in normal environments and 563.59 kg ha^−1^ in extreme environments, an increase of 21.15 kg ha^−1^; this difference was not statistically significant in either Welch or Mann-Whitney tests. Using a stricter definition requiring two or more risk flags did not produce a consistent performance penalty.

**Table 5:** Performance under normal and extreme environments.

Climate class	Environments	Mean R^2^	Median R^2^	Mean RMSE	Median RMSE	Mean MAE	Top-20% recall
Extreme	24	-0.046	0.248	563.588	513.5	465.692	0.611
Normal	43	-0.072	0.196	542.441	514.312	448.257	0.441

Risk-type analyses therefore did not support the stronger claim that the model generally failed under climate-risk environments. Instead, high-temperature environments showed a descriptive RMSE increase of 98.92 kg ha^−1^, while other risk classes showed weaker or opposite shifts. Top-20% recall was not lower in flagged environments. We therefore revised this section to describe risk-specific uncertainty rather than broad climate-risk degradation.

### 3.7 Model interpretation identified scenario-specific information priorities

**Figure 9:**
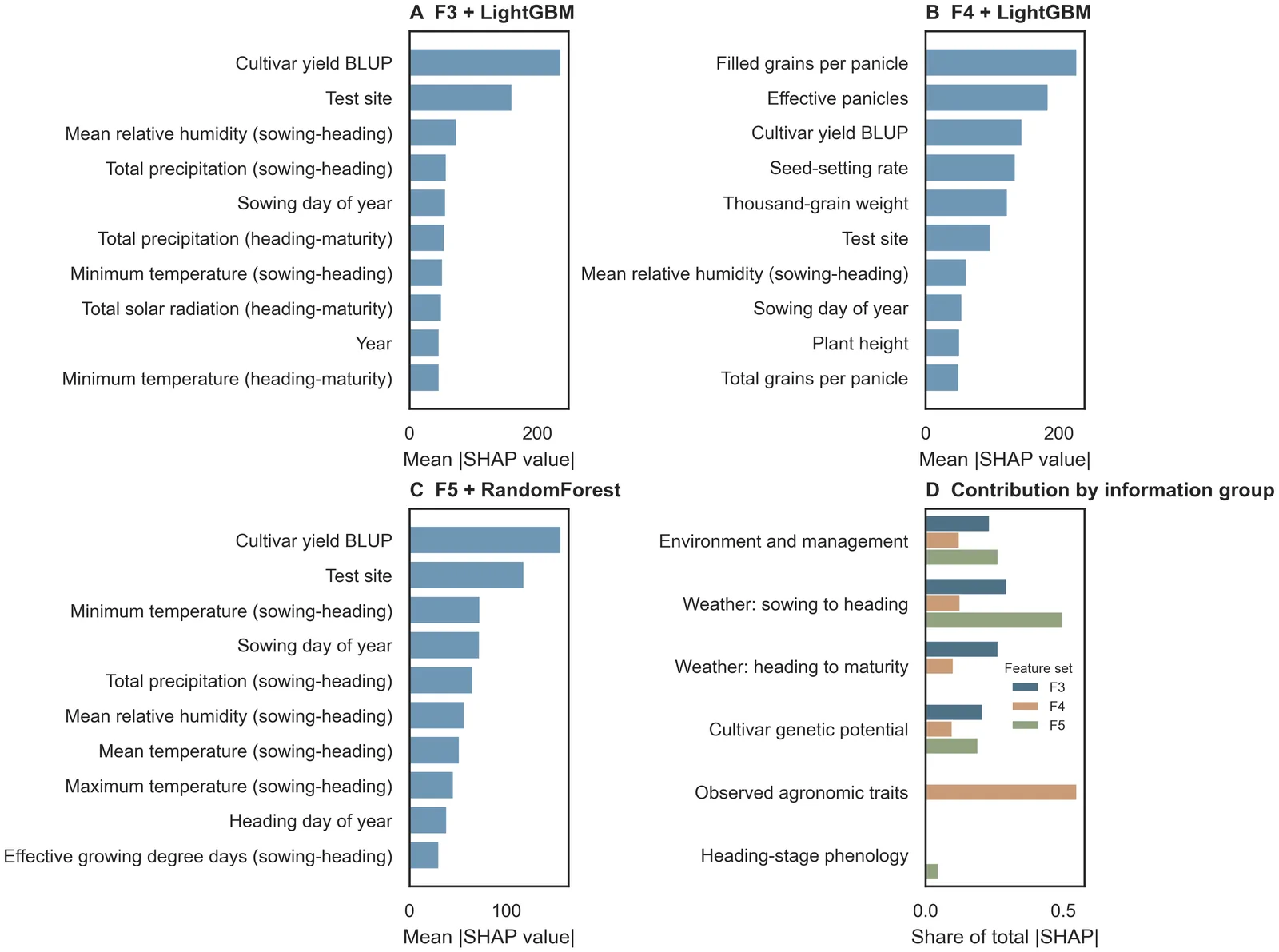
Model interpretation across information scenarios. **(A–C)** Leading features ranked by mean absolute SHAP value for the interpreted prediction scenarios. **(D)** Relative SHAP contribution of information groups.

SHAP analysis clarified why the prediction scenarios behaved differently (**Figure 9**; **Table 6**; **Supplementary Tables 12**, **22**, **23**). In the F3 climate-plus-genetic-potential scenario, cultivar yield BLUP was the leading feature, followed by test site and sowing-to-heading weather variables. Cross-algorithm SHAP comparison showed moderate agreement for F3 (Spearman rank correlation = 0.684; eight of the top 10 features overlapped between LightGBM and random forest).

**Table 6:** Feature-level SHAP importance.

Feature set	Model	Rank	Feature	Information group	Mean absolute SHAP value
F3	LightGBM	1	Cultivar yield BLUP	Cultivar genetic potential	236.811
F3	LightGBM	2	Test site	Environment and management	160.378
F3	LightGBM	3	Mean relative humidity: sowing to heading	Weather: sowing to heading	73.626
F3	LightGBM	4	Total precipitation: sowing to heading	Weather: sowing to heading	57.651
F3	LightGBM	5	Sowing day of year	Environment and management	56.657
F4	LightGBM	1	Filled grains per panicle	Observed agronomic traits	227.383
F4	LightGBM	2	Effective panicles	Observed agronomic traits	184.191
F4	LightGBM	3	Cultivar yield BLUP	Cultivar genetic potential	144.931
F4	LightGBM	4	Seed-setting rate	Observed agronomic traits	134.659
F4	LightGBM	5	Thousand-grain weight	Observed agronomic traits	123.126
F5	Random forest	1	Cultivar yield BLUP	Cultivar genetic potential	156.387
F5	Random forest	2	Test site	Environment and management	118.279
F5	Random forest	3	Mean minimum temperature: sowing to heading	Weather: sowing to heading	72.719
F5	Random forest	4	Sowing day of year	Environment and management	72.652
F5	Random forest	5	Total precipitation: sowing to heading	Weather: sowing to heading	65.621

In F4, observed agronomic traits dominated the fitted model and accounted for 55.1% of total absolute SHAP attribution. Filled grains per panicle, effective panicles, seed-setting rate and thousand-grain weight were prominent, but their exact rank varied by algorithm. LightGBM and random forest still shared eight of the top 10 F4 features and had a Spearman rank correlation of 0.728, supporting a stable group-level conclusion while discouraging overinterpretation of individual climate-variable ranks.

In the F5 heading-stage update scenario, cultivar yield BLUP was again the leading feature. Sowing-to-heading weather and sowing timing were consistently important across algorithms, with the strongest SHAP stability among the three interpreted scenarios (Spearman rank correlation = 0.918; eight of the top 10 features overlapped). Climate features formed several highly correlated clusters, so climate attribution was interpreted at the metric or growth-stage group level rather than as independent effects of single variables.

## 4 Discussion

This study established a scenario-aware framework for rice yield prediction that separates internal accuracy from deployment-oriented generalization. The main contribution is not a single universally optimal predictor. Instead, the framework identifies which information sources become useful at different crop-development stages and shows how model credibility changes when the deployment target shifts from related records to future years, new sites or unseen site-year combinations. This distinction extends earlier crop-yield modeling studies that demonstrated the potential of machine learning and crop-model integration across large spatial and temporal domains (Leng and Hall, 2020; Shahhosseini et al., 2021; Cao et al., 2021) by showing that model evaluation must also reflect the intended decision point and extrapolation target.

The leakage-controlled cultivar BLUP feature provided a bridge between multi-environment trial analysis and machine learning. BLUP has long been used to summarize cultivar performance in regional yield trials (Piepho, 1994), and recent studies have shown that BLUP and machine learning can provide complementary information in crop-prediction settings (Kick and Washburn, 2023). This is consistent with the broader development of reaction-norm models and environment-informed prediction strategies for multi-environment trials (Jarquín et al., 2014; Westhues et al., 2022). Cultivar yield BLUP was the leading SHAP feature in both F3 and F5, indicating that historical performance remains informative when only climate, management and heading-stage information are available. Its role changed in F4, where observed agronomic traits dominated model attribution. This shift is biologically plausible: cultivar BLUP summarizes expected performance from historical trials, whereas traits such as filled grains per panicle, effective panicles and seed-setting rate capture the realized outcome of the current growing season more directly. The two information sources should therefore be viewed as complementary rather than competing (Yue et al., 2026). Historical genetic potential is especially useful before detailed field observations become available; later field surveys partly replace that prior information with season-specific evidence.

The identity of the best-performing algorithm also depended on the validation setting. LightGBM performed best for F4 under random cross-validation, whereas ElasticNet was preferred under leave-one-year-out, forward-year and leave-one-site-out evaluation. This pattern is consistent with the broader warning that random cross-validation can reward models that reproduce spatial or temporal structure already represented in the training data rather than models that extrapolate reliably (Roberts et al., 2017; Meyer et al., 2019; Sweet et al., 2023). A plausible explanation is that flexible tree-based models can exploit nonlinearities and local interactions when training and test records share related environments, while a regularized linear model may be less sensitive to distribution shifts when extrapolating to new years or sites. The correlated climate variables and strongly informative yield-component traits may further favor a parsimonious, shrinkage-based model under external transfer (Liang et al., 2026). A nested tuning check for F4 in one temporal and one spatial deployment setting did not overturn this pattern: after inner-CV tuning, ElasticNet retained slightly higher mean R2 than LightGBM in both forward-year and leave-one-site-out validation. We therefore present the algorithm ranking as a robustness pattern rather than as proof of inherent superiority of any single method.

The generalization gap is itself an important result. Temporal transfer was challenged by substantial cultivar turnover, and new-site evaluation removed the direct utility of site-specific patterns learned from the training data. Related multi-environment studies have likewise emphasized that prediction quality depends on how environmental information and target environments are represented during training (Jarquín et al., 2014; Westhues et al., 2022). The sensitivity analysis showed that the best forward-year result did not depend on the numeric year feature, ruling out a simple time-trend explanation for F4 performance. These findings support a stricter reporting standard for agricultural machine learning: random cross-validation should be accompanied by validation schemes that reflect the intended deployment domain.

Generalization to unseen site-year combinations remained the most difficult task. Although 42 of 67 environments had positive R^2^ values and the median R^2^ was 0.213, a small number of severe failures reduced the mean to -0.063. Climate-risk analysis further showed that prediction errors increased under adverse heading-to-maturity conditions, especially high-temperature environments. This pattern is agronomically plausible. High temperature can induce spikelet sterility at flowering (Satake and Yoshida, 1978; Jagadish et al., 2007), disrupt reproductive development (Wu et al., 2016) and reduce rice yield as night temperatures rise (Peng et al., 2004). Similar concerns apply more broadly to global crop production, for which historical warming has already affected crop yields (Lobell et al., 2011) and further temperature increases are associated with measurable yield losses (Zhao et al., 2017; Petropoulos et al., 2025). Our analysis does not establish causal effects of individual weather variables, but it identifies climate-risk conditions under which automated predictions require additional scrutiny. These observations support the development of uncertainty estimates, environment-similarity diagnostics and automated warnings for conditions that fall outside the well-represented training domain.

Model interpretation is important in this context because deployment decisions require more than a ranking of predictive algorithms. Agricultural ML studies increasingly use interpretability methods to identify influential predictors and communicate model behavior to domain users (Ryo, 2022). In the present study, SHAP analysis did not establish causal determinants of yield, but it clarified how the fitted models redistributed attribution across cultivar history, weather, management and field-survey traits as the prediction scenario changed. This type of scenario-specific explanation is especially useful when model outputs are used to guide additional field inspection rather than to replace agronomic judgement.

This study has several limitations. First, the trial network covered 10 sites and 7 years in South China, so the models should be evaluated in additional regions and seasons before broader deployment. Second, the extreme-environment analysis was based on empirical quantile thresholds within this dataset. The 10% threshold was used to flag the most unusual site-year environments while retaining enough environments for comparison, but it should not be interpreted as a universal physiological threshold. Third, F4 depends on observed agronomic traits and therefore represents later-stage estimation rather than early forecasting. Future work should integrate remote sensing, soil information, genomic markers and uncertainty quantification to improve transferability and support climate-resilient variety evaluation.

## 5 Conclusions

We developed and evaluated a deployment-oriented framework for rice yield prediction in South China. Stage-specific climate information and leakage-controlled cultivar potential provided useful information before detailed field observations became available, whereas field-survey traits produced the strongest accuracy in a later-stage estimation scenario. Random cross-validation substantially overstated performance relative to future-year, new-site and new-site-year deployment settings.

For practical deployment, the framework supports a tiered strategy. Historical cultivar performance and stage-specific climate information can provide an initial estimate, heading-stage observations can update the prediction and targeted surveys of yield-component traits can improve pre-harvest estimation. The results also show where caution is required. New cultivars, low within-environment yield variance and climate-risk environments can weaken ranking reliability. More broadly, agricultural machine-learning studies should align validation design, feature timing and interpretation with the decision setting they intend to support.

## Data Availability

The raw yield, phenological and agronomic trait records were extracted from official national variety registration trial reports for the Southern Rice Region of China, which are compiled and administered by the National Agricultural Technology Extension and Service Center under the Ministry of Agriculture and Rural Affairs. These records are not owned by the authors, and redistribution of the complete raw dataset is restricted by the data-sharing policies of the issuing agency. Aggregated and processed data supporting the findings of this study, sufficient to reproduce the analyses, can be made available by the corresponding author upon reasonable request and with permission from the data-issuing agency where applicable. Meteorological data are not subject to these restrictions and are freely available from the China Meteorological Data Service Centre (http://data.cma.cn). Requests to access these datasets should be directed to Yongzhu Liu, lively@scau.edu.cn.
