# Supplementary material for: Deployment-oriented evaluation of stage-specific information for rice yield prediction across years, sites and site-year environments in southern China
Source: Front Plant Sci. 2026 Sep 8;17:1915306. doi: 10.3389/fpls.2026.1915306 (PMC13593899; doi:10.3389/fpls.2026.1915306)
Supplement: Supplementary file 1 [file DataSheet1.docx]

Supplementary Material

# 1 Supplementary Figures and Tables

## 1.1 Supplementary Figures

**Supplementary Figure 1.** Pearson correlations among stage-specific weather features. SH, sowing-heading; HM, heading-maturity; SM, sowing-maturity.

**Supplementary Figure 2.** Difference in mean absolute SHAP value between extreme and normal environments for the F4 LightGBM model.

**Supplementary Figure 3.** Partial dependence of the three leading continuous predictors in each interpretation scenario.

**Supplementary Figure 4.** Sensitivity of forward-year predictions to removal of the numeric Year feature. (A) Mean R^2^ for the best retained-Year model in each feature set. (B) Corresponding mean RMSE. (C) Year-specific change in R^2^ after removing Year.

## **Supplementary Figure 5.** Revision diagnostics for deployment-oriented interpretation. (A) R2 depends on within-environment variance(rho = 0.41). (B) Climate-risk sensitivity. (C) Forward-year performance by cultivar history.

## 1.2 Supplementary Table

**Supplementary Table 1.** BLUP feature definitions

| Feature | Role | Strict validation rule |
| --- | --- | --- |
| Cultivar yield BLUP | F3 core genetic-potential feature | recalculate from training fold only |
| GrowthDays BLUP value | optional phenology genetic-potential feature | recalculate from training fold only |
| DTH BLUP value | optional heading-time genetic-potential feature | recalculate from training fold only |
| DGM BLUP value | optional grain-filling-duration genetic-potential feature | recalculate from training fold only |
| SSR BLUP value | optional seed-setting genetic-potential feature | recalculate from training fold only |
| TGW BLUP value | optional grain-weight genetic-potential feature | recalculate from training fold only |

**Supplementary Table 2.** Random five-fold cross-validation results

| Feature set | Model | Folds | Mean R² | SD of R² | Mean RMSE | SD of RMSE | Mean MAE | SD of MAE | Mean normalized RMSE | SD of normalized RMSE | Mean Spearman correlation | SD of Spearman correlation | Mean top-20% recall | SD of top-20% recall |
| --- | --- | --- | --- | --- | --- | --- | --- | --- | --- | --- | --- | --- | --- | --- |
| F0 | Environment random-effect LMM | 5 | 0.402 | 0.024 | 667.431 | 14.214 | 520.043 | 15.388 | 0.089 | 0.002 | 0.583 | 0.019 | 0.589 | 0.024 |
| F0 | Environment mean | 5 | 0.402 | 0.026 | 667.635 | 14.001 | 519.89 | 15.238 | 0.089 | 0.002 | 0.583 | 0.019 | 0.589 | 0.034 |
| F0 | Overall mean | 5 | -0.003 | 0.003 | 864.786 | 24.543 | 668.594 | 23.665 | 0.115 | 0.003 |  |  | 0.185 | 0.039 |
| F1 | LightGBM | 5 | 0.662 | 0.027 | 501.433 | 8.574 | 377.947 | 7.881 | 0.067 | 0.001 | 0.776 | 0.01 | 0.688 | 0.026 |
| F1 | Random forest | 5 | 0.658 | 0.027 | 504.051 | 7.522 | 379.906 | 7.762 | 0.067 | 0.001 | 0.776 | 0.011 | 0.68 | 0.021 |
| F1 | XGBoost | 5 | 0.654 | 0.025 | 507.526 | 6.62 | 387.81 | 9.519 | 0.068 | 0.001 | 0.769 | 0.011 | 0.683 | 0.027 |
| F1 | CatBoost | 5 | 0.642 | 0.022 | 516.289 | 7.859 | 396.714 | 12.286 | 0.069 | 0.001 | 0.763 | 0.01 | 0.671 | 0.032 |
| F1 | LinearRegression | 5 | 0.344 | 0.047 | 698.714 | 7.928 | 537.65 | 8.298 | 0.093 | 0.001 | 0.549 | 0.022 | 0.544 | 0.048 |
| F1 | Ridge | 5 | 0.343 | 0.043 | 699.096 | 6.906 | 539.186 | 8.649 | 0.093 | 0.001 | 0.543 | 0.021 | 0.54 | 0.055 |
| F1 | ElasticNet | 5 | 0.342 | 0.042 | 699.804 | 6.685 | 540.189 | 8.884 | 0.093 | 0.001 | 0.54 | 0.021 | 0.544 | 0.054 |
| F2 | Random forest | 5 | 0.701 | 0.022 | 471.908 | 5.379 | 346.07 | 6.971 | 0.063 | 0 | 0.808 | 0.012 | 0.728 | 0.033 |
| F2 | LightGBM | 5 | 0.699 | 0.021 | 472.985 | 9.81 | 349.776 | 8.124 | 0.063 | 0.001 | 0.802 | 0.014 | 0.717 | 0.029 |
| F2 | XGBoost | 5 | 0.683 | 0.017 | 485.536 | 8.081 | 368.555 | 5.814 | 0.065 | 0.001 | 0.792 | 0.011 | 0.7 | 0.022 |
| F2 | CatBoost | 5 | 0.668 | 0.014 | 497.269 | 5.372 | 382.419 | 6.338 | 0.066 | 0.001 | 0.784 | 0.009 | 0.7 | 0.007 |
| F2 | LinearRegression | 5 | 0.362 | 0.042 | 688.698 | 4.814 | 527.611 | 8.977 | 0.092 | 0.001 | 0.551 | 0.019 | 0.526 | 0.046 |
| F2 | Ridge | 5 | 0.356 | 0.037 | 692.313 | 4.046 | 531.907 | 9.98 | 0.092 | 0.001 | 0.547 | 0.018 | 0.536 | 0.046 |
| F2 | ElasticNet | 5 | 0.354 | 0.036 | 693.581 | 4.198 | 533.496 | 10.271 | 0.093 | 0.001 | 0.546 | 0.018 | 0.533 | 0.049 |
| F3 | LightGBM | 5 | 0.755 | 0.024 | 426.969 | 12.93 | 316.434 | 7.031 | 0.057 | 0.001 | 0.84 | 0.019 | 0.725 | 0.036 |
| F3 | Random forest | 5 | 0.752 | 0.023 | 429.688 | 9.032 | 314.347 | 7.074 | 0.057 | 0.001 | 0.847 | 0.017 | 0.75 | 0.021 |
| F3 | XGBoost | 5 | 0.735 | 0.019 | 444.392 | 7.896 | 335.011 | 3.717 | 0.059 | 0.001 | 0.827 | 0.017 | 0.705 | 0.035 |
| F3 | CatBoost | 5 | 0.724 | 0.018 | 452.92 | 7.803 | 346.426 | 6.24 | 0.06 | 0.001 | 0.823 | 0.016 | 0.707 | 0.028 |
| F3 | LinearRegression | 5 | 0.426 | 0.057 | 652.664 | 15.714 | 498.781 | 9.38 | 0.087 | 0.002 | 0.608 | 0.034 | 0.551 | 0.038 |
| F3 | Ridge | 5 | 0.421 | 0.054 | 655.937 | 13.39 | 502.974 | 9.681 | 0.087 | 0.002 | 0.607 | 0.034 | 0.548 | 0.04 |
| F3 | ElasticNet | 5 | 0.418 | 0.053 | 657.273 | 12.73 | 504.702 | 9.532 | 0.088 | 0.002 | 0.606 | 0.034 | 0.54 | 0.036 |
| F4 | LightGBM | 5 | 0.844 | 0.018 | 340.593 | 11.43 | 252.518 | 9.204 | 0.045 | 0.002 | 0.9 | 0.015 | 0.803 | 0.028 |
| F4 | XGBoost | 5 | 0.824 | 0.023 | 360.948 | 14.686 | 274.099 | 9.862 | 0.048 | 0.002 | 0.888 | 0.02 | 0.781 | 0.023 |
| F4 | CatBoost | 5 | 0.806 | 0.018 | 379.414 | 9.575 | 292.671 | 10.048 | 0.051 | 0.001 | 0.879 | 0.016 | 0.77 | 0.021 |
| F4 | Random forest | 5 | 0.802 | 0.02 | 383.255 | 8.958 | 276.737 | 4.686 | 0.051 | 0.001 | 0.89 | 0.013 | 0.77 | 0.025 |
| F4 | LinearRegression | 5 | 0.635 | 0.036 | 520.508 | 19.277 | 400.696 | 12.843 | 0.069 | 0.003 | 0.773 | 0.027 | 0.666 | 0.046 |
| F4 | Ridge | 5 | 0.634 | 0.033 | 521.848 | 17.181 | 402.363 | 11.161 | 0.07 | 0.003 | 0.773 | 0.025 | 0.657 | 0.042 |
| F4 | ElasticNet | 5 | 0.633 | 0.033 | 522.605 | 16.708 | 403.266 | 10.881 | 0.07 | 0.002 | 0.771 | 0.025 | 0.66 | 0.048 |
| F5 | Random forest | 5 | 0.723 | 0.023 | 453.926 | 5.666 | 334.036 | 3.204 | 0.061 | 0.001 | 0.83 | 0.013 | 0.724 | 0.029 |
| F5 | LightGBM | 5 | 0.719 | 0.019 | 457.25 | 4.43 | 339.665 | 2.183 | 0.061 | 0 | 0.821 | 0.016 | 0.707 | 0.033 |
| F5 | XGBoost | 5 | 0.705 | 0.02 | 468.335 | 2.736 | 355.234 | 2.205 | 0.062 | 0 | 0.811 | 0.013 | 0.68 | 0.03 |
| F5 | CatBoost | 5 | 0.695 | 0.017 | 476.572 | 6.906 | 365.739 | 7.869 | 0.064 | 0.001 | 0.806 | 0.011 | 0.665 | 0.033 |
| F5 | LinearRegression | 5 | 0.4 | 0.059 | 667.708 | 16.077 | 511.539 | 7.792 | 0.089 | 0.002 | 0.601 | 0.036 | 0.539 | 0.055 |
| F5 | Ridge | 5 | 0.394 | 0.055 | 670.981 | 12.818 | 515.196 | 6.419 | 0.089 | 0.002 | 0.596 | 0.035 | 0.531 | 0.048 |
| F5 | ElasticNet | 5 | 0.391 | 0.054 | 672.844 | 11.814 | 517.049 | 5.994 | 0.09 | 0.002 | 0.593 | 0.035 | 0.536 | 0.042 |

**Supplementary Table 3.** Leave-one-year-out validation results

| Feature set | Model | Year splits | Mean R² | SD of R² | Mean RMSE | SD of RMSE | Mean MAE | SD of MAE | Mean normalized RMSE | SD of normalized RMSE | Mean Spearman correlation | SD of Spearman correlation | Mean top-20% recall | SD of top-20% recall |
| --- | --- | --- | --- | --- | --- | --- | --- | --- | --- | --- | --- | --- | --- | --- |
| F0 | Environment random-effect LMM | 7 | -0.044 | 0.052 | 867.565 | 76.733 | 672.614 | 68.247 | 0.116 | 0.008 |  |  | 0.207 | 0.027 |
| F0 | Environment mean | 7 | -0.046 | 0.046 | 868.063 | 74.222 | 672.127 | 67.717 | 0.116 | 0.008 |  |  | 0.207 | 0.027 |
| F0 | Overall mean | 7 | -0.046 | 0.046 | 868.063 | 74.222 | 672.127 | 67.717 | 0.116 | 0.008 |  |  | 0.207 | 0.027 |
| F1 | ElasticNet | 7 | 0.168 | 0.378 | 755.57 | 156.004 | 590.79 | 108.24 | 0.101 | 0.022 | 0.444 | 0.204 | 0.486 | 0.157 |
| F1 | Ridge | 7 | 0.156 | 0.393 | 759.883 | 160.845 | 594.249 | 110.761 | 0.101 | 0.022 | 0.442 | 0.205 | 0.481 | 0.166 |
| F1 | LightGBM | 7 | 0.13 | 0.317 | 777.202 | 133.06 | 604.354 | 83.545 | 0.104 | 0.019 | 0.38 | 0.227 | 0.472 | 0.167 |
| F1 | Random forest | 7 | 0.127 | 0.287 | 782.072 | 129.167 | 612.817 | 88.622 | 0.104 | 0.018 | 0.394 | 0.21 | 0.499 | 0.154 |
| F1 | XGBoost | 7 | 0.118 | 0.293 | 784.45 | 119.421 | 613.011 | 80.513 | 0.105 | 0.017 | 0.38 | 0.207 | 0.468 | 0.162 |
| F1 | LinearRegression | 7 | 0.116 | 0.456 | 774.494 | 181.345 | 606.112 | 118.972 | 0.103 | 0.025 | 0.448 | 0.2 | 0.479 | 0.164 |
| F1 | CatBoost | 7 | 0.09 | 0.285 | 799.215 | 127.057 | 622.456 | 87.536 | 0.107 | 0.017 | 0.364 | 0.182 | 0.481 | 0.128 |
| F2 | ElasticNet | 7 | 0.138 | 0.387 | 769.706 | 157.061 | 596.133 | 103.977 | 0.103 | 0.022 | 0.414 | 0.213 | 0.5 | 0.161 |
| F2 | CatBoost | 7 | 0.135 | 0.211 | 782.355 | 93.821 | 611.665 | 66.414 | 0.104 | 0.013 | 0.383 | 0.137 | 0.477 | 0.112 |
| F2 | LightGBM | 7 | 0.127 | 0.248 | 784.17 | 111.84 | 620.171 | 77.572 | 0.105 | 0.015 | 0.391 | 0.182 | 0.478 | 0.151 |
| F2 | Ridge | 7 | 0.124 | 0.4 | 774.826 | 160.006 | 599.201 | 104.614 | 0.103 | 0.022 | 0.41 | 0.214 | 0.497 | 0.166 |
| F2 | Random forest | 7 | 0.124 | 0.232 | 788.318 | 121.491 | 613.798 | 85.751 | 0.105 | 0.016 | 0.42 | 0.145 | 0.466 | 0.135 |
| F2 | XGBoost | 7 | 0.12 | 0.263 | 785.367 | 108.81 | 616.554 | 76.528 | 0.105 | 0.015 | 0.392 | 0.166 | 0.475 | 0.123 |
| F2 | LinearRegression | 7 | 0.057 | 0.472 | 800.528 | 179.929 | 614.24 | 110.339 | 0.107 | 0.025 | 0.394 | 0.196 | 0.474 | 0.161 |
| F3 | CatBoost | 7 | 0.151 | 0.228 | 774.592 | 105.011 | 606.66 | 73.251 | 0.103 | 0.014 | 0.414 | 0.137 | 0.475 | 0.144 |
| F3 | Random forest | 7 | 0.148 | 0.226 | 777.117 | 116.759 | 609.396 | 84.473 | 0.104 | 0.015 | 0.432 | 0.155 | 0.492 | 0.14 |
| F3 | ElasticNet | 7 | 0.146 | 0.367 | 767.241 | 152.13 | 594.048 | 107.449 | 0.102 | 0.021 | 0.424 | 0.208 | 0.492 | 0.14 |
| F3 | Ridge | 7 | 0.132 | 0.382 | 772.596 | 156.238 | 596.63 | 109.209 | 0.103 | 0.022 | 0.421 | 0.212 | 0.501 | 0.141 |
| F3 | XGBoost | 7 | 0.131 | 0.265 | 780.813 | 117.001 | 614.871 | 84.72 | 0.104 | 0.016 | 0.407 | 0.166 | 0.494 | 0.131 |
| F3 | LightGBM | 7 | 0.097 | 0.268 | 796.855 | 118.269 | 627.257 | 80.148 | 0.106 | 0.016 | 0.389 | 0.158 | 0.465 | 0.144 |
| F3 | LinearRegression | 7 | 0.067 | 0.454 | 796.991 | 177.233 | 611.026 | 116.557 | 0.106 | 0.025 | 0.412 | 0.2 | 0.489 | 0.156 |
| F4 | ElasticNet | 7 | 0.474 | 0.189 | 605.263 | 89.445 | 468.191 | 69.399 | 0.081 | 0.013 | 0.668 | 0.129 | 0.587 | 0.136 |
| F4 | Ridge | 7 | 0.464 | 0.186 | 611.094 | 86.083 | 472.602 | 66.131 | 0.082 | 0.013 | 0.662 | 0.126 | 0.576 | 0.141 |
| F4 | XGBoost | 7 | 0.449 | 0.152 | 621.856 | 67.587 | 487.06 | 47.83 | 0.083 | 0.01 | 0.652 | 0.092 | 0.584 | 0.129 |
| F4 | LightGBM | 7 | 0.44 | 0.138 | 628.12 | 60.706 | 494.183 | 45.072 | 0.084 | 0.009 | 0.642 | 0.079 | 0.573 | 0.134 |
| F4 | CatBoost | 7 | 0.436 | 0.156 | 629.943 | 75.902 | 496.423 | 52.657 | 0.084 | 0.011 | 0.639 | 0.099 | 0.592 | 0.137 |
| F4 | LinearRegression | 7 | 0.412 | 0.178 | 641.376 | 76.173 | 497.492 | 54.709 | 0.086 | 0.011 | 0.628 | 0.115 | 0.55 | 0.161 |
| F4 | Random forest | 7 | 0.309 | 0.181 | 698.326 | 89.943 | 548.537 | 64.145 | 0.093 | 0.012 | 0.571 | 0.118 | 0.533 | 0.157 |
| F5 | ElasticNet | 7 | 0.119 | 0.34 | 782.51 | 143.748 | 606.747 | 107.135 | 0.104 | 0.02 | 0.431 | 0.207 | 0.479 | 0.148 |
| F5 | Ridge | 7 | 0.111 | 0.355 | 784.786 | 148.718 | 607.719 | 109.658 | 0.105 | 0.021 | 0.431 | 0.211 | 0.488 | 0.145 |
| F5 | LinearRegression | 7 | 0.084 | 0.377 | 795.324 | 148.862 | 614.477 | 104.264 | 0.106 | 0.021 | 0.437 | 0.205 | 0.48 | 0.149 |
| F5 | Random forest | 7 | 0.01 | 0.28 | 836.504 | 134.46 | 652.974 | 103.79 | 0.112 | 0.018 | 0.346 | 0.181 | 0.423 | 0.102 |
| F5 | CatBoost | 7 | -0.048 | 0.302 | 859.056 | 130.062 | 666.973 | 94.119 | 0.115 | 0.017 | 0.327 | 0.165 | 0.415 | 0.088 |
| F5 | XGBoost | 7 | -0.108 | 0.375 | 880.328 | 161.009 | 679.609 | 123.943 | 0.117 | 0.021 | 0.298 | 0.185 | 0.404 | 0.142 |
| F5 | LightGBM | 7 | -0.14 | 0.333 | 895.657 | 143.464 | 694.404 | 106.328 | 0.119 | 0.019 | 0.261 | 0.169 | 0.342 | 0.143 |

**Supplementary Table 4.** Forward-year validation results

| Feature set | Model | Year splits | Mean R² | SD of R² | Mean RMSE | SD of RMSE | Mean MAE | SD of MAE | Mean normalized RMSE | SD of normalized RMSE | Mean Spearman correlation | SD of Spearman correlation | Mean top-20% recall | SD of top-20% recall |
| --- | --- | --- | --- | --- | --- | --- | --- | --- | --- | --- | --- | --- | --- | --- |
| F0 | Environment random-effect LMM | 6 | -0.05 | 0.048 | 878.167 | 56.717 | 685.216 | 50.715 | 0.117 | 0.006 |  |  | 0.203 | 0.028 |
| F0 | Environment mean | 6 | -0.061 | 0.06 | 882.325 | 50.513 | 687.515 | 48.21 | 0.118 | 0.005 |  |  | 0.203 | 0.028 |
| F0 | Overall mean | 6 | -0.061 | 0.06 | 882.325 | 50.513 | 687.515 | 48.21 | 0.118 | 0.005 |  |  | 0.203 | 0.028 |
| F1 | Random forest | 6 | 0.068 | 0.257 | 817.94 | 95.477 | 636.287 | 58.492 | 0.109 | 0.013 | 0.35 | 0.125 | 0.482 | 0.158 |
| F1 | CatBoost | 6 | 0.057 | 0.22 | 824.802 | 79.617 | 638.132 | 50.895 | 0.11 | 0.011 | 0.306 | 0.161 | 0.427 | 0.158 |
| F1 | LightGBM | 6 | 0.041 | 0.277 | 826.865 | 84.584 | 635.22 | 43.863 | 0.11 | 0.013 | 0.33 | 0.157 | 0.427 | 0.181 |
| F1 | ElasticNet | 6 | 0.031 | 0.439 | 819.31 | 149.806 | 635.863 | 100.835 | 0.11 | 0.022 | 0.388 | 0.145 | 0.434 | 0.169 |
| F1 | Ridge | 6 | 0.027 | 0.445 | 820.737 | 152.558 | 638.716 | 100.518 | 0.11 | 0.022 | 0.379 | 0.145 | 0.429 | 0.17 |
| F1 | XGBoost | 6 | 0.024 | 0.246 | 836.431 | 67.163 | 638.798 | 32.642 | 0.112 | 0.01 | 0.3 | 0.136 | 0.382 | 0.133 |
| F1 | LinearRegression | 6 | -0.057 | 0.438 | 860.636 | 151.438 | 657.248 | 93.047 | 0.115 | 0.022 | 0.38 | 0.149 | 0.399 | 0.175 |
| F2 | CatBoost | 6 | 0.106 | 0.214 | 802.113 | 78.019 | 627.925 | 52.581 | 0.107 | 0.011 | 0.362 | 0.171 | 0.454 | 0.174 |
| F2 | Random forest | 6 | 0.079 | 0.247 | 813.004 | 86.098 | 634.486 | 49.584 | 0.108 | 0.012 | 0.371 | 0.142 | 0.465 | 0.185 |
| F2 | XGBoost | 6 | -0.02 | 0.307 | 851.242 | 107.17 | 664.782 | 69.801 | 0.114 | 0.015 | 0.322 | 0.182 | 0.418 | 0.162 |
| F2 | ElasticNet | 6 | -0.03 | 0.57 | 833.805 | 186.28 | 643.233 | 139.204 | 0.112 | 0.027 | 0.375 | 0.176 | 0.483 | 0.195 |
| F2 | Ridge | 6 | -0.043 | 0.574 | 839.363 | 186.227 | 648.42 | 137.055 | 0.112 | 0.027 | 0.365 | 0.171 | 0.483 | 0.195 |
| F2 | LightGBM | 6 | -0.129 | 0.425 | 893.192 | 161.651 | 704.883 | 124.361 | 0.119 | 0.021 | 0.321 | 0.162 | 0.425 | 0.164 |
| F2 | LinearRegression | 6 | -0.312 | 0.597 | 951.378 | 183.698 | 739.635 | 149.376 | 0.127 | 0.026 | 0.385 | 0.174 | 0.412 | 0.181 |
| F3 | Random forest | 6 | 0.086 | 0.253 | 808.951 | 85.518 | 634.12 | 49.605 | 0.108 | 0.012 | 0.37 | 0.141 | 0.469 | 0.156 |
| F3 | CatBoost | 6 | 0.071 | 0.258 | 815.816 | 92.48 | 638.266 | 62.068 | 0.109 | 0.013 | 0.336 | 0.173 | 0.457 | 0.135 |
| F3 | XGBoost | 6 | 0.013 | 0.273 | 839.474 | 86.621 | 660.369 | 52.555 | 0.112 | 0.013 | 0.301 | 0.191 | 0.411 | 0.139 |
| F3 | LightGBM | 6 | -0.024 | 0.299 | 854.151 | 107.744 | 673.753 | 82.641 | 0.114 | 0.015 | 0.307 | 0.206 | 0.433 | 0.152 |
| F3 | ElasticNet | 6 | -0.033 | 0.496 | 843.608 | 161.202 | 649.474 | 117.664 | 0.113 | 0.023 | 0.36 | 0.188 | 0.48 | 0.181 |
| F3 | Ridge | 6 | -0.05 | 0.496 | 850.99 | 159.443 | 656.676 | 112.501 | 0.114 | 0.023 | 0.35 | 0.182 | 0.477 | 0.183 |
| F3 | LinearRegression | 6 | -0.389 | 0.576 | 984.909 | 191.451 | 764.323 | 144.072 | 0.131 | 0.026 | 0.339 | 0.166 | 0.415 | 0.179 |
| F4 | ElasticNet | 6 | 0.359 | 0.232 | 673.605 | 93.758 | 525.059 | 68.595 | 0.09 | 0.013 | 0.61 | 0.134 | 0.578 | 0.121 |
| F4 | Ridge | 6 | 0.349 | 0.23 | 679.442 | 88.87 | 530.617 | 63.578 | 0.091 | 0.013 | 0.601 | 0.132 | 0.564 | 0.116 |
| F4 | CatBoost | 6 | 0.319 | 0.214 | 697.283 | 90.115 | 546.476 | 55.574 | 0.093 | 0.012 | 0.57 | 0.131 | 0.519 | 0.142 |
| F4 | XGBoost | 6 | 0.318 | 0.198 | 698.227 | 75.194 | 550.685 | 54.993 | 0.093 | 0.011 | 0.562 | 0.13 | 0.526 | 0.132 |
| F4 | LightGBM | 6 | 0.223 | 0.31 | 739.905 | 133.923 | 583.028 | 109.918 | 0.099 | 0.017 | 0.535 | 0.156 | 0.522 | 0.094 |
| F4 | Random forest | 6 | 0.217 | 0.185 | 750.6 | 53.636 | 590.384 | 29.876 | 0.1 | 0.008 | 0.488 | 0.107 | 0.506 | 0.167 |
| F4 | LinearRegression | 6 | -0.036 | 0.613 | 844.006 | 237.27 | 668.373 | 189.947 | 0.112 | 0.03 | 0.524 | 0.15 | 0.452 | 0.151 |
| F5 | ElasticNet | 6 | 0.035 | 0.372 | 824.572 | 131.836 | 635.701 | 86.182 | 0.11 | 0.019 | 0.375 | 0.203 | 0.501 | 0.197 |
| F5 | Ridge | 6 | 0.025 | 0.383 | 828.256 | 135.974 | 640.041 | 87.155 | 0.111 | 0.02 | 0.373 | 0.204 | 0.503 | 0.199 |
| F5 | Random forest | 6 | -0.015 | 0.3 | 852.069 | 104.019 | 666.972 | 69.512 | 0.114 | 0.015 | 0.317 | 0.195 | 0.415 | 0.148 |
| F5 | CatBoost | 6 | -0.057 | 0.295 | 870.008 | 92.554 | 676.311 | 56.141 | 0.116 | 0.014 | 0.307 | 0.173 | 0.421 | 0.148 |
| F5 | LightGBM | 6 | -0.104 | 0.408 | 880.724 | 133.337 | 685.499 | 73.118 | 0.118 | 0.02 | 0.291 | 0.201 | 0.394 | 0.152 |
| F5 | XGBoost | 6 | -0.118 | 0.435 | 883.68 | 138.443 | 682.828 | 81.695 | 0.118 | 0.02 | 0.287 | 0.207 | 0.416 | 0.141 |
| F5 | LinearRegression | 6 | -0.335 | 0.587 | 964.019 | 207.794 | 733.326 | 138.352 | 0.129 | 0.027 | 0.316 | 0.191 | 0.431 | 0.181 |

**Supplementary Table 5.** Leave-one-site-out validation results

| Feature set | Model | Site splits | Mean R² | SD of R² | Mean RMSE | SD of RMSE | Mean MAE | SD of MAE | Mean normalized RMSE | SD of normalized RMSE | Mean Spearman correlation | SD of Spearman correlation | Mean top-20% recall | SD of top-20% recall |
| --- | --- | --- | --- | --- | --- | --- | --- | --- | --- | --- | --- | --- | --- | --- |
| F0 | Environment random-effect LMM | 10 | -0.236 | 0.316 | 837.268 | 281.358 | 681.342 | 251.204 | 0.112 | 0.035 |  |  | 0.189 | 0.123 |
| F0 | Environment mean | 10 | -0.245 | 0.315 | 841.013 | 284.018 | 683.639 | 253.088 | 0.113 | 0.036 |  |  | 0.189 | 0.123 |
| F0 | Overall mean | 10 | -0.245 | 0.315 | 841.013 | 284.018 | 683.639 | 253.088 | 0.113 | 0.036 |  |  | 0.189 | 0.123 |
| F1 | ElasticNet | 10 | -0.03 | 0.13 | 753.898 | 175.592 | 606.42 | 146.974 | 0.101 | 0.022 | 0.297 | 0.145 | 0.299 | 0.142 |
| F1 | Ridge | 10 | -0.04 | 0.129 | 756.565 | 171.986 | 607.927 | 142.676 | 0.101 | 0.021 | 0.289 | 0.138 | 0.3 | 0.143 |
| F1 | LinearRegression | 10 | -0.078 | 0.151 | 769.608 | 176.032 | 618.546 | 145.633 | 0.103 | 0.021 | 0.262 | 0.164 | 0.303 | 0.141 |
| F1 | Random forest | 10 | -0.154 | 0.266 | 800.996 | 234.784 | 657.55 | 211.64 | 0.107 | 0.028 | 0.227 | 0.152 | 0.299 | 0.123 |
| F1 | CatBoost | 10 | -0.189 | 0.271 | 813.49 | 240.355 | 672.816 | 218.625 | 0.109 | 0.029 | 0.236 | 0.189 | 0.258 | 0.143 |
| F1 | XGBoost | 10 | -0.236 | 0.231 | 828.289 | 223.959 | 681.354 | 203.575 | 0.111 | 0.028 | 0.183 | 0.17 | 0.311 | 0.176 |
| F1 | LightGBM | 10 | -0.331 | 0.29 | 857.117 | 234.203 | 704.066 | 221.831 | 0.114 | 0.027 | 0.179 | 0.101 | 0.276 | 0.132 |
| F2 | ElasticNet | 10 | -0.075 | 0.125 | 772.97 | 189.597 | 621.638 | 154.464 | 0.104 | 0.024 | 0.278 | 0.173 | 0.265 | 0.112 |
| F2 | Ridge | 10 | -0.093 | 0.122 | 779.262 | 187.359 | 625.295 | 150.515 | 0.104 | 0.024 | 0.268 | 0.177 | 0.266 | 0.111 |
| F2 | Random forest | 10 | -0.119 | 0.259 | 789.188 | 234.932 | 646.627 | 212.911 | 0.105 | 0.028 | 0.266 | 0.173 | 0.315 | 0.123 |
| F2 | LinearRegression | 10 | -0.146 | 0.099 | 800.119 | 197.245 | 640.414 | 154.642 | 0.107 | 0.026 | 0.23 | 0.212 | 0.239 | 0.143 |
| F2 | CatBoost | 10 | -0.179 | 0.321 | 803.26 | 229.552 | 659.11 | 214.273 | 0.107 | 0.027 | 0.285 | 0.179 | 0.345 | 0.152 |
| F2 | LightGBM | 10 | -0.211 | 0.267 | 815.486 | 215.925 | 667.835 | 200.082 | 0.109 | 0.026 | 0.25 | 0.17 | 0.312 | 0.135 |
| F2 | XGBoost | 10 | -0.236 | 0.297 | 829.262 | 251.976 | 683.704 | 233.014 | 0.111 | 0.03 | 0.253 | 0.158 | 0.314 | 0.117 |
| F3 | ElasticNet | 10 | -0.017 | 0.141 | 753.715 | 199.443 | 610.501 | 167.442 | 0.101 | 0.025 | 0.39 | 0.157 | 0.326 | 0.126 |
| F3 | Ridge | 10 | -0.036 | 0.13 | 760.503 | 197.259 | 615.075 | 163.864 | 0.102 | 0.025 | 0.378 | 0.159 | 0.313 | 0.129 |
| F3 | Random forest | 10 | -0.063 | 0.271 | 771.759 | 246.519 | 632.516 | 223.309 | 0.103 | 0.03 | 0.361 | 0.16 | 0.32 | 0.087 |
| F3 | LinearRegression | 10 | -0.083 | 0.093 | 779.117 | 199.792 | 628.109 | 162.438 | 0.104 | 0.026 | 0.352 | 0.181 | 0.313 | 0.134 |
| F3 | CatBoost | 10 | -0.093 | 0.293 | 778.829 | 241.257 | 639.848 | 221.764 | 0.104 | 0.029 | 0.384 | 0.142 | 0.334 | 0.11 |
| F3 | XGBoost | 10 | -0.15 | 0.292 | 796.679 | 234.813 | 655.772 | 216.289 | 0.106 | 0.028 | 0.379 | 0.148 | 0.354 | 0.111 |
| F3 | LightGBM | 10 | -0.156 | 0.315 | 797.603 | 235.253 | 654.164 | 215.805 | 0.107 | 0.029 | 0.346 | 0.155 | 0.334 | 0.134 |
| F4 | ElasticNet | 10 | 0.29 | 0.228 | 618.026 | 150.081 | 493.874 | 117.849 | 0.083 | 0.019 | 0.648 | 0.076 | 0.506 | 0.13 |
| F4 | Ridge | 10 | 0.278 | 0.233 | 623.053 | 151.264 | 498.777 | 119.032 | 0.083 | 0.019 | 0.642 | 0.078 | 0.497 | 0.128 |
| F4 | CatBoost | 10 | 0.265 | 0.227 | 634.215 | 180.671 | 521.188 | 155.692 | 0.085 | 0.022 | 0.615 | 0.099 | 0.469 | 0.092 |
| F4 | XGBoost | 10 | 0.257 | 0.201 | 640.621 | 181.284 | 525.572 | 156.974 | 0.086 | 0.023 | 0.614 | 0.08 | 0.495 | 0.108 |
| F4 | LightGBM | 10 | 0.25 | 0.2 | 641.792 | 168.919 | 524.456 | 143.547 | 0.086 | 0.022 | 0.61 | 0.082 | 0.474 | 0.117 |
| F4 | LinearRegression | 10 | 0.236 | 0.226 | 642.458 | 155.769 | 515.963 | 120.883 | 0.086 | 0.02 | 0.619 | 0.084 | 0.484 | 0.138 |
| F4 | Random forest | 10 | 0.12 | 0.264 | 702.036 | 233.33 | 578.346 | 210.162 | 0.094 | 0.028 | 0.504 | 0.117 | 0.405 | 0.118 |
| F5 | ElasticNet | 10 | -0.033 | 0.213 | 765.295 | 246.257 | 618.03 | 210.545 | 0.102 | 0.032 | 0.407 | 0.162 | 0.362 | 0.138 |
| F5 | Ridge | 10 | -0.047 | 0.211 | 770.87 | 247.771 | 622.111 | 211.083 | 0.103 | 0.032 | 0.401 | 0.166 | 0.344 | 0.138 |
| F5 | CatBoost | 10 | -0.114 | 0.295 | 789.546 | 254.75 | 643.111 | 227.881 | 0.105 | 0.031 | 0.371 | 0.151 | 0.343 | 0.101 |
| F5 | Random forest | 10 | -0.121 | 0.31 | 793.196 | 262.152 | 648.005 | 235.263 | 0.106 | 0.032 | 0.352 | 0.167 | 0.347 | 0.118 |
| F5 | LinearRegression | 10 | -0.154 | 0.312 | 812.797 | 295.738 | 659.996 | 258.285 | 0.109 | 0.038 | 0.37 | 0.179 | 0.325 | 0.12 |
| F5 | XGBoost | 10 | -0.184 | 0.344 | 817.618 | 284.023 | 666.392 | 256.937 | 0.109 | 0.035 | 0.335 | 0.164 | 0.338 | 0.083 |
| F5 | LightGBM | 10 | -0.219 | 0.347 | 827.561 | 276.68 | 679.429 | 254.409 | 0.111 | 0.034 | 0.334 | 0.18 | 0.33 | 0.109 |

**Supplementary Table 6.** Best models in leave-one-site-out validation

| Feature set | Model | Site splits | Mean R² | SD of R² | Mean RMSE | SD of RMSE | Mean MAE | SD of MAE | Mean normalized RMSE | SD of normalized RMSE | Mean Spearman correlation | SD of Spearman correlation | Mean top-20% recall | SD of top-20% recall |
| --- | --- | --- | --- | --- | --- | --- | --- | --- | --- | --- | --- | --- | --- | --- |
| F4 | ElasticNet | 10 | 0.29 | 0.228 | 618.026 | 150.081 | 493.874 | 117.849 | 0.083 | 0.019 | 0.648 | 0.076 | 0.506 | 0.13 |
| F3 | ElasticNet | 10 | -0.017 | 0.141 | 753.715 | 199.443 | 610.501 | 167.442 | 0.101 | 0.025 | 0.39 | 0.157 | 0.326 | 0.126 |
| F1 | ElasticNet | 10 | -0.03 | 0.13 | 753.898 | 175.592 | 606.42 | 146.974 | 0.101 | 0.022 | 0.297 | 0.145 | 0.299 | 0.142 |
| F5 | ElasticNet | 10 | -0.033 | 0.213 | 765.295 | 246.257 | 618.03 | 210.545 | 0.102 | 0.032 | 0.407 | 0.162 | 0.362 | 0.138 |
| F2 | ElasticNet | 10 | -0.075 | 0.125 | 772.97 | 189.597 | 621.638 | 154.464 | 0.104 | 0.024 | 0.278 | 0.173 | 0.265 | 0.112 |
| F0 | Environment random-effect LMM | 10 | -0.236 | 0.316 | 837.268 | 281.358 | 681.342 | 251.204 | 0.112 | 0.035 |  |  | 0.189 | 0.123 |

Supplementary Table 7 Leave-one-site-year-out validation results

| Feature set | Model | Environment splits | Mean R² | SD of R² | Mean RMSE | SD of RMSE | Mean MAE | SD of MAE | Mean normalized RMSE | SD of normalized RMSE | Mean Spearman correlation | SD of Spearman correlation | Mean top-20% recall | SD of top-20% recall |
| --- | --- | --- | --- | --- | --- | --- | --- | --- | --- | --- | --- | --- | --- | --- |
| F0 | Environment random-effect LMM | 67 | -0.954 | 1.67 | 784.88 | 320.505 | 660.195 | 300.17 | 0.105 | 0.041 |  |  | 0.195 | 0.137 |
| F0 | Environment mean | 67 | -0.958 | 1.667 | 786.059 | 319.874 | 660.62 | 298.871 | 0.105 | 0.041 |  |  | 0.195 | 0.137 |
| F0 | Overall mean | 67 | -0.958 | 1.667 | 786.059 | 319.874 | 660.62 | 298.871 | 0.105 | 0.041 |  |  | 0.195 | 0.137 |
| F1 | ElasticNet | 67 | -0.616 | 1.267 | 711.578 | 278.768 | 592.288 | 239.067 | 0.096 | 0.039 | 0.216 | 0.378 | 0.203 | 0.137 |
| F1 | Ridge | 67 | -0.635 | 1.3 | 715.868 | 284.4 | 596.394 | 243.997 | 0.096 | 0.04 | 0.213 | 0.38 | 0.199 | 0.135 |
| F1 | LinearRegression | 67 | -0.675 | 1.306 | 726.121 | 301.693 | 606.285 | 260.621 | 0.098 | 0.042 | 0.21 | 0.384 | 0.202 | 0.136 |
| F1 | Random forest | 67 | -0.745 | 1.344 | 730.82 | 277.724 | 609.738 | 247.044 | 0.098 | 0.039 | 0.176 | 0.378 | 0.228 | 0.176 |
| F1 | XGBoost | 67 | -0.803 | 1.367 | 744.767 | 255.675 | 620.547 | 228.88 | 0.1 | 0.037 | 0.159 | 0.387 | 0.225 | 0.156 |
| F1 | CatBoost | 67 | -0.829 | 1.288 | 758.214 | 286.855 | 633.582 | 254.119 | 0.102 | 0.04 | 0.166 | 0.38 | 0.224 | 0.157 |
| F1 | LightGBM | 67 | -0.85 | 1.519 | 749.497 | 276.914 | 621.104 | 242.747 | 0.101 | 0.04 | 0.169 | 0.368 | 0.248 | 0.171 |
| F2 | ElasticNet | 67 | -0.668 | 1.287 | 722.237 | 297.743 | 600.393 | 259.056 | 0.097 | 0.041 | 0.252 | 0.394 | 0.263 | 0.174 |
| F2 | Ridge | 67 | -0.703 | 1.36 | 728.493 | 305.432 | 605.31 | 266.069 | 0.098 | 0.042 | 0.25 | 0.394 | 0.26 | 0.186 |
| F2 | Random forest | 67 | -0.758 | 1.324 | 729.008 | 273.15 | 608.155 | 247.356 | 0.098 | 0.037 | 0.2 | 0.401 | 0.233 | 0.184 |
| F2 | LinearRegression | 67 | -0.774 | 1.491 | 743.095 | 332.285 | 617.837 | 291.65 | 0.1 | 0.046 | 0.23 | 0.409 | 0.275 | 0.191 |
| F2 | CatBoost | 67 | -0.842 | 1.388 | 739.392 | 261.625 | 617.112 | 235.976 | 0.1 | 0.037 | 0.224 | 0.383 | 0.264 | 0.194 |
| F2 | LightGBM | 67 | -0.926 | 1.479 | 754.544 | 260.871 | 628.224 | 234.282 | 0.102 | 0.038 | 0.175 | 0.375 | 0.229 | 0.179 |
| F2 | XGBoost | 67 | -0.938 | 1.543 | 754.107 | 257.598 | 627.955 | 232.505 | 0.102 | 0.037 | 0.189 | 0.383 | 0.239 | 0.175 |
| F3 | ElasticNet | 67 | -0.621 | 1.543 | 693.526 | 297.909 | 579.457 | 265.22 | 0.093 | 0.041 | 0.443 | 0.348 | 0.361 | 0.205 |
| F3 | Random forest | 67 | -0.653 | 1.325 | 702.893 | 274.442 | 587.787 | 249.991 | 0.094 | 0.038 | 0.395 | 0.346 | 0.342 | 0.211 |
| F3 | Ridge | 67 | -0.666 | 1.66 | 700.567 | 306.498 | 585.251 | 272.878 | 0.094 | 0.043 | 0.441 | 0.348 | 0.369 | 0.211 |
| F3 | CatBoost | 67 | -0.736 | 1.444 | 711.697 | 270.089 | 595.371 | 246.303 | 0.096 | 0.038 | 0.416 | 0.328 | 0.348 | 0.228 |
| F3 | LinearRegression | 67 | -0.749 | 1.807 | 717.458 | 330.833 | 600.529 | 297.083 | 0.097 | 0.046 | 0.432 | 0.352 | 0.363 | 0.209 |
| F3 | LightGBM | 67 | -0.846 | 1.562 | 727.529 | 269.809 | 607.184 | 243.464 | 0.098 | 0.038 | 0.384 | 0.328 | 0.349 | 0.208 |
| F3 | XGBoost | 67 | -0.848 | 1.534 | 728.835 | 261.439 | 608.904 | 238.705 | 0.098 | 0.037 | 0.38 | 0.337 | 0.332 | 0.217 |
| F4 | ElasticNet | 67 | -0.063 | 1.069 | 550.016 | 208.806 | 454.502 | 184.126 | 0.074 | 0.029 | 0.628 | 0.231 | 0.502 | 0.198 |
| F4 | Ridge | 67 | -0.088 | 1.102 | 555.527 | 211.016 | 459.151 | 185.979 | 0.075 | 0.029 | 0.626 | 0.232 | 0.51 | 0.192 |
| F4 | XGBoost | 67 | -0.097 | 0.928 | 569.674 | 206.426 | 474.737 | 188.927 | 0.077 | 0.029 | 0.608 | 0.215 | 0.505 | 0.178 |
| F4 | CatBoost | 67 | -0.138 | 0.984 | 574.605 | 216.871 | 483.046 | 197.86 | 0.077 | 0.031 | 0.618 | 0.235 | 0.486 | 0.177 |
| F4 | LinearRegression | 67 | -0.142 | 1.115 | 570.244 | 220.491 | 472.113 | 194.351 | 0.077 | 0.03 | 0.624 | 0.23 | 0.502 | 0.189 |
| F4 | LightGBM | 67 | -0.146 | 0.962 | 581.916 | 215.307 | 486.72 | 195.977 | 0.078 | 0.031 | 0.6 | 0.223 | 0.486 | 0.182 |
| F4 | Random forest | 67 | -0.332 | 1.031 | 633.542 | 238.08 | 532.316 | 216.614 | 0.085 | 0.033 | 0.537 | 0.284 | 0.423 | 0.211 |
| F5 | ElasticNet | 67 | -0.626 | 1.763 | 683.609 | 289.609 | 568.743 | 263.225 | 0.092 | 0.041 | 0.447 | 0.329 | 0.361 | 0.214 |
| F5 | Ridge | 67 | -0.653 | 1.844 | 686.753 | 295.75 | 571.918 | 269.21 | 0.092 | 0.041 | 0.446 | 0.333 | 0.358 | 0.214 |
| F5 | LinearRegression | 67 | -0.743 | 2.024 | 697.646 | 311.317 | 581.99 | 285.617 | 0.094 | 0.043 | 0.438 | 0.342 | 0.349 | 0.209 |
| F5 | Random forest | 67 | -0.861 | 1.581 | 742.27 | 305.094 | 619.009 | 278.212 | 0.1 | 0.041 | 0.363 | 0.356 | 0.316 | 0.224 |
| F5 | CatBoost | 67 | -1.03 | 1.889 | 756.763 | 303.663 | 630.475 | 276.843 | 0.102 | 0.042 | 0.379 | 0.363 | 0.339 | 0.218 |
| F5 | XGBoost | 67 | -1.041 | 1.837 | 771.096 | 317.506 | 642.495 | 288.132 | 0.104 | 0.044 | 0.376 | 0.352 | 0.334 | 0.208 |
| F5 | LightGBM | 67 | -1.202 | 2.146 | 789.616 | 329.004 | 659.338 | 302.138 | 0.106 | 0.045 | 0.351 | 0.346 | 0.346 | 0.217 |

**Supplementary Table 8.** Best models in leave-one-site-year-out validation

| Feature set | Model | Environment splits | Mean R² | SD of R² | Mean RMSE | SD of RMSE | Mean MAE | SD of MAE | Mean normalized RMSE | SD of normalized RMSE | Mean Spearman correlation | SD of Spearman correlation | Mean top-20% recall | SD of top-20% recall |
| --- | --- | --- | --- | --- | --- | --- | --- | --- | --- | --- | --- | --- | --- | --- |
| F4 | ElasticNet | 67 | -0.063 | 1.069 | 550.016 | 208.806 | 454.502 | 184.126 | 0.074 | 0.029 | 0.628 | 0.231 | 0.502 | 0.198 |
| F1 | ElasticNet | 67 | -0.616 | 1.267 | 711.578 | 278.768 | 592.288 | 239.067 | 0.096 | 0.039 | 0.216 | 0.378 | 0.203 | 0.137 |
| F3 | ElasticNet | 67 | -0.621 | 1.543 | 693.526 | 297.909 | 579.457 | 265.22 | 0.093 | 0.041 | 0.443 | 0.348 | 0.361 | 0.205 |
| F5 | ElasticNet | 67 | -0.626 | 1.763 | 683.609 | 289.609 | 568.743 | 263.225 | 0.092 | 0.041 | 0.447 | 0.329 | 0.361 | 0.214 |
| F2 | ElasticNet | 67 | -0.668 | 1.287 | 722.237 | 297.743 | 600.393 | 259.056 | 0.097 | 0.041 | 0.252 | 0.394 | 0.263 | 0.174 |
| F0 | Environment random-effect LMM | 67 | -0.954 | 1.67 | 784.88 | 320.505 | 660.195 | 300.17 | 0.105 | 0.041 |  |  | 0.195 | 0.137 |

**Supplementary Table 9.** Sample-reduction robustness results

| Training fraction | Feature set | Model | Repeats | Mean training records | Mean R² | SD of R² | Mean RMSE | SD of RMSE | Mean MAE | SD of MAE | Mean normalized RMSE | SD of normalized RMSE | Mean Spearman correlation | SD of Spearman correlation | Mean top-20% recall | SD of top-20% recall | Mean runtime (s) | SD of runtime (s) |
| --- | --- | --- | --- | --- | --- | --- | --- | --- | --- | --- | --- | --- | --- | --- | --- | --- | --- | --- |
| 0.2 | F3 | ElasticNet | 30 | 511 | 0.358 | 0.011 | 686.916 | 6.123 | 527.299 | 5.663 | 0.092 | 0.001 | 0.559 | 0.007 | 0.518 | 0.02 | 0.023 | 0.003 |
| 0.4 | F3 | ElasticNet | 30 | 1025 | 0.385 | 0.012 | 671.933 | 6.7 | 516.823 | 5.76 | 0.09 | 0.001 | 0.583 | 0.01 | 0.514 | 0.015 | 0.041 | 0.004 |
| 0.6 | F3 | ElasticNet | 30 | 1538 | 0.398 | 0.009 | 665.13 | 4.807 | 512.203 | 3.627 | 0.089 | 0.001 | 0.593 | 0.007 | 0.517 | 0.013 | 0.057 | 0.006 |
| 0.8 | F3 | ElasticNet | 30 | 2052 | 0.405 | 0.004 | 660.825 | 2.271 | 509.548 | 2.205 | 0.088 | 0 | 0.598 | 0.004 | 0.52 | 0.011 | 0.092 | 0.01 |
| 1 | F3 | ElasticNet | 1 | 2563 | 0.41 | 0 | 658.511 | 0 | 508.08 | 0 | 0.088 | 0 | 0.601 | 0 | 0.527 | 0 | 0.12 | 0 |
| 0.2 | F3 | LightGBM | 30 | 511 | 0.526 | 0.035 | 589.431 | 21.897 | 435.507 | 14.667 | 0.079 | 0.003 | 0.687 | 0.025 | 0.596 | 0.03 | 0.143 | 0.004 |
| 0.4 | F3 | LightGBM | 30 | 1025 | 0.635 | 0.027 | 517.667 | 19.219 | 380.951 | 11.983 | 0.069 | 0.003 | 0.76 | 0.017 | 0.649 | 0.024 | 0.237 | 0.037 |
| 0.6 | F3 | LightGBM | 30 | 1538 | 0.693 | 0.017 | 475.032 | 13.399 | 350.164 | 7.492 | 0.063 | 0.002 | 0.797 | 0.011 | 0.677 | 0.019 | 0.293 | 0.043 |
| 0.8 | F3 | LightGBM | 30 | 2052 | 0.73 | 0.012 | 445.391 | 9.998 | 331.799 | 5.535 | 0.059 | 0.001 | 0.817 | 0.007 | 0.69 | 0.018 | 0.492 | 0.074 |
| 1 | F3 | LightGBM | 1 | 2563 | 0.755 | 0 | 424.083 | 0 | 317.746 | 0 | 0.057 | 0 | 0.832 | 0 | 0.682 | 0 | 0.384 | 0 |
| 0.2 | F3 | Random forest | 30 | 511 | 0.542 | 0.022 | 579.985 | 14.079 | 436.57 | 10.007 | 0.077 | 0.002 | 0.701 | 0.021 | 0.604 | 0.027 | 0.325 | 0.028 |
| 0.4 | F3 | Random forest | 30 | 1025 | 0.632 | 0.018 | 520.045 | 12.661 | 386.05 | 8.278 | 0.069 | 0.002 | 0.766 | 0.013 | 0.648 | 0.024 | 0.637 | 0.086 |
| 0.6 | F3 | Random forest | 30 | 1538 | 0.679 | 0.014 | 485.607 | 10.486 | 356.728 | 5.969 | 0.065 | 0.001 | 0.797 | 0.009 | 0.679 | 0.015 | 1.109 | 0.145 |
| 0.8 | F3 | Random forest | 30 | 2052 | 0.719 | 0.009 | 454.631 | 7.61 | 332.233 | 3.844 | 0.061 | 0.001 | 0.82 | 0.005 | 0.697 | 0.012 | 2.29 | 0.248 |
| 1 | F3 | Random forest | 1 | 2563 | 0.752 | 0 | 426.958 | 0 | 312.511 | 0 | 0.057 | 0 | 0.838 | 0 | 0.729 | 0 | 2.336 | 0 |
| 0.2 | F3 | XGBoost | 30 | 511 | 0.546 | 0.033 | 576.967 | 21.087 | 429.341 | 14.798 | 0.077 | 0.003 | 0.7 | 0.025 | 0.605 | 0.03 | 0.191 | 0.008 |
| 0.4 | F3 | XGBoost | 30 | 1025 | 0.638 | 0.022 | 515.261 | 15.937 | 384.935 | 9.782 | 0.069 | 0.002 | 0.76 | 0.014 | 0.638 | 0.019 | 0.257 | 0.031 |
| 0.6 | F3 | XGBoost | 30 | 1538 | 0.683 | 0.016 | 482.705 | 12.307 | 361.856 | 6.708 | 0.064 | 0.002 | 0.788 | 0.009 | 0.648 | 0.017 | 0.313 | 0.048 |
| 0.8 | F3 | XGBoost | 30 | 2052 | 0.714 | 0.01 | 458.574 | 8.268 | 347.734 | 4.851 | 0.061 | 0.001 | 0.804 | 0.006 | 0.661 | 0.016 | 0.553 | 0.066 |
| 1 | F3 | XGBoost | 1 | 2563 | 0.738 | 0 | 438.909 | 0 | 338.631 | 0 | 0.059 | 0 | 0.817 | 0 | 0.651 | 0 | 0.417 | 0 |
| 0.2 | F4 | ElasticNet | 30 | 511 | 0.574 | 0.008 | 559.251 | 5.509 | 434.404 | 5.604 | 0.075 | 0.001 | 0.723 | 0.008 | 0.606 | 0.019 | 0.041 | 0.004 |
| 0.4 | F4 | ElasticNet | 30 | 1025 | 0.593 | 0.007 | 546.988 | 4.932 | 424.401 | 4.51 | 0.073 | 0.001 | 0.733 | 0.006 | 0.603 | 0.016 | 0.096 | 0.009 |
| 0.6 | F4 | ElasticNet | 30 | 1538 | 0.6 | 0.004 | 541.866 | 2.767 | 420.036 | 2.293 | 0.072 | 0 | 0.739 | 0.004 | 0.606 | 0.012 | 0.126 | 0.016 |
| 0.8 | F4 | ElasticNet | 30 | 2052 | 0.604 | 0.003 | 539.044 | 1.805 | 417.593 | 1.795 | 0.072 | 0 | 0.741 | 0.002 | 0.606 | 0.008 | 0.212 | 0.035 |
| 1 | F4 | ElasticNet | 1 | 2563 | 0.608 | 0 | 536.796 | 0 | 415.526 | 0 | 0.072 | 0 | 0.744 | 0 | 0.605 | 0 | 0.284 | 0 |
| 0.2 | F4 | LightGBM | 30 | 511 | 0.65 | 0.015 | 507.024 | 11.166 | 376.062 | 8.001 | 0.068 | 0.001 | 0.768 | 0.01 | 0.67 | 0.023 | 0.173 | 0.007 |
| 0.4 | F4 | LightGBM | 30 | 1025 | 0.734 | 0.014 | 442.196 | 11.518 | 321.733 | 7.932 | 0.059 | 0.002 | 0.825 | 0.009 | 0.732 | 0.021 | 0.299 | 0.039 |
| 0.6 | F4 | LightGBM | 30 | 1538 | 0.774 | 0.014 | 407.006 | 12.956 | 295.234 | 7.455 | 0.054 | 0.002 | 0.85 | 0.009 | 0.757 | 0.02 | 0.377 | 0.062 |
| 0.8 | F4 | LightGBM | 30 | 2052 | 0.809 | 0.01 | 374.428 | 9.633 | 274.084 | 5.646 | 0.05 | 0.001 | 0.868 | 0.007 | 0.776 | 0.013 | 0.677 | 0.078 |
| 1 | F4 | LightGBM | 1 | 2563 | 0.833 | 0 | 349.914 | 0 | 259.919 | 0 | 0.047 | 0 | 0.882 | 0 | 0.806 | 0 | 0.331 | 0 |
| 0.2 | F4 | Random forest | 30 | 511 | 0.598 | 0.014 | 543.099 | 9.474 | 416.322 | 6.422 | 0.072 | 0.001 | 0.748 | 0.013 | 0.648 | 0.024 | 0.363 | 0.008 |
| 0.4 | F4 | Random forest | 30 | 1025 | 0.682 | 0.011 | 482.828 | 8.679 | 365.426 | 5.9 | 0.064 | 0.001 | 0.806 | 0.009 | 0.682 | 0.021 | 0.779 | 0.065 |
| 0.6 | F4 | Random forest | 30 | 1538 | 0.732 | 0.01 | 443.909 | 8.646 | 331.563 | 4.852 | 0.059 | 0.001 | 0.835 | 0.007 | 0.708 | 0.017 | 1.346 | 0.175 |
| 0.8 | F4 | Random forest | 30 | 2052 | 0.772 | 0.008 | 408.813 | 6.903 | 302.946 | 3.477 | 0.055 | 0.001 | 0.859 | 0.005 | 0.729 | 0.016 | 2.992 | 0.268 |
| 1 | F4 | Random forest | 1 | 2563 | 0.809 | 0 | 374.626 | 0 | 278.871 | 0 | 0.05 | 0 | 0.88 | 0 | 0.76 | 0 | 1.878 | 0 |
| 0.2 | F4 | XGBoost | 30 | 511 | 0.658 | 0.016 | 500.929 | 12.058 | 374.097 | 8.423 | 0.067 | 0.002 | 0.775 | 0.013 | 0.683 | 0.019 | 0.255 | 0.008 |
| 0.4 | F4 | XGBoost | 30 | 1025 | 0.733 | 0.013 | 442.948 | 10.765 | 330.275 | 7.398 | 0.059 | 0.001 | 0.825 | 0.008 | 0.729 | 0.017 | 0.359 | 0.045 |
| 0.6 | F4 | XGBoost | 30 | 1538 | 0.766 | 0.013 | 414.082 | 11.484 | 308.261 | 6.077 | 0.055 | 0.002 | 0.846 | 0.007 | 0.759 | 0.019 | 0.443 | 0.062 |
| 0.8 | F4 | XGBoost | 30 | 2052 | 0.794 | 0.009 | 388.964 | 8.782 | 293.113 | 4.954 | 0.052 | 0.001 | 0.858 | 0.005 | 0.775 | 0.012 | 0.751 | 0.077 |
| 1 | F4 | XGBoost | 1 | 2563 | 0.811 | 0 | 372.752 | 0 | 284.961 | 0 | 0.05 | 0 | 0.87 | 0 | 0.783 | 0 | 0.364 | 0 |
| 0.2 | F5 | ElasticNet | 30 | 511 | 0.323 | 0.012 | 705.203 | 6.079 | 539.873 | 5.781 | 0.094 | 0.001 | 0.533 | 0.008 | 0.515 | 0.025 | 0.014 | 0.002 |
| 0.4 | F5 | ElasticNet | 30 | 1025 | 0.352 | 0.012 | 689.703 | 6.527 | 529.433 | 6.141 | 0.092 | 0.001 | 0.56 | 0.01 | 0.504 | 0.017 | 0.036 | 0.005 |
| 0.6 | F5 | ElasticNet | 30 | 1538 | 0.364 | 0.01 | 683.511 | 5.117 | 524.541 | 4.486 | 0.091 | 0.001 | 0.57 | 0.008 | 0.504 | 0.014 | 0.047 | 0.006 |
| 0.8 | F5 | ElasticNet | 30 | 2052 | 0.372 | 0.005 | 679.253 | 2.773 | 521.937 | 2.56 | 0.091 | 0 | 0.577 | 0.005 | 0.501 | 0.013 | 0.081 | 0.008 |
| 1 | F5 | ElasticNet | 1 | 2563 | 0.375 | 0 | 677.343 | 0 | 520.969 | 0 | 0.09 | 0 | 0.578 | 0 | 0.504 | 0 | 0.062 | 0 |
| 0.2 | F5 | LightGBM | 30 | 511 | 0.47 | 0.042 | 623.452 | 24.69 | 462.853 | 16.728 | 0.083 | 0.003 | 0.661 | 0.027 | 0.572 | 0.029 | 0.107 | 0.008 |
| 0.4 | F5 | LightGBM | 30 | 1025 | 0.596 | 0.029 | 544.423 | 19.068 | 400.125 | 13.718 | 0.073 | 0.003 | 0.742 | 0.017 | 0.637 | 0.021 | 0.159 | 0.016 |
| 0.6 | F5 | LightGBM | 30 | 1538 | 0.657 | 0.019 | 502.035 | 13.695 | 367.494 | 8.394 | 0.067 | 0.002 | 0.783 | 0.011 | 0.658 | 0.018 | 0.203 | 0.028 |
| 0.8 | F5 | LightGBM | 30 | 2052 | 0.692 | 0.011 | 475.758 | 8.868 | 348.861 | 5.53 | 0.063 | 0.001 | 0.804 | 0.006 | 0.673 | 0.015 | 0.328 | 0.039 |
| 1 | F5 | LightGBM | 1 | 2563 | 0.709 | 0 | 461.936 | 0 | 338.955 | 0 | 0.062 | 0 | 0.813 | 0 | 0.674 | 0 | 0.206 | 0 |
| 0.2 | F5 | Random forest | 30 | 511 | 0.502 | 0.024 | 604.361 | 14.587 | 454.623 | 10.259 | 0.081 | 0.002 | 0.685 | 0.023 | 0.592 | 0.031 | 0.276 | 0.007 |
| 0.4 | F5 | Random forest | 30 | 1025 | 0.602 | 0.017 | 540.356 | 11.716 | 401.605 | 7.888 | 0.072 | 0.002 | 0.756 | 0.012 | 0.639 | 0.02 | 0.591 | 0.05 |
| 0.6 | F5 | Random forest | 30 | 1538 | 0.65 | 0.016 | 507.151 | 11.558 | 371.56 | 7.075 | 0.068 | 0.002 | 0.787 | 0.009 | 0.667 | 0.016 | 0.949 | 0.121 |
| 0.8 | F5 | Random forest | 30 | 2052 | 0.687 | 0.012 | 479.194 | 9.164 | 347.238 | 5.102 | 0.064 | 0.001 | 0.81 | 0.006 | 0.679 | 0.018 | 1.888 | 0.161 |
| 1 | F5 | Random forest | 1 | 2563 | 0.717 | 0 | 456.018 | 0 | 328.924 | 0 | 0.061 | 0 | 0.827 | 0 | 0.705 | 0 | 1.069 | 0 |
| 0.2 | F5 | XGBoost | 30 | 511 | 0.493 | 0.038 | 609.52 | 22.862 | 450.913 | 14.543 | 0.081 | 0.003 | 0.676 | 0.027 | 0.585 | 0.035 | 0.131 | 0.018 |
| 0.4 | F5 | XGBoost | 30 | 1025 | 0.601 | 0.026 | 540.788 | 17.734 | 401.56 | 12.292 | 0.072 | 0.002 | 0.746 | 0.017 | 0.619 | 0.02 | 0.161 | 0.019 |
| 0.6 | F5 | XGBoost | 30 | 1538 | 0.652 | 0.018 | 505.61 | 13.339 | 376.807 | 7.426 | 0.067 | 0.002 | 0.779 | 0.011 | 0.628 | 0.019 | 0.205 | 0.029 |
| 0.8 | F5 | XGBoost | 30 | 2052 | 0.678 | 0.012 | 486.601 | 9.193 | 364.502 | 5.634 | 0.065 | 0.001 | 0.793 | 0.007 | 0.646 | 0.016 | 0.341 | 0.042 |
| 1 | F5 | XGBoost | 1 | 2563 | 0.699 | 0 | 470.536 | 0 | 354.387 | 0 | 0.063 | 0 | 0.8 | 0 | 0.643 | 0 | 0.178 | 0 |

**Supplementary Table 10.** Extreme-environment thresholds

| Risk type | Label | Variable | Direction | Quantile | Threshold | Flagged environments |
| --- | --- | --- | --- | --- | --- | --- |
| high temperature | High temperature | tmax mean heading maturity | high | 0.9 | 32.79 | 7 |
| heavy rainfall | Heavy rainfall | tp sum heading maturity | high | 0.9 | 361.651 | 7 |
| high humidity | High humidity | RH mean heading maturity | high | 0.9 | 83.443 | 7 |
| low radiation | Low radiation | ssrd sum heading maturity | low | 0.1 | 5358.126 | 7 |
| low sunshine | Low sunshine | ssd sum heading maturity | low | 0.1 | 143.408 | 7 |

**Supplementary Table 11.** Extreme-environment classification

| Env | Testsite | Year | Records | Varieties | Yield mean | Yield sd | Tmax mean heading maturity | Tp sum heading maturity | Rh mean heading maturity | Ssrd sum heading maturity | Ssd sum heading maturity | High temperature flag | High temperature severity | Heavy rainfall flag | Heavy rainfall severity | High humidity flag | High humidity severity | Low radiation flag | Low radiation severity | Low sunshine flag | Low sunshine severity | Extreme risk count | Extreme environment | Climate class | Extreme severity score | Extreme risk types |
| --- | --- | --- | --- | --- | --- | --- | --- | --- | --- | --- | --- | --- | --- | --- | --- | --- | --- | --- | --- | --- | --- | --- | --- | --- | --- | --- |
| GaoZhou 2016 | GaoZhou | 2016 | 38 | 37 | 7079.337 | 650.382 | 32.773 | 125.846 | 80.608 | 6572.368 | 206.892 | FALSE | 0.896 | FALSE | 0.119 | FALSE | 0.672 | FALSE | 0.224 | FALSE | 0.119 | 0 | FALSE | Normal | 0.896 |  |
| GaoZhou 2017 | GaoZhou | 2017 | 33 | 32 | 7018.682 | 667.776 | 31.735 | 276.694 | 84.138 | 5981.082 | 178.87 | FALSE | 0.627 | FALSE | 0.612 | TRUE | 0.925 | FALSE | 0.493 | FALSE | 0.418 | 1 | TRUE | Extreme | 0.925 | high humidity |
| GaoZhou 2018 | GaoZhou | 2018 | 37 | 36 | 7767.374 | 513.098 | 31.533 | 209.615 | 80.496 | 6088.72 | 186.543 | FALSE | 0.478 | FALSE | 0.418 | FALSE | 0.657 | FALSE | 0.403 | FALSE | 0.328 | 0 | FALSE | Normal | 0.657 |  |
| GaoZhou 2019 | GaoZhou | 2019 | 12 | 12 | 7076.8 | 531.348 | 31.917 | 22.075 | 74.243 | 6808.885 | 246.867 | FALSE | 0.672 | FALSE | 0.015 | FALSE | 0.075 | FALSE | 0.179 | FALSE | 0.015 | 0 | FALSE | Normal | 0.672 |  |
| GaoZhou 2020 | GaoZhou | 2020 | 31 | 30 | 7213.13 | 687.146 | 32.081 | 177.595 | 81.401 | 6149.023 | 193.61 | FALSE | 0.731 | FALSE | 0.269 | FALSE | 0.776 | FALSE | 0.358 | FALSE | 0.284 | 0 | FALSE | Normal | 0.776 |  |
| GaoZhou 2021 | GaoZhou | 2021 | 36 | 35 | 7273.346 | 546.151 | 32.003 | 160.257 | 79.489 | 6420.98 | 199.949 | FALSE | 0.716 | FALSE | 0.239 | FALSE | 0.537 | FALSE | 0.254 | FALSE | 0.194 | 0 | FALSE | Normal | 0.716 |  |
| GaoZhou 2022 | GaoZhou | 2022 | 34 | 33 | 7587.168 | 447.221 | 31.299 | 356.614 | 81.085 | 6012.604 | 195.502 | FALSE | 0.313 | FALSE | 0.881 | FALSE | 0.761 | FALSE | 0.478 | FALSE | 0.239 | 0 | FALSE | Normal | 0.881 |  |
| GuangZhou 2016 | GuangZhou | 2016 | 76 | 37 | 7152.6 | 333.362 | 31.766 | 244.749 | 80.832 | 5916.246 | 178.753 | FALSE | 0.642 | FALSE | 0.567 | FALSE | 0.701 | FALSE | 0.522 | FALSE | 0.433 | 0 | FALSE | Normal | 0.701 |  |
| GuangZhou 2017 | GuangZhou | 2017 | 66 | 32 | 7448.786 | 354.737 | 30.698 | 275.897 | 78.593 | 5670.244 | 160.199 | FALSE | 0.164 | FALSE | 0.597 | FALSE | 0.403 | FALSE | 0.731 | FALSE | 0.657 | 0 | FALSE | Normal | 0.731 |  |
| GuangZhou 2018 | GuangZhou | 2018 | 74 | 36 | 7579.694 | 1146.597 | 30.407 | 362.414 | 80.276 | 5253.561 | 144.033 | FALSE | 0.09 | TRUE | 0.91 | FALSE | 0.627 | TRUE | 0.925 | FALSE | 0.851 | 2 | TRUE | Extreme | 0.925 | heavy rainfall;low radiation |
| GuangZhou 2019 | GuangZhou | 2019 | 50 | 24 | 7587.144 | 424.542 | 33.062 | 378.666 | 82.627 | 5885.299 | 143.702 | TRUE | 0.955 | TRUE | 0.925 | FALSE | 0.866 | FALSE | 0.552 | FALSE | 0.866 | 2 | TRUE | Extreme | 0.955 | high temperature;heavy rainfall |
| GuangZhou 2020 | GuangZhou | 2020 | 62 | 30 | 7888.101 | 833.973 | 32.125 | 105.483 | 75.13 | 6057.932 | 185.286 | FALSE | 0.746 | FALSE | 0.09 | FALSE | 0.104 | FALSE | 0.448 | FALSE | 0.358 | 0 | FALSE | Normal | 0.746 |  |
| GuangZhou 2021 | GuangZhou | 2021 | 72 | 35 | 6555.358 | 497.837 | 31.53 | 225.193 | 76.459 | 5727.732 | 156.036 | FALSE | 0.448 | FALSE | 0.463 | FALSE | 0.134 | FALSE | 0.657 | FALSE | 0.716 | 0 | FALSE | Normal | 0.716 |  |
| GuangZhou 2022 | GuangZhou | 2022 | 67 | 33 | 6339.304 | 511.223 | 31.009 | 309.727 | 77.464 | 5577.599 | 156.141 | FALSE | 0.239 | FALSE | 0.806 | FALSE | 0.224 | FALSE | 0.806 | FALSE | 0.701 | 0 | FALSE | Normal | 0.806 |  |
| HaiKou 2016 | HaiKou | 2016 | 76 | 37 | 6776.317 | 1070.041 | 32.554 | 279.337 | 83.435 | 6775.286 | 200.841 | FALSE | 0.836 | FALSE | 0.672 | FALSE | 0.896 | FALSE | 0.194 | FALSE | 0.179 | 0 | FALSE | Normal | 0.896 |  |
| HaiKou 2019 | HaiKou | 2019 | 74 | 38 | 6911.372 | 939.733 | 32.183 | 317.309 | 82.117 | 7206.394 | 203.038 | FALSE | 0.761 | FALSE | 0.851 | FALSE | 0.821 | FALSE | 0.075 | FALSE | 0.149 | 0 | FALSE | Normal | 0.851 |  |
| HaiKou 2021 | HaiKou | 2021 | 72 | 35 | 7192.258 | 1200.142 | 32.923 | 297.103 | 78.294 | 7000.502 | 204.286 | TRUE | 0.925 | FALSE | 0.746 | FALSE | 0.328 | FALSE | 0.149 | FALSE | 0.134 | 1 | TRUE | Extreme | 0.925 | high temperature |
| HaiKou 2022 | HaiKou | 2022 | 46 | 22 | 6799.187 | 731.298 | 31.482 | 288.134 | 85.435 | 7761.79 | 168.273 | FALSE | 0.403 | FALSE | 0.701 | TRUE | 0.97 | FALSE | 0.03 | FALSE | 0.567 | 1 | TRUE | Extreme | 0.97 | high humidity |
| HuiZhou 2016 | HuiZhou | 2016 | 38 | 37 | 7415.791 | 648.438 | 31.044 | 306.64 | 81.698 | 5740.942 | 181.55 | FALSE | 0.254 | FALSE | 0.776 | FALSE | 0.806 | FALSE | 0.642 | FALSE | 0.403 | 0 | FALSE | Normal | 0.806 |  |
| HuiZhou 2017 | HuiZhou | 2017 | 33 | 32 | 7154.091 | 389.772 | 30.322 | 416.769 | 78.78 | 6012.764 | 181.625 | FALSE | 0.075 | TRUE | 0.94 | FALSE | 0.418 | FALSE | 0.463 | FALSE | 0.388 | 1 | TRUE | Extreme | 0.94 | heavy rainfall |
| HuiZhou 2018 | HuiZhou | 2018 | 37 | 36 | 8269.931 | 636.031 | 30.114 | 361.141 | 79.226 | 5607.116 | 157.581 | FALSE | 0.045 | FALSE | 0.896 | FALSE | 0.507 | FALSE | 0.791 | FALSE | 0.687 | 0 | FALSE | Normal | 0.896 |  |
| HuiZhou 2019 | HuiZhou | 2019 | 25 | 24 | 6396.528 | 456.787 | 32.41 | 290.495 | 83.456 | 6302.531 | 151.218 | FALSE | 0.791 | FALSE | 0.731 | TRUE | 0.91 | FALSE | 0.284 | FALSE | 0.776 | 1 | TRUE | Extreme | 0.91 | high humidity |
| HuiZhou 2020 | HuiZhou | 2020 | 31 | 30 | 7346.081 | 955.829 | 31.943 | 122.964 | 72.517 | 6707.392 | 209.82 | FALSE | 0.687 | FALSE | 0.104 | FALSE | 0.06 | FALSE | 0.209 | FALSE | 0.075 | 0 | FALSE | Normal | 0.687 |  |
| HuiZhou 2021 | HuiZhou | 2021 | 36 | 35 | 7008.917 | 736.933 | 30.683 | 253.284 | 77.718 | 5464.13 | 144.984 | FALSE | 0.149 | FALSE | 0.582 | FALSE | 0.269 | FALSE | 0.836 | FALSE | 0.836 | 0 | FALSE | Normal | 0.836 |  |
| HuiZhou 2022 | HuiZhou | 2022 | 34 | 33 | 8009.246 | 776.107 | 29.949 | 308.871 | 77.672 | 5411.142 | 149.041 | FALSE | 0.015 | FALSE | 0.791 | FALSE | 0.239 | FALSE | 0.866 | FALSE | 0.791 | 0 | FALSE | Normal | 0.866 |  |
| NanNing 2016 | NanNing | 2016 | 76 | 37 | 7901.211 | 283.816 | 32.738 | 208.232 | 78.436 | 6549.081 | 197.05 | FALSE | 0.881 | FALSE | 0.403 | FALSE | 0.373 | FALSE | 0.239 | FALSE | 0.224 | 0 | FALSE | Normal | 0.881 |  |
| NanNing 2017 | NanNing | 2017 | 44 | 21 | 7900.786 | 769.648 | 31.635 | 240.752 | 87.962 | 5801.084 | 119.877 | FALSE | 0.537 | FALSE | 0.507 | TRUE | 1 | FALSE | 0.582 | TRUE | 0.97 | 2 | TRUE | Extreme | 1 | high humidity;low sunshine |
| NanNing 2018 | NanNing | 2018 | 74 | 36 | 8078.209 | 575.044 | 30.606 | 187.4 | 79.875 | 6177.11 | 169.682 | FALSE | 0.119 | FALSE | 0.299 | FALSE | 0.567 | FALSE | 0.343 | FALSE | 0.537 | 0 | FALSE | Normal | 0.567 |  |
| NanNing 2019 | NanNing | 2019 | 50 | 24 | 7822.098 | 365.352 | 33.037 | 192.255 | 85.398 | 5680.037 | 121.31 | TRUE | 0.94 | FALSE | 0.313 | TRUE | 0.955 | FALSE | 0.716 | TRUE | 0.955 | 3 | TRUE | Extreme | 0.955 | high temperature;high humidity;low sunshine |
| NanNing 2020 | NanNing | 2020 | 62 | 30 | 7326.949 | 802.311 | 31.391 | 143.662 | 81.694 | 6095.508 | 143.135 | FALSE | 0.358 | FALSE | 0.179 | FALSE | 0.791 | FALSE | 0.373 | TRUE | 0.896 | 1 | TRUE | Extreme | 0.896 | low sunshine |
| NanNing 2021 | NanNing | 2021 | 72 | 35 | 7381.229 | 501.007 | 31.618 | 157.249 | 79.449 | 5899.946 | 140.061 | FALSE | 0.522 | FALSE | 0.224 | FALSE | 0.522 | FALSE | 0.537 | TRUE | 0.91 | 1 | TRUE | Extreme | 0.91 | low sunshine |
| NanNing 2022 | NanNing | 2022 | 46 | 22 | 8033.23 | 494.112 | 32.427 | 204.3 | 85.96 | 4666.371 | 86.2 | FALSE | 0.806 | FALSE | 0.373 | TRUE | 0.985 | TRUE | 0.985 | TRUE | 0.985 | 3 | TRUE | Extreme | 0.985 | high humidity;low radiation;low sunshine |
| QinZhou 2016 | QinZhou | 2016 | 76 | 37 | 7713.434 | 339.808 | 31.538 | 576.483 | 80.088 | 7093.559 | 236.671 | FALSE | 0.493 | TRUE | 1 | FALSE | 0.612 | FALSE | 0.09 | FALSE | 0.03 | 1 | TRUE | Extreme | 1 | heavy rainfall |
| QinZhou 2017 | QinZhou | 2017 | 66 | 32 | 7625.009 | 260.304 | 30.051 | 420.594 | 82.388 | 5253.749 | 155.201 | FALSE | 0.03 | TRUE | 0.955 | FALSE | 0.851 | TRUE | 0.91 | FALSE | 0.731 | 2 | TRUE | Extreme | 0.955 | heavy rainfall;low radiation |
| QinZhou 2018 | QinZhou | 2018 | 74 | 36 | 7404.634 | 216.181 | 31.103 | 278.866 | 77.708 | 6258.854 | 208.622 | FALSE | 0.269 | FALSE | 0.657 | FALSE | 0.254 | FALSE | 0.313 | FALSE | 0.09 | 0 | FALSE | Normal | 0.657 |  |
| QinZhou 2019 | QinZhou | 2019 | 74 | 38 | 7348.703 | 629.024 | 31.951 | 197.112 | 80.355 | 6203.044 | 194.636 | FALSE | 0.701 | FALSE | 0.343 | FALSE | 0.642 | FALSE | 0.328 | FALSE | 0.269 | 0 | FALSE | Normal | 0.701 |  |
| QinZhou 2020 | QinZhou | 2020 | 62 | 30 | 7009.933 | 527.277 | 31.512 | 278.506 | 76.564 | 6073.125 | 202.332 | FALSE | 0.433 | FALSE | 0.642 | FALSE | 0.164 | FALSE | 0.418 | FALSE | 0.164 | 0 | FALSE | Normal | 0.642 |  |
| QinZhou 2021 | QinZhou | 2021 | 72 | 35 | 7289.925 | 588.901 | 31.452 | 244.015 | 78.55 | 7029.123 | 231.844 | FALSE | 0.388 | FALSE | 0.552 | FALSE | 0.388 | FALSE | 0.119 | FALSE | 0.045 | 0 | FALSE | Normal | 0.552 |  |
| QinZhou 2022 | QinZhou | 2022 | 67 | 33 | 7522.894 | 292.464 | 31.486 | 313.314 | 80.994 | 5882.08 | 186.102 | FALSE | 0.418 | FALSE | 0.821 | FALSE | 0.716 | FALSE | 0.567 | FALSE | 0.343 | 0 | FALSE | Normal | 0.821 |  |
| QingYuan 2016 | QingYuan | 2016 | 38 | 37 | 7378.942 | 463.686 | 31.72 | 224.316 | 81.042 | 6089.55 | 183.201 | FALSE | 0.612 | FALSE | 0.448 | FALSE | 0.746 | FALSE | 0.388 | FALSE | 0.373 | 0 | FALSE | Normal | 0.746 |  |
| QingYuan 2017 | QingYuan | 2017 | 33 | 32 | 7004.164 | 544.771 | 30.652 | 349.428 | 79.967 | 5707.193 | 174.385 | FALSE | 0.134 | FALSE | 0.866 | FALSE | 0.582 | FALSE | 0.701 | FALSE | 0.463 | 0 | FALSE | Normal | 0.866 |  |
| QingYuan 2018 | QingYuan | 2018 | 37 | 36 | 6783.467 | 541.03 | 30.304 | 315.572 | 79.522 | 5641.105 | 160.36 | FALSE | 0.06 | FALSE | 0.836 | FALSE | 0.552 | FALSE | 0.761 | FALSE | 0.642 | 0 | FALSE | Normal | 0.836 |  |
| QingYuan 2019 | QingYuan | 2019 | 25 | 24 | 6869.622 | 343.91 | 32.816 | 301.531 | 85.076 | 5146.739 | 133.476 | TRUE | 0.91 | FALSE | 0.761 | TRUE | 0.94 | TRUE | 0.955 | TRUE | 0.94 | 4 | TRUE | Extreme | 0.955 | high temperature;high humidity;low radiation;low sunshine |
| QingYuan 2020 | QingYuan | 2020 | 31 | 30 | 7158.634 | 515.041 | 31.665 | 102.846 | 79.038 | 5408.881 | 172.201 | FALSE | 0.552 | FALSE | 0.06 | FALSE | 0.463 | FALSE | 0.881 | FALSE | 0.478 | 0 | FALSE | Normal | 0.881 |  |
| QingYuan 2021 | QingYuan | 2021 | 36 | 35 | 7031.646 | 803.296 | 31.385 | 289.055 | 78.95 | 5440.161 | 145.003 | FALSE | 0.343 | FALSE | 0.716 | FALSE | 0.448 | FALSE | 0.851 | FALSE | 0.821 | 0 | FALSE | Normal | 0.851 |  |
| QingYuan 2022 | QingYuan | 2022 | 34 | 33 | 7403.519 | 789.342 | 30.884 | 534.476 | 75.609 | 5668.556 | 162.539 | FALSE | 0.194 | TRUE | 0.985 | FALSE | 0.119 | FALSE | 0.746 | FALSE | 0.597 | 1 | TRUE | Extreme | 0.985 | heavy rainfall |
| YuLin 2016 | YuLin | 2016 | 38 | 37 | 7878.805 | 430.996 | 32.443 | 155.773 | 77.961 | 6297.801 | 191.15 | FALSE | 0.821 | FALSE | 0.209 | FALSE | 0.299 | FALSE | 0.299 | FALSE | 0.299 | 0 | FALSE | Normal | 0.821 |  |
| YuLin 2017 | YuLin | 2017 | 33 | 32 | 7271.9 | 815.784 | 30.996 | 276.754 | 83.429 | 5156.59 | 151.721 | FALSE | 0.224 | FALSE | 0.627 | FALSE | 0.881 | TRUE | 0.94 | FALSE | 0.761 | 1 | TRUE | Extreme | 0.94 | low radiation |
| YuLin 2018 | YuLin | 2018 | 37 | 36 | 7408.646 | 747.298 | 31.14 | 144.317 | 81.021 | 5709.443 | 161.202 | FALSE | 0.299 | FALSE | 0.194 | FALSE | 0.731 | FALSE | 0.687 | FALSE | 0.627 | 0 | FALSE | Normal | 0.731 |  |
| YuLin 2019 | YuLin | 2019 | 37 | 36 | 7751.534 | 520.375 | 31.696 | 142.677 | 82.15 | 5281.993 | 153.194 | FALSE | 0.597 | FALSE | 0.164 | FALSE | 0.836 | TRUE | 0.896 | FALSE | 0.746 | 1 | TRUE | Extreme | 0.896 | low radiation |
| YuLin 2020 | YuLin | 2020 | 31 | 30 | 8095.602 | 607.301 | 31.608 | 138.031 | 77.046 | 5957.804 | 168.374 | FALSE | 0.507 | FALSE | 0.149 | FALSE | 0.209 | FALSE | 0.507 | FALSE | 0.552 | 0 | FALSE | Normal | 0.552 |  |
| YuLin 2021 | YuLin | 2021 | 36 | 35 | 7585.525 | 580.167 | 31.348 | 127.281 | 78.335 | 5726.814 | 159.471 | FALSE | 0.328 | FALSE | 0.134 | FALSE | 0.343 | FALSE | 0.672 | FALSE | 0.672 | 0 | FALSE | Normal | 0.672 |  |
| YuLin 2022 | YuLin | 2022 | 34 | 33 | 7382.951 | 580.487 | 30.552 | 243.07 | 78.158 | 5055.417 | 148.793 | FALSE | 0.104 | FALSE | 0.537 | FALSE | 0.313 | TRUE | 0.97 | FALSE | 0.806 | 1 | TRUE | Extreme | 0.97 | low radiation |
| ZhangZhou 2016 | ZhangZhou | 2016 | 50 | 24 | 8722.794 | 565.789 | 34.037 | 242.785 | 80.697 | 7019.758 | 190.21 | TRUE | 1 | FALSE | 0.522 | FALSE | 0.687 | FALSE | 0.134 | FALSE | 0.313 | 1 | TRUE | Extreme | 1 | high temperature |
| ZhangZhou 2017 | ZhangZhou | 2017 | 65 | 32 | 9083.275 | 808.024 | 31.531 | 205.751 | 80.066 | 6060.99 | 171.955 | FALSE | 0.463 | FALSE | 0.388 | FALSE | 0.597 | FALSE | 0.433 | FALSE | 0.493 | 0 | FALSE | Normal | 0.597 |  |
| ZhangZhou 2018 | ZhangZhou | 2018 | 48 | 23 | 9251.358 | 610.722 | 33.778 | 236.333 | 79.178 | 7388.575 | 194.909 | TRUE | 0.985 | FALSE | 0.493 | FALSE | 0.493 | FALSE | 0.06 | FALSE | 0.254 | 1 | TRUE | Extreme | 0.985 | high temperature |
| ZhangZhou 2019 | ZhangZhou | 2019 | 24 | 14 | 7449.438 | 910.235 | 30.898 | 35.216 | 65.822 | 8041.493 | 299.549 | FALSE | 0.209 | FALSE | 0.03 | FALSE | 0.015 | FALSE | 0 | FALSE | 0 | 0 | FALSE | Normal | 0.209 |  |
| ZhangZhou 2020 | ZhangZhou | 2020 | 62 | 30 | 7267.29 | 1143.01 | 33.712 | 56.6 | 71.56 | 7773.781 | 213.624 | TRUE | 0.97 | FALSE | 0.045 | FALSE | 0.045 | FALSE | 0.015 | FALSE | 0.06 | 1 | TRUE | Extreme | 0.97 | high temperature |
| ZhangZhou 2021 | ZhangZhou | 2021 | 72 | 35 | 8495.054 | 580.423 | 31.872 | 215.81 | 77.79 | 7485.922 | 197.651 | FALSE | 0.657 | FALSE | 0.433 | FALSE | 0.284 | FALSE | 0.045 | FALSE | 0.209 | 0 | FALSE | Normal | 0.657 |  |
| ZhangZhou 2022 | ZhangZhou | 2022 | 67 | 33 | 8157.152 | 588.764 | 31.429 | 202.678 | 79.08 | 6990.837 | 207.584 | FALSE | 0.373 | FALSE | 0.358 | FALSE | 0.478 | FALSE | 0.164 | FALSE | 0.104 | 0 | FALSE | Normal | 0.478 |  |
| ZhaoQing 2016 | ZhaoQing | 2016 | 38 | 37 | 7178.937 | 617.677 | 32.214 | 186.323 | 76.476 | 5772.641 | 170.267 | FALSE | 0.776 | FALSE | 0.284 | FALSE | 0.149 | FALSE | 0.612 | FALSE | 0.522 | 0 | FALSE | Normal | 0.776 |  |
| ZhaoQing 2017 | ZhaoQing | 2017 | 33 | 32 | 7585.482 | 487.119 | 31.132 | 175.157 | 78.382 | 5794.456 | 171.723 | FALSE | 0.284 | FALSE | 0.254 | FALSE | 0.358 | FALSE | 0.597 | FALSE | 0.507 | 0 | FALSE | Normal | 0.597 |  |
| ZhaoQing 2018 | ZhaoQing | 2018 | 24 | 23 | 7252.556 | 647.643 | 32.71 | 492.633 | 78.781 | 7032.185 | 164.025 | FALSE | 0.866 | TRUE | 0.97 | FALSE | 0.433 | FALSE | 0.104 | FALSE | 0.582 | 1 | TRUE | Extreme | 0.97 | heavy rainfall |
| ZhaoQing 2019 | ZhaoQing | 2019 | 37 | 36 | 7620.482 | 812.801 | 32.555 | 196.519 | 76.638 | 6360.775 | 177.403 | FALSE | 0.851 | FALSE | 0.328 | FALSE | 0.179 | FALSE | 0.269 | FALSE | 0.448 | 0 | FALSE | Normal | 0.851 |  |
| ZhaoQing 2020 | ZhaoQing | 2020 | 29 | 29 | 7451.595 | 611.519 | 31.693 | 103.875 | 76.793 | 5756.798 | 161.63 | FALSE | 0.582 | FALSE | 0.075 | FALSE | 0.194 | FALSE | 0.627 | FALSE | 0.612 | 0 | FALSE | Normal | 0.627 |  |
| ZhaoQing 2021 | ZhaoQing | 2021 | 36 | 35 | 7569.854 | 799.637 | 31.681 | 233.07 | 74.473 | 5499.237 | 135.88 | FALSE | 0.567 | FALSE | 0.478 | FALSE | 0.09 | FALSE | 0.821 | TRUE | 0.925 | 1 | TRUE | Extreme | 0.925 | low sunshine |
| ZhaoQing 2022 | ZhaoQing | 2022 | 34 | 33 | 7435.893 | 570.875 | 30.799 | 282.144 | 71.249 | 5624.077 | 143.589 | FALSE | 0.179 | FALSE | 0.687 | FALSE | 0.03 | FALSE | 0.776 | FALSE | 0.881 | 0 | FALSE | Normal | 0.881 |  |

**Supplementary Table 12.** Information-group SHAP contribution

| Feature set | Model | Feature group | Feature group label | Climate class | Mean absolute SHAP value | Total abs shap | Share of total abs shap |
| --- | --- | --- | --- | --- | --- | --- | --- |
| F3 | LightGBM | weather sowing to heading | Weather: sowing to heading | All | 37.428 | 1079268.446 | 0.295 |
| F3 | LightGBM | weather heading to maturity | Weather: heading to maturity | All | 33.507 | 966211.628 | 0.264 |
| F3 | LightGBM | environment management | Environment and management | All | 53.03 | 849534.319 | 0.233 |
| F3 | LightGBM | blup core | Cultivar genetic potential | All | 236.811 | 758743.093 | 0.208 |
| F3 | LightGBM | weather sowing to heading | Weather: sowing to heading | Extreme | 37.579 | 382859.49 | 0.292 |
| F3 | LightGBM | weather heading to maturity | Weather: heading to maturity | Extreme | 33.408 | 340365.631 | 0.26 |
| F3 | LightGBM | environment management | Environment and management | Extreme | 54.946 | 310995.769 | 0.237 |
| F3 | LightGBM | blup core | Cultivar genetic potential | Extreme | 243.864 | 276054.288 | 0.211 |
| F3 | LightGBM | weather sowing to heading | Weather: sowing to heading | Normal | 37.345 | 696408.956 | 0.297 |
| F3 | LightGBM | weather heading to maturity | Weather: heading to maturity | Normal | 33.561 | 625845.997 | 0.267 |
| F3 | LightGBM | environment management | Environment and management | Normal | 51.982 | 538538.551 | 0.23 |
| F3 | LightGBM | blup core | Cultivar genetic potential | Normal | 232.958 | 482688.805 | 0.206 |
| F4 | LightGBM | agronomic trait | Observed agronomic traits | All | 82.107 | 2630700.481 | 0.551 |
| F4 | LightGBM | weather sowing to heading | Weather: sowing to heading | All | 20.864 | 601624.15 | 0.126 |
| F4 | LightGBM | environment management | Environment and management | All | 36.895 | 591059.64 | 0.124 |
| F4 | LightGBM | weather heading to maturity | Weather: heading to maturity | All | 16.883 | 486848.843 | 0.102 |
| F4 | LightGBM | blup core | Cultivar genetic potential | All | 144.931 | 464357.879 | 0.097 |
| F4 | LightGBM | agronomic trait | Observed agronomic traits | Extreme | 83.835 | 949014.986 | 0.553 |
| F4 | LightGBM | environment management | Environment and management | Extreme | 37.961 | 214857.999 | 0.125 |
| F4 | LightGBM | weather sowing to heading | Weather: sowing to heading | Extreme | 20.695 | 210844.3 | 0.123 |
| F4 | LightGBM | weather heading to maturity | Weather: heading to maturity | Extreme | 17.278 | 176029.335 | 0.103 |
| F4 | LightGBM | blup core | Cultivar genetic potential | Extreme | 146.281 | 165589.911 | 0.096 |
| F4 | LightGBM | agronomic trait | Observed agronomic traits | Normal | 81.162 | 1681685.494 | 0.55 |
| F4 | LightGBM | weather sowing to heading | Weather: sowing to heading | Normal | 20.956 | 390779.85 | 0.128 |
| F4 | LightGBM | environment management | Environment and management | Normal | 36.313 | 376201.641 | 0.123 |
| F4 | LightGBM | weather heading to maturity | Weather: heading to maturity | Normal | 16.668 | 310819.508 | 0.102 |
| F4 | LightGBM | blup core | Cultivar genetic potential | Normal | 144.193 | 298767.968 | 0.098 |
| F5 | Random forest | weather sowing to heading | Weather: sowing to heading | All | 45.353 | 1307799.37 | 0.498 |
| F5 | Random forest | environment management | Environment and management | All | 54.164 | 694159.589 | 0.264 |
| F5 | Random forest | blup core | Cultivar genetic potential | All | 156.387 | 501062.358 | 0.191 |
| F5 | Random forest | midseason phenology | Heading-stage phenology | All | 38.436 | 123147.836 | 0.047 |
| F5 | Random forest | weather sowing to heading | Weather: sowing to heading | Extreme | 46.786 | 476652.646 | 0.492 |
| F5 | Random forest | environment management | Environment and management | Extreme | 57.868 | 262025.924 | 0.271 |
| F5 | Random forest | blup core | Cultivar genetic potential | Extreme | 161.021 | 182275.66 | 0.188 |
| F5 | Random forest | midseason phenology | Heading-stage phenology | Extreme | 42.067 | 47619.908 | 0.049 |
| F5 | Random forest | weather sowing to heading | Weather: sowing to heading | Normal | 44.57 | 831146.724 | 0.501 |
| F5 | Random forest | environment management | Environment and management | Normal | 52.14 | 432133.665 | 0.261 |
| F5 | Random forest | blup core | Cultivar genetic potential | Normal | 153.855 | 318786.697 | 0.192 |
| F5 | Random forest | midseason phenology | Heading-stage phenology | Normal | 36.452 | 75527.927 | 0.046 |

**Supplementary Table 13.** Forward-year sensitivity after removing the numeric Year feature

| Feature set | Model | Variant | Variant label | Year splits | Feature count | Mean R^2^ | SD of R² | Mean RMSE | SD of RMSE | Mean MAE | SD of MAE | Mean normalized RMSE | SD of normalized RMSE | Mean Spearman correlation | SD of Spearman correlation | Mean top-20% recall | SD of top-20% recall |
| --- | --- | --- | --- | --- | --- | --- | --- | --- | --- | --- | --- | --- | --- | --- | --- | --- | --- |
| F1 | Random forest | year removed | Year removed | 6 | 13 | 0.055 | 0.288 | 821.973 | 106.493 | 635.983 | 64.872 | 0.11 | 0.015 | 0.343 | 0.129 | 0.445 | 0.176 |
| F1 | Random forest | year retained | Year retained | 6 | 14 | 0.068 | 0.257 | 817.94 | 95.477 | 636.287 | 58.492 | 0.109 | 0.013 | 0.35 | 0.125 | 0.482 | 0.158 |
| F2 | CatBoost | year removed | Year removed | 6 | 22 | 0.035 | 0.305 | 829.005 | 114.817 | 643.585 | 73.769 | 0.111 | 0.016 | 0.35 | 0.185 | 0.445 | 0.162 |
| F2 | CatBoost | year retained | Year retained | 6 | 23 | 0.106 | 0.214 | 802.113 | 78.019 | 627.925 | 52.581 | 0.107 | 0.011 | 0.362 | 0.171 | 0.454 | 0.174 |
| F3 | Random forest | year removed | Year removed | 6 | 23 | 0.071 | 0.242 | 816.825 | 82.66 | 641.927 | 48.917 | 0.109 | 0.012 | 0.355 | 0.152 | 0.484 | 0.159 |
| F3 | Random forest | year retained | Year retained | 6 | 24 | 0.086 | 0.253 | 808.951 | 85.518 | 634.12 | 49.605 | 0.108 | 0.012 | 0.37 | 0.141 | 0.469 | 0.156 |
| F4 | ElasticNet | year removed | Year removed | 6 | 33 | 0.365 | 0.196 | 673.933 | 86.955 | 524.425 | 63.104 | 0.09 | 0.012 | 0.607 | 0.132 | 0.587 | 0.126 |
| F4 | ElasticNet | year retained | Year retained | 6 | 34 | 0.359 | 0.232 | 673.605 | 93.758 | 525.059 | 68.595 | 0.09 | 0.013 | 0.61 | 0.134 | 0.578 | 0.121 |

**Supplementary Table 14.** Micro-averaged validation metrics

| Validation scheme | Feature set | Model | Records | R2 | Rmse | Mae | Nrmse | Spearman | Top20 recall |
| --- | --- | --- | --- | --- | --- | --- | --- | --- | --- |
| Forward-year prediction | F0 | Environment random-effect LMM | 2660 | -0.005 | 880.855 | 687.59 | 0.118 | 0.029 | 0.139 |
| Forward-year prediction | F0 | Environment mean | 2660 | -0.014 | 884.643 | 689.855 | 0.118 | -0.003 | 0.165 |
| Forward-year prediction | F0 | Overall mean | 2660 | -0.014 | 884.643 | 689.855 | 0.118 | -0.003 | 0.165 |
| Forward-year prediction | F1 | Random forest | 2660 | 0.132 | 818.478 | 634.677 | 0.109 | 0.355 | 0.487 |
| Forward-year prediction | F1 | ElasticNet | 2660 | 0.125 | 821.978 | 630.727 | 0.11 | 0.406 | 0.483 |
| Forward-year prediction | F1 | Ridge | 2660 | 0.12 | 824.116 | 633.778 | 0.11 | 0.405 | 0.483 |
| Forward-year prediction | F1 | CatBoost | 2660 | 0.119 | 824.868 | 637.475 | 0.11 | 0.326 | 0.397 |
| Forward-year prediction | F1 | LightGBM | 2660 | 0.109 | 829.357 | 635.855 | 0.111 | 0.327 | 0.447 |
| Forward-year prediction | F1 | XGBoost | 2660 | 0.095 | 835.897 | 638.055 | 0.112 | 0.313 | 0.398 |
| Forward-year prediction | F1 | LinearRegression | 2660 | 0.035 | 863.064 | 653.268 | 0.115 | 0.407 | 0.425 |
| Forward-year prediction | F2 | CatBoost | 2660 | 0.172 | 799.417 | 623.959 | 0.107 | 0.392 | 0.455 |
| Forward-year prediction | F2 | Random forest | 2660 | 0.147 | 811.714 | 631.621 | 0.108 | 0.38 | 0.502 |
| Forward-year prediction | F2 | ElasticNet | 2660 | 0.085 | 840.753 | 636.916 | 0.112 | 0.384 | 0.524 |
| Forward-year prediction | F2 | Ridge | 2660 | 0.073 | 846.121 | 641.799 | 0.113 | 0.379 | 0.519 |
| Forward-year prediction | F2 | XGBoost | 2660 | 0.067 | 848.732 | 659.01 | 0.113 | 0.337 | 0.434 |
| Forward-year prediction | F2 | LightGBM | 2660 | -0.036 | 894.235 | 696.875 | 0.119 | 0.327 | 0.445 |
| Forward-year prediction | F2 | LinearRegression | 2660 | -0.172 | 951.502 | 727.447 | 0.127 | 0.36 | 0.498 |
| Forward-year prediction | F3 | Random forest | 2660 | 0.155 | 807.781 | 631.68 | 0.108 | 0.382 | 0.479 |
| Forward-year prediction | F3 | CatBoost | 2660 | 0.144 | 813.221 | 634.126 | 0.108 | 0.366 | 0.462 |
| Forward-year prediction | F3 | XGBoost | 2660 | 0.091 | 837.811 | 656.805 | 0.112 | 0.322 | 0.444 |
| Forward-year prediction | F3 | ElasticNet | 2660 | 0.07 | 847.361 | 644.522 | 0.113 | 0.361 | 0.534 |
| Forward-year prediction | F3 | LightGBM | 2660 | 0.06 | 852.089 | 667.87 | 0.114 | 0.33 | 0.414 |
| Forward-year prediction | F3 | Ridge | 2660 | 0.055 | 854.267 | 651.419 | 0.114 | 0.354 | 0.53 |
| Forward-year prediction | F3 | LinearRegression | 2660 | -0.256 | 984.613 | 752.369 | 0.131 | 0.314 | 0.47 |
| Forward-year prediction | F4 | ElasticNet | 2660 | 0.413 | 673.389 | 522.03 | 0.09 | 0.597 | 0.59 |
| Forward-year prediction | F4 | Ridge | 2660 | 0.404 | 678.606 | 527.442 | 0.091 | 0.591 | 0.579 |
| Forward-year prediction | F4 | CatBoost | 2660 | 0.374 | 695.317 | 542.866 | 0.093 | 0.594 | 0.511 |
| Forward-year prediction | F4 | XGBoost | 2660 | 0.371 | 696.925 | 547.68 | 0.093 | 0.576 | 0.534 |
| Forward-year prediction | F4 | LightGBM | 2660 | 0.289 | 741.084 | 576.759 | 0.099 | 0.539 | 0.536 |
| Forward-year prediction | F4 | Random forest | 2660 | 0.275 | 748.073 | 588.483 | 0.1 | 0.496 | 0.519 |
| Forward-year prediction | F4 | LinearRegression | 2660 | 0.048 | 857.498 | 658.13 | 0.114 | 0.478 | 0.455 |
| Forward-year prediction | F5 | ElasticNet | 2660 | 0.111 | 828.675 | 633.851 | 0.111 | 0.398 | 0.53 |
| Forward-year prediction | F5 | Ridge | 2660 | 0.102 | 832.755 | 637.939 | 0.111 | 0.396 | 0.53 |
| Forward-year prediction | F5 | Random forest | 2660 | 0.055 | 854.365 | 666.863 | 0.114 | 0.324 | 0.415 |
| Forward-year prediction | F5 | CatBoost | 2660 | 0.02 | 869.718 | 674.692 | 0.116 | 0.318 | 0.41 |
| Forward-year prediction | F5 | LightGBM | 2660 | -0.015 | 885.493 | 684.412 | 0.118 | 0.291 | 0.397 |
| Forward-year prediction | F5 | XGBoost | 2660 | -0.021 | 887.964 | 681.202 | 0.118 | 0.304 | 0.419 |
| Forward-year prediction | F5 | LinearRegression | 2660 | -0.211 | 966.999 | 723.362 | 0.129 | 0.337 | 0.492 |
| Leave-one-site-out | F0 | Environment random-effect LMM | 3204 | -0.05 | 885.85 | 684.06 | 0.118 | -0.42 | 0.095 |
| Leave-one-site-out | F0 | Environment mean | 3204 | -0.06 | 890.186 | 686.6 | 0.119 | -0.412 | 0.131 |
| Leave-one-site-out | F0 | Overall mean | 3204 | -0.06 | 890.186 | 686.6 | 0.119 | -0.412 | 0.131 |
| Leave-one-site-out | F1 | ElasticNet | 3204 | 0.199 | 773.73 | 608.403 | 0.103 | 0.396 | 0.42 |
| Leave-one-site-out | F1 | Ridge | 3204 | 0.196 | 775.161 | 609.394 | 0.103 | 0.389 | 0.407 |
| Leave-one-site-out | F1 | LinearRegression | 3204 | 0.168 | 788.406 | 618.984 | 0.105 | 0.359 | 0.437 |
| Leave-one-site-out | F1 | Random forest | 3204 | 0.061 | 837.968 | 664.548 | 0.112 | 0.278 | 0.278 |
| Leave-one-site-out | F1 | CatBoost | 3204 | 0.037 | 848.399 | 676.447 | 0.113 | 0.226 | 0.281 |
| Leave-one-site-out | F1 | XGBoost | 3204 | 0.021 | 855.497 | 683.034 | 0.114 | 0.232 | 0.346 |
| Leave-one-site-out | F1 | LightGBM | 3204 | -0.065 | 892.27 | 710.697 | 0.119 | 0.138 | 0.24 |
| Leave-one-site-out | F2 | ElasticNet | 3204 | 0.155 | 794.682 | 622.596 | 0.106 | 0.366 | 0.396 |
| Leave-one-site-out | F2 | Ridge | 3204 | 0.145 | 799.297 | 625.342 | 0.107 | 0.355 | 0.373 |
| Leave-one-site-out | F2 | LinearRegression | 3204 | 0.102 | 819.226 | 637.656 | 0.109 | 0.322 | 0.398 |
| Leave-one-site-out | F2 | Random forest | 3204 | 0.082 | 828.604 | 653.505 | 0.111 | 0.293 | 0.323 |
| Leave-one-site-out | F2 | CatBoost | 3204 | 0.051 | 842.052 | 666.919 | 0.112 | 0.263 | 0.296 |
| Leave-one-site-out | F2 | LightGBM | 3204 | 0.038 | 848.077 | 673.246 | 0.113 | 0.26 | 0.262 |
| Leave-one-site-out | F2 | XGBoost | 3204 | -0.018 | 872.145 | 690.328 | 0.116 | 0.212 | 0.245 |
| Leave-one-site-out | F3 | ElasticNet | 3204 | 0.186 | 779.888 | 613.989 | 0.104 | 0.42 | 0.373 |
| Leave-one-site-out | F3 | Ridge | 3204 | 0.177 | 784.554 | 617.413 | 0.105 | 0.411 | 0.37 |
| Leave-one-site-out | F3 | LinearRegression | 3204 | 0.145 | 799.627 | 627.209 | 0.107 | 0.387 | 0.396 |
| Leave-one-site-out | F3 | Random forest | 3204 | 0.11 | 815.499 | 639.295 | 0.109 | 0.347 | 0.318 |
| Leave-one-site-out | F3 | CatBoost | 3204 | 0.1 | 820.145 | 646.161 | 0.109 | 0.345 | 0.306 |
| Leave-one-site-out | F3 | XGBoost | 3204 | 0.064 | 836.407 | 662.846 | 0.112 | 0.318 | 0.315 |
| Leave-one-site-out | F3 | LightGBM | 3204 | 0.06 | 838.395 | 662.83 | 0.112 | 0.313 | 0.296 |
| Leave-one-site-out | F4 | ElasticNet | 3204 | 0.461 | 634.587 | 495.532 | 0.085 | 0.648 | 0.543 |
| Leave-one-site-out | F4 | Ridge | 3204 | 0.452 | 639.85 | 500.346 | 0.085 | 0.643 | 0.532 |
| Leave-one-site-out | F4 | LightGBM | 3204 | 0.426 | 654.962 | 518.15 | 0.087 | 0.626 | 0.516 |
| Leave-one-site-out | F4 | CatBoost | 3204 | 0.425 | 655.601 | 519.243 | 0.087 | 0.628 | 0.501 |
| Leave-one-site-out | F4 | XGBoost | 3204 | 0.418 | 659.333 | 521.163 | 0.088 | 0.624 | 0.496 |
| Leave-one-site-out | F4 | LinearRegression | 3204 | 0.417 | 660.363 | 517.559 | 0.088 | 0.624 | 0.524 |
| Leave-one-site-out | F4 | Random forest | 3204 | 0.261 | 743.042 | 581.872 | 0.099 | 0.522 | 0.384 |
| Leave-one-site-out | F5 | ElasticNet | 3204 | 0.133 | 804.997 | 623.67 | 0.107 | 0.381 | 0.365 |
| Leave-one-site-out | F5 | Ridge | 3204 | 0.123 | 809.841 | 627.408 | 0.108 | 0.369 | 0.357 |
| Leave-one-site-out | F5 | CatBoost | 3204 | 0.068 | 834.695 | 651.064 | 0.111 | 0.329 | 0.318 |
| Leave-one-site-out | F5 | Random forest | 3204 | 0.057 | 839.647 | 655.463 | 0.112 | 0.3 | 0.289 |
| Leave-one-site-out | F5 | LinearRegression | 3204 | 0.006 | 862.032 | 664.415 | 0.115 | 0.257 | 0.295 |
| Leave-one-site-out | F5 | XGBoost | 3204 | -0.009 | 868.477 | 671.61 | 0.116 | 0.242 | 0.253 |
| Leave-one-site-out | F5 | LightGBM | 3204 | -0.021 | 873.487 | 682.815 | 0.116 | 0.236 | 0.245 |
| Leave-one-site-year-out | F0 | Environment random-effect LMM | 3204 | -0.014 | 870.553 | 672.885 | 0.116 | -0.604 | 0.034 |
| Leave-one-site-year-out | F0 | Environment mean | 3204 | -0.016 | 871.376 | 673.027 | 0.116 | -0.592 | 0.048 |
| Leave-one-site-year-out | F0 | Overall mean | 3204 | -0.016 | 871.376 | 673.027 | 0.116 | -0.592 | 0.048 |
| Leave-one-site-year-out | F1 | ElasticNet | 3204 | 0.212 | 767.373 | 592.723 | 0.102 | 0.442 | 0.488 |
| Leave-one-site-year-out | F1 | Ridge | 3204 | 0.201 | 772.693 | 596.319 | 0.103 | 0.441 | 0.488 |
| Leave-one-site-year-out | F1 | XGBoost | 3204 | 0.171 | 787.255 | 619.894 | 0.105 | 0.354 | 0.499 |
| Leave-one-site-year-out | F1 | Random forest | 3204 | 0.169 | 788.375 | 614.364 | 0.105 | 0.368 | 0.488 |
| Leave-one-site-year-out | F1 | LinearRegression | 3204 | 0.167 | 789.122 | 606.473 | 0.105 | 0.436 | 0.485 |
| Leave-one-site-year-out | F1 | LightGBM | 3204 | 0.14 | 801.611 | 621.986 | 0.107 | 0.337 | 0.473 |
| Leave-one-site-year-out | F1 | CatBoost | 3204 | 0.107 | 817.254 | 638.182 | 0.109 | 0.322 | 0.432 |
| Leave-one-site-year-out | F2 | ElasticNet | 3204 | 0.173 | 786.104 | 601.783 | 0.105 | 0.413 | 0.49 |
| Leave-one-site-year-out | F2 | Random forest | 3204 | 0.169 | 788.061 | 614.285 | 0.105 | 0.386 | 0.468 |
| Leave-one-site-year-out | F2 | CatBoost | 3204 | 0.165 | 789.966 | 621.984 | 0.105 | 0.365 | 0.47 |
| Leave-one-site-year-out | F2 | Ridge | 3204 | 0.158 | 793.411 | 605.598 | 0.106 | 0.403 | 0.493 |
| Leave-one-site-year-out | F2 | LightGBM | 3204 | 0.154 | 795.471 | 626.687 | 0.106 | 0.354 | 0.471 |
| Leave-one-site-year-out | F2 | XGBoost | 3204 | 0.136 | 803.801 | 633.495 | 0.107 | 0.35 | 0.445 |
| Leave-one-site-year-out | F2 | LinearRegression | 3204 | 0.111 | 815.043 | 615.251 | 0.109 | 0.383 | 0.477 |
| Leave-one-site-year-out | F3 | ElasticNet | 3204 | 0.227 | 760.168 | 583.022 | 0.101 | 0.474 | 0.499 |
| Leave-one-site-year-out | F3 | Random forest | 3204 | 0.221 | 763.014 | 593.362 | 0.102 | 0.434 | 0.465 |
| Leave-one-site-year-out | F3 | CatBoost | 3204 | 0.216 | 765.593 | 598.025 | 0.102 | 0.434 | 0.474 |
| Leave-one-site-year-out | F3 | Ridge | 3204 | 0.21 | 768.341 | 587.508 | 0.102 | 0.466 | 0.501 |
| Leave-one-site-year-out | F3 | XGBoost | 3204 | 0.188 | 779.047 | 612.906 | 0.104 | 0.412 | 0.449 |
| Leave-one-site-year-out | F3 | LightGBM | 3204 | 0.184 | 780.999 | 610.344 | 0.104 | 0.419 | 0.437 |
| Leave-one-site-year-out | F3 | LinearRegression | 3204 | 0.162 | 791.673 | 600.38 | 0.106 | 0.446 | 0.496 |
| Leave-one-site-year-out | F4 | ElasticNet | 3204 | 0.523 | 596.991 | 461.332 | 0.08 | 0.687 | 0.573 |
| Leave-one-site-year-out | F4 | Ridge | 3204 | 0.514 | 603.01 | 466.008 | 0.08 | 0.681 | 0.565 |
| Leave-one-site-year-out | F4 | XGBoost | 3204 | 0.51 | 605.141 | 472.821 | 0.081 | 0.671 | 0.59 |
| Leave-one-site-year-out | F4 | CatBoost | 3204 | 0.496 | 614.046 | 482.048 | 0.082 | 0.659 | 0.583 |
| Leave-one-site-year-out | F4 | LinearRegression | 3204 | 0.487 | 619.528 | 477.866 | 0.083 | 0.663 | 0.541 |
| Leave-one-site-year-out | F4 | LightGBM | 3204 | 0.485 | 620.365 | 485.292 | 0.083 | 0.648 | 0.587 |
| Leave-one-site-year-out | F4 | Random forest | 3204 | 0.379 | 681.412 | 535.231 | 0.091 | 0.57 | 0.549 |
| Leave-one-site-year-out | F5 | ElasticNet | 3204 | 0.249 | 749.404 | 573.685 | 0.1 | 0.487 | 0.484 |
| Leave-one-site-year-out | F5 | Ridge | 3204 | 0.241 | 753.153 | 576.147 | 0.1 | 0.484 | 0.477 |
| Leave-one-site-year-out | F5 | LinearRegression | 3204 | 0.216 | 765.367 | 584.297 | 0.102 | 0.478 | 0.474 |
| Leave-one-site-year-out | F5 | Random forest | 3204 | 0.11 | 815.483 | 629.068 | 0.109 | 0.368 | 0.417 |
| Leave-one-site-year-out | F5 | CatBoost | 3204 | 0.084 | 827.419 | 640.739 | 0.11 | 0.361 | 0.417 |
| Leave-one-site-year-out | F5 | XGBoost | 3204 | 0.053 | 841.569 | 648.584 | 0.112 | 0.337 | 0.385 |
| Leave-one-site-year-out | F5 | LightGBM | 3204 | 0 | 864.771 | 667.201 | 0.115 | 0.297 | 0.343 |
| Leave-one-year-out | F0 | Environment random-effect LMM | 3204 | -0.011 | 869.275 | 672.056 | 0.116 | -0.156 | 0.172 |
| Leave-one-year-out | F0 | Environment mean | 3204 | -0.012 | 869.672 | 671.573 | 0.116 | -0.153 | 0.173 |
| Leave-one-year-out | F0 | Overall mean | 3204 | -0.012 | 869.672 | 671.573 | 0.116 | -0.153 | 0.173 |
| Leave-one-year-out | F1 | ElasticNet | 3204 | 0.233 | 757.038 | 581.983 | 0.101 | 0.469 | 0.504 |
| Leave-one-year-out | F1 | Ridge | 3204 | 0.223 | 761.954 | 585.356 | 0.102 | 0.47 | 0.498 |
| Leave-one-year-out | F1 | LinearRegression | 3204 | 0.188 | 779.206 | 597.058 | 0.104 | 0.47 | 0.491 |
| Leave-one-year-out | F1 | LightGBM | 3204 | 0.185 | 780.501 | 600.412 | 0.104 | 0.387 | 0.479 |
| Leave-one-year-out | F1 | Random forest | 3204 | 0.182 | 782.2 | 606.914 | 0.104 | 0.389 | 0.466 |
| Leave-one-year-out | F1 | XGBoost | 3204 | 0.173 | 786.24 | 608.808 | 0.105 | 0.388 | 0.479 |
| Leave-one-year-out | F1 | CatBoost | 3204 | 0.144 | 799.814 | 617.054 | 0.107 | 0.373 | 0.493 |
| Leave-one-year-out | F2 | ElasticNet | 3204 | 0.206 | 770.62 | 587.749 | 0.103 | 0.442 | 0.499 |
| Leave-one-year-out | F2 | Ridge | 3204 | 0.194 | 776.11 | 590.989 | 0.104 | 0.437 | 0.501 |
| Leave-one-year-out | F2 | CatBoost | 3204 | 0.189 | 778.811 | 605.782 | 0.104 | 0.386 | 0.465 |
| Leave-one-year-out | F2 | LightGBM | 3204 | 0.181 | 782.464 | 614.388 | 0.104 | 0.384 | 0.473 |
| Leave-one-year-out | F2 | XGBoost | 3204 | 0.178 | 783.701 | 611 | 0.105 | 0.385 | 0.479 |
| Leave-one-year-out | F2 | Random forest | 3204 | 0.171 | 787.155 | 608.131 | 0.105 | 0.393 | 0.448 |
| Leave-one-year-out | F2 | LinearRegression | 3204 | 0.133 | 804.879 | 607.072 | 0.107 | 0.414 | 0.493 |
| Leave-one-year-out | F3 | ElasticNet | 3204 | 0.209 | 768.917 | 587.3 | 0.103 | 0.441 | 0.493 |
| Leave-one-year-out | F3 | CatBoost | 3204 | 0.202 | 772.486 | 601.365 | 0.103 | 0.415 | 0.477 |
| Leave-one-year-out | F3 | Ridge | 3204 | 0.197 | 774.793 | 589.969 | 0.103 | 0.436 | 0.499 |
| Leave-one-year-out | F3 | Random forest | 3204 | 0.194 | 776.202 | 604.171 | 0.104 | 0.404 | 0.46 |
| Leave-one-year-out | F3 | XGBoost | 3204 | 0.184 | 781.162 | 610.566 | 0.104 | 0.391 | 0.473 |
| Leave-one-year-out | F3 | LightGBM | 3204 | 0.152 | 796.243 | 622.165 | 0.106 | 0.381 | 0.438 |
| Leave-one-year-out | F3 | LinearRegression | 3204 | 0.139 | 802.227 | 605.052 | 0.107 | 0.42 | 0.488 |
| Leave-one-year-out | F4 | ElasticNet | 3204 | 0.51 | 604.932 | 465.26 | 0.081 | 0.679 | 0.594 |
| Leave-one-year-out | F4 | Ridge | 3204 | 0.502 | 610.398 | 469.708 | 0.081 | 0.675 | 0.583 |
| Leave-one-year-out | F4 | XGBoost | 3204 | 0.485 | 620.461 | 484.738 | 0.083 | 0.652 | 0.598 |
| Leave-one-year-out | F4 | LightGBM | 3204 | 0.474 | 627.236 | 492.531 | 0.084 | 0.639 | 0.577 |
| Leave-one-year-out | F4 | CatBoost | 3204 | 0.471 | 629.14 | 494.245 | 0.084 | 0.641 | 0.582 |
| Leave-one-year-out | F4 | LinearRegression | 3204 | 0.452 | 639.778 | 494.941 | 0.085 | 0.642 | 0.552 |
| Leave-one-year-out | F4 | Random forest | 3204 | 0.35 | 697.06 | 545.031 | 0.093 | 0.553 | 0.526 |
| Leave-one-year-out | F5 | ElasticNet | 3204 | 0.178 | 783.85 | 600.498 | 0.105 | 0.413 | 0.477 |
| Leave-one-year-out | F5 | Ridge | 3204 | 0.172 | 786.554 | 601.343 | 0.105 | 0.413 | 0.47 |
| Leave-one-year-out | F5 | LinearRegression | 3204 | 0.15 | 797.054 | 608.882 | 0.106 | 0.407 | 0.456 |
| Leave-one-year-out | F5 | Random forest | 3204 | 0.061 | 837.983 | 648.456 | 0.112 | 0.306 | 0.378 |
| Leave-one-year-out | F5 | CatBoost | 3204 | 0.013 | 858.987 | 662.008 | 0.115 | 0.297 | 0.381 |
| Leave-one-year-out | F5 | XGBoost | 3204 | -0.051 | 886.533 | 676.51 | 0.118 | 0.263 | 0.363 |
| Leave-one-year-out | F5 | LightGBM | 3204 | -0.082 | 899.526 | 691.828 | 0.12 | 0.221 | 0.346 |

**Supplementary Table 15.** Paired tests for feature-set and algorithm contrasts

| Validation scheme | Contrast type | Left | Right | Left model | Right model | Metric | N pairs | Mean difference | Ci95 low | Ci95 high | Paired t p | Wilcoxon p | Feature set |
| --- | --- | --- | --- | --- | --- | --- | --- | --- | --- | --- | --- | --- | --- |
| Random five-fold CV | feature set best model | F1 | F2 | LightGBM | Random forest | r2 | 5 | 0.039 | 0.025 | 0.052 | 0.001 | 0.062 |  |
| Random five-fold CV | feature set best model | F2 | F3 | Random forest | LightGBM | r2 | 5 | 0.054 | 0.043 | 0.065 | 0 | 0.062 |  |
| Random five-fold CV | feature set best model | F3 | F4 | LightGBM | LightGBM | r2 | 5 | 0.089 | 0.064 | 0.114 | 0.001 | 0.062 |  |
| Random five-fold CV | leading algorithm within feature set | Random forest | LightGBM | Random forest | LightGBM | r2 | 5 | 0.004 | -0.001 | 0.008 | 0.113 | 0.188 | F1 |
| Random five-fold CV | leading algorithm within feature set | LightGBM | Random forest | LightGBM | Random forest | r2 | 5 | 0.001 | -0.016 | 0.018 | 0.845 | 1 | F2 |
| Random five-fold CV | leading algorithm within feature set | Random forest | LightGBM | Random forest | LightGBM | r2 | 5 | 0.003 | -0.004 | 0.011 | 0.31 | 0.312 | F3 |
| Random five-fold CV | leading algorithm within feature set | XGBoost | LightGBM | XGBoost | LightGBM | r2 | 5 | 0.02 | 0.012 | 0.027 | 0.002 | 0.062 | F4 |
| Random five-fold CV | leading algorithm within feature set | LightGBM | Random forest | LightGBM | Random forest | r2 | 5 | 0.004 | -0.004 | 0.011 | 0.223 | 0.312 | F5 |
| Leave-one-year-out | feature set best model | F1 | F2 | ElasticNet | ElasticNet | r2 | 7 | -0.03 | -0.049 | -0.011 | 0.008 | 0.031 |  |
| Leave-one-year-out | feature set best model | F2 | F3 | ElasticNet | CatBoost | r2 | 7 | 0.013 | -0.161 | 0.186 | 0.866 | 0.812 |  |
| Leave-one-year-out | feature set best model | F3 | F4 | CatBoost | ElasticNet | r2 | 7 | 0.323 | 0.162 | 0.484 | 0.003 | 0.016 |  |
| Leave-one-year-out | leading algorithm within feature set | Ridge | ElasticNet | Ridge | ElasticNet | r2 | 7 | 0.012 | -0.006 | 0.029 | 0.153 | 0.297 | F1 |
| Leave-one-year-out | leading algorithm within feature set | Ridge | ElasticNet | Ridge | ElasticNet | r2 | 7 | 0.014 | -0.002 | 0.029 | 0.077 | 0.109 | F2 |
| Leave-one-year-out | leading algorithm within feature set | CatBoost | ElasticNet | CatBoost | ElasticNet | r2 | 7 | -0.004 | -0.157 | 0.149 | 0.951 | 0.688 | F3 |
| Leave-one-year-out | leading algorithm within feature set | Ridge | ElasticNet | Ridge | ElasticNet | r2 | 7 | 0.01 | -0.002 | 0.021 | 0.094 | 0.078 | F4 |
| Leave-one-year-out | leading algorithm within feature set | Ridge | ElasticNet | Ridge | ElasticNet | r2 | 7 | 0.008 | -0.008 | 0.023 | 0.268 | 0.578 | F5 |
| Forward-year prediction | feature set best model | F1 | F2 | Random forest | CatBoost | r2 | 6 | 0.038 | -0.09 | 0.166 | 0.482 | 0.438 |  |
| Forward-year prediction | feature set best model | F2 | F3 | CatBoost | Random forest | r2 | 6 | -0.02 | -0.106 | 0.066 | 0.574 | 0.562 |  |
| Forward-year prediction | feature set best model | F3 | F4 | Random forest | ElasticNet | r2 | 6 | 0.273 | 0.042 | 0.504 | 0.029 | 0.031 |  |
| Forward-year prediction | leading algorithm within feature set | ElasticNet | Random forest | ElasticNet | Random forest | r2 | 6 | 0.037 | -0.204 | 0.279 | 0.709 | 1 | F1 |
| Forward-year prediction | leading algorithm within feature set | Random forest | CatBoost | Random forest | CatBoost | r2 | 6 | 0.027 | -0.052 | 0.107 | 0.417 | 0.438 | F2 |
| Forward-year prediction | leading algorithm within feature set | CatBoost | Random forest | CatBoost | Random forest | r2 | 6 | 0.015 | -0.054 | 0.084 | 0.594 | 0.844 | F3 |
| Forward-year prediction | leading algorithm within feature set | Ridge | ElasticNet | Ridge | ElasticNet | r2 | 6 | 0.01 | -0.004 | 0.025 | 0.116 | 0.094 | F4 |
| Forward-year prediction | leading algorithm within feature set | Ridge | ElasticNet | Ridge | ElasticNet | r2 | 6 | 0.01 | -0.004 | 0.024 | 0.131 | 0.156 | F5 |
| Leave-one-site-out | feature set best model | F1 | F2 | ElasticNet | ElasticNet | r2 | 10 | -0.044 | -0.109 | 0.02 | 0.154 | 0.16 |  |
| Leave-one-site-out | feature set best model | F2 | F3 | ElasticNet | ElasticNet | r2 | 10 | 0.057 | 0.012 | 0.103 | 0.018 | 0.084 |  |
| Leave-one-site-out | feature set best model | F3 | F4 | ElasticNet | ElasticNet | r2 | 10 | 0.307 | 0.163 | 0.451 | 0.001 | 0.01 |  |
| Leave-one-site-out | leading algorithm within feature set | Ridge | ElasticNet | Ridge | ElasticNet | r2 | 10 | 0.009 | -0.005 | 0.024 | 0.178 | 0.275 | F1 |
| Leave-one-site-out | leading algorithm within feature set | Ridge | ElasticNet | Ridge | ElasticNet | r2 | 10 | 0.018 | -0.001 | 0.038 | 0.063 | 0.105 | F2 |
| Leave-one-site-out | leading algorithm within feature set | Ridge | ElasticNet | Ridge | ElasticNet | r2 | 10 | 0.018 | -0.001 | 0.038 | 0.064 | 0.064 | F3 |
| Leave-one-site-out | leading algorithm within feature set | Ridge | ElasticNet | Ridge | ElasticNet | r2 | 10 | 0.012 | 0.003 | 0.021 | 0.018 | 0.01 | F4 |
| Leave-one-site-out | leading algorithm within feature set | Ridge | ElasticNet | Ridge | ElasticNet | r2 | 10 | 0.014 | -0.001 | 0.03 | 0.065 | 0.193 | F5 |
| Leave-one-site-year-out | feature set best model | F1 | F2 | ElasticNet | ElasticNet | r2 | 67 | -0.052 | -0.118 | 0.015 | 0.127 | 0.115 |  |
| Leave-one-site-year-out | feature set best model | F2 | F3 | ElasticNet | ElasticNet | r2 | 67 | 0.046 | -0.047 | 0.14 | 0.325 | 0.001 |  |
| Leave-one-site-year-out | feature set best model | F3 | F4 | ElasticNet | ElasticNet | r2 | 67 | 0.559 | 0.354 | 0.763 | 0 | 0 |  |
| Leave-one-site-year-out | leading algorithm within feature set | Ridge | ElasticNet | Ridge | ElasticNet | r2 | 67 | 0.019 | 0.003 | 0.036 | 0.024 | 0.079 | F1 |
| Leave-one-site-year-out | leading algorithm within feature set | Random forest | ElasticNet | Random forest | ElasticNet | r2 | 67 | 0.09 | -0.136 | 0.316 | 0.43 | 0.439 | F2 |
| Leave-one-site-year-out | leading algorithm within feature set | Random forest | ElasticNet | Random forest | ElasticNet | r2 | 67 | 0.031 | -0.223 | 0.285 | 0.806 | 0.442 | F3 |
| Leave-one-site-year-out | leading algorithm within feature set | Ridge | ElasticNet | Ridge | ElasticNet | r2 | 67 | 0.025 | 0.008 | 0.042 | 0.005 | 0.008 | F4 |
| Leave-one-site-year-out | leading algorithm within feature set | Ridge | ElasticNet | Ridge | ElasticNet | r2 | 67 | 0.027 | -0.002 | 0.056 | 0.066 | 0.261 | F5 |

**Supplementary Table 16.** Environment-level R^2^ and within-environment yield variance

| Held out environment | Feature set | Model | Train records | Test records | Runtime seconds | R2 | Rmse | Mae | Nrmse | Spearman | Top20 recall | Env | Yield sd | Yield mean | Yield range | Records |
| --- | --- | --- | --- | --- | --- | --- | --- | --- | --- | --- | --- | --- | --- | --- | --- | --- |
| QinZhou 2018 | F4 | ElasticNet | 3130 | 74 | 0.33 | -6.14 | 573.752 | 540.764 | 0.077 | -0.039 | 0.375 | QinZhou 2018 | 216.181 | 7404.634 | 759.151 | 74 |
| HuiZhou 2019 | F4 | ElasticNet | 3179 | 25 | 0.346 | -3.919 | 992.625 | 909.964 | 0.155 | 0.609 | 0.4 | HuiZhou 2019 | 456.787 | 6396.528 | 2260.65 | 25 |
| GuangZhou 2016 | F4 | ElasticNet | 3128 | 76 | 0.2 | -2.346 | 605.761 | 530.864 | 0.085 | -0.155 | 0.118 | GuangZhou 2016 | 333.362 | 7152.6 | 1602.45 | 76 |
| GuangZhou 2019 | F4 | ElasticNet | 3154 | 50 | 0.336 | -1.193 | 622.391 | 551.373 | 0.082 | 0.592 | 0.6 | GuangZhou 2019 | 424.542 | 7587.144 | 1589.25 | 50 |
| HuiZhou 2018 | F4 | ElasticNet | 3167 | 37 | 0.315 | -1.185 | 927.454 | 842.291 | 0.112 | 0.566 | 0.5 | HuiZhou 2018 | 636.031 | 8269.931 | 2357.368 | 37 |
| NanNing 2016 | F4 | ElasticNet | 3128 | 76 | 0.152 | -0.912 | 389.894 | 320.52 | 0.049 | 0.404 | 0.375 | NanNing 2016 | 283.816 | 7901.211 | 1247.1 | 76 |
| GuangZhou 2017 | F4 | ElasticNet | 3138 | 66 | 0.362 | -0.885 | 483.303 | 380.383 | 0.065 | 0.481 | 0.429 | GuangZhou 2017 | 354.737 | 7448.786 | 1312.5 | 66 |
| QinZhou 2017 | F4 | ElasticNet | 3138 | 66 | 0.322 | -0.829 | 349.364 | 284.068 | 0.046 | 0.615 | 0.571 | QinZhou 2017 | 260.304 | 7625.009 | 874.95 | 66 |
| ZhangZhou 2021 | F4 | ElasticNet | 3132 | 72 | 0.234 | -0.687 | 748.531 | 681.949 | 0.088 | 0.642 | 0.556 | ZhangZhou 2021 | 580.423 | 8495.054 | 2459.85 | 72 |
| ZhangZhou 2017 | F4 | ElasticNet | 3139 | 65 | 0.326 | -0.617 | 1019.569 | 792.305 | 0.112 | 0.274 | 0.286 | ZhangZhou 2017 | 808.024 | 9083.275 | 3024.3 | 65 |
| QingYuan 2019 | F4 | ElasticNet | 3179 | 25 | 0.35 | -0.562 | 421.083 | 333.394 | 0.061 | 0.27 | 0.4 | QingYuan 2019 | 343.91 | 6869.622 | 1286.85 | 25 |
| YuLin 2016 | F4 | ElasticNet | 3166 | 38 | 0.311 | -0.462 | 514.312 | 412.533 | 0.065 | -0.047 | 0 | YuLin 2016 | 430.996 | 7878.805 | 1639.95 | 38 |
| YuLin 2022 | F4 | ElasticNet | 3170 | 34 | 0.308 | -0.436 | 685.331 | 522.127 | 0.093 | 0.234 | 0.286 | YuLin 2022 | 580.487 | 7382.951 | 2294.4 | 34 |
| GuangZhou 2022 | F4 | ElasticNet | 3137 | 67 | 0.297 | -0.382 | 596.588 | 499.552 | 0.094 | 0.62 | 0.571 | GuangZhou 2022 | 511.223 | 6339.304 | 1913.25 | 67 |
| HaiKou 2019 | F4 | ElasticNet | 3130 | 74 | 0.295 | -0.298 | 1063.545 | 846.422 | 0.154 | 0.333 | 0.5 | HaiKou 2019 | 939.733 | 6911.372 | 3873 | 74 |
| NanNing 2021 | F4 | ElasticNet | 3132 | 72 | 0.39 | -0.293 | 565.756 | 446.133 | 0.077 | 0.705 | 0.5 | NanNing 2021 | 501.007 | 7381.229 | 1925.85 | 72 |
| QingYuan 2016 | F4 | ElasticNet | 3166 | 38 | 0.322 | -0.288 | 519.316 | 408.496 | 0.07 | 0.422 | 0.375 | QingYuan 2016 | 463.686 | 7378.942 | 1962.6 | 38 |
| ZhangZhou 2018 | F4 | ElasticNet | 3156 | 48 | 0.253 | -0.225 | 668.972 | 560.756 | 0.072 | 0.536 | 0.4 | ZhangZhou 2018 | 610.722 | 9251.358 | 2466.279 | 48 |
| ZhangZhou 2020 | F4 | ElasticNet | 3142 | 62 | 0.34 | -0.22 | 1252.438 | 1060.646 | 0.172 | 0.545 | 0.571 | ZhangZhou 2020 | 1143.01 | 7267.29 | 4130.455 | 62 |
| GuangZhou 2021 | F4 | ElasticNet | 3132 | 72 | 0.288 | -0.213 | 544.514 | 418.474 | 0.083 | 0.638 | 0.438 | GuangZhou 2021 | 497.837 | 6555.358 | 1853.1 | 72 |

**Supplementary Table 17.** Seen and unseen cultivar performance

| Validation scheme | Feature set | Model | Cultivar history class | Splits | Records total | Mean R2 | SD of R2 | Mean RMSE | SD of RMSE | Mean MAE | SD of MAE | Mean normalized RMSE | SD of normalized RMSE | Mean Spearman correlation | SD of Spearman correlation | Mean top-20% recall | SD of top-20% recall |
| --- | --- | --- | --- | --- | --- | --- | --- | --- | --- | --- | --- | --- | --- | --- | --- | --- | --- |
| Forward-year prediction | F0 | Environment mean | Seen cultivars | 6 | 1001 | -0.087 | 0.102 | 866.72 | 92.858 | 661.125 | 70.415 | 0.114 | 0.011 |  |  | 0.2 | 0.065 |
| Forward-year prediction | F0 | Environment mean | Unseen cultivars | 6 | 1659 | -0.068 | 0.058 | 889.509 | 55.137 | 701.084 | 56.49 | 0.12 | 0.007 |  |  | 0.202 | 0.046 |
| Forward-year prediction | F0 | Environment random-effect LMM | Seen cultivars | 6 | 1001 | -0.085 | 0.085 | 866.404 | 93.103 | 662.618 | 70.991 | 0.114 | 0.011 |  |  | 0.2 | 0.065 |
| Forward-year prediction | F0 | Environment random-effect LMM | Unseen cultivars | 6 | 1659 | -0.051 | 0.04 | 883.25 | 58.413 | 696.393 | 54.891 | 0.119 | 0.007 |  |  | 0.202 | 0.046 |
| Forward-year prediction | F0 | Overall mean | Seen cultivars | 6 | 1001 | -0.087 | 0.102 | 866.72 | 92.858 | 661.125 | 70.415 | 0.114 | 0.011 |  |  | 0.2 | 0.065 |
| Forward-year prediction | F0 | Overall mean | Unseen cultivars | 6 | 1659 | -0.068 | 0.058 | 889.509 | 55.137 | 701.084 | 56.49 | 0.12 | 0.007 |  |  | 0.202 | 0.046 |
| Forward-year prediction | F1 | CatBoost | Seen cultivars | 6 | 1001 | 0.048 | 0.211 | 800.87 | 40.956 | 619.165 | 46.536 | 0.105 | 0.005 | 0.331 | 0.181 | 0.451 | 0.197 |
| Forward-year prediction | F1 | CatBoost | Unseen cultivars | 6 | 1659 | 0.048 | 0.245 | 833.536 | 105.343 | 645.969 | 61.285 | 0.112 | 0.015 | 0.286 | 0.149 | 0.421 | 0.142 |
| Forward-year prediction | F1 | ElasticNet | Seen cultivars | 6 | 1001 | 0.067 | 0.439 | 771.384 | 114.967 | 600.798 | 89.645 | 0.102 | 0.017 | 0.409 | 0.132 | 0.451 | 0.232 |
| Forward-year prediction | F1 | ElasticNet | Unseen cultivars | 6 | 1659 | -0.012 | 0.457 | 843.679 | 164.952 | 655.001 | 107.306 | 0.114 | 0.024 | 0.37 | 0.155 | 0.409 | 0.149 |
| Forward-year prediction | F1 | LightGBM | Seen cultivars | 6 | 1001 | 0.051 | 0.322 | 790.6 | 77.065 | 603.189 | 42.646 | 0.104 | 0.012 | 0.361 | 0.198 | 0.46 | 0.249 |
| Forward-year prediction | F1 | LightGBM | Unseen cultivars | 6 | 1659 | 0.017 | 0.268 | 843.579 | 97.588 | 652 | 55.102 | 0.114 | 0.015 | 0.304 | 0.14 | 0.403 | 0.172 |
| Forward-year prediction | F1 | LinearRegression | Seen cultivars | 6 | 1001 | -0.069 | 0.428 | 834.124 | 104.666 | 643.558 | 67.463 | 0.11 | 0.014 | 0.381 | 0.146 | 0.418 | 0.199 |
| Forward-year prediction | F1 | LinearRegression | Unseen cultivars | 6 | 1659 | -0.081 | 0.469 | 875.436 | 172.502 | 666.92 | 105.318 | 0.118 | 0.025 | 0.373 | 0.157 | 0.391 | 0.165 |
| Forward-year prediction | F1 | Random forest | Seen cultivars | 6 | 1001 | 0.052 | 0.27 | 795.626 | 59.019 | 616.942 | 65.581 | 0.105 | 0.008 | 0.367 | 0.155 | 0.509 | 0.223 |
| Forward-year prediction | F1 | Random forest | Unseen cultivars | 6 | 1659 | 0.061 | 0.277 | 825.793 | 118.685 | 644.336 | 64.038 | 0.111 | 0.017 | 0.336 | 0.12 | 0.448 | 0.132 |
| Forward-year prediction | F1 | Ridge | Seen cultivars | 6 | 1001 | 0.059 | 0.45 | 773.377 | 119.499 | 604.446 | 89.002 | 0.102 | 0.018 | 0.408 | 0.135 | 0.438 | 0.226 |
| Forward-year prediction | F1 | Ridge | Unseen cultivars | 6 | 1659 | -0.015 | 0.461 | 844.879 | 167.249 | 657.595 | 107.08 | 0.114 | 0.024 | 0.359 | 0.155 | 0.403 | 0.153 |
| Forward-year prediction | F1 | XGBoost | Seen cultivars | 6 | 1001 | 0.042 | 0.243 | 801.583 | 53.722 | 606.635 | 29.91 | 0.105 | 0.008 | 0.323 | 0.173 | 0.392 | 0.187 |
| Forward-year prediction | F1 | XGBoost | Unseen cultivars | 6 | 1659 | -0.003 | 0.258 | 852.43 | 86.771 | 655.313 | 49.546 | 0.115 | 0.013 | 0.279 | 0.113 | 0.379 | 0.118 |
| Forward-year prediction | F2 | CatBoost | Seen cultivars | 6 | 1001 | 0.079 | 0.215 | 787.654 | 65.743 | 610.195 | 59.47 | 0.103 | 0.008 | 0.388 | 0.188 | 0.473 | 0.228 |
| Forward-year prediction | F2 | CatBoost | Unseen cultivars | 6 | 1659 | 0.103 | 0.233 | 807.68 | 97.979 | 635.847 | 64.696 | 0.109 | 0.014 | 0.345 | 0.17 | 0.429 | 0.135 |
| Forward-year prediction | F2 | ElasticNet | Seen cultivars | 6 | 1001 | 0.034 | 0.557 | 773.382 | 162.571 | 600.691 | 128.878 | 0.102 | 0.024 | 0.411 | 0.17 | 0.507 | 0.235 |
| Forward-year prediction | F2 | ElasticNet | Unseen cultivars | 6 | 1659 | -0.09 | 0.591 | 865.087 | 195.75 | 667.016 | 143.58 | 0.117 | 0.028 | 0.351 | 0.186 | 0.441 | 0.178 |
| Forward-year prediction | F2 | LightGBM | Seen cultivars | 6 | 1001 | -0.174 | 0.469 | 882.139 | 189.095 | 694.221 | 144.829 | 0.116 | 0.023 | 0.293 | 0.2 | 0.434 | 0.202 |
| Forward-year prediction | F2 | LightGBM | Unseen cultivars | 6 | 1659 | -0.131 | 0.426 | 897.524 | 158.071 | 710.737 | 121.11 | 0.121 | 0.021 | 0.326 | 0.147 | 0.423 | 0.146 |
| Forward-year prediction | F2 | LinearRegression | Seen cultivars | 6 | 1001 | -0.301 | 0.589 | 915.592 | 181.063 | 717.719 | 164.344 | 0.12 | 0.024 | 0.405 | 0.167 | 0.405 | 0.211 |
| Forward-year prediction | F2 | LinearRegression | Unseen cultivars | 6 | 1659 | -0.358 | 0.618 | 973.747 | 188.151 | 756.847 | 144.084 | 0.131 | 0.027 | 0.372 | 0.18 | 0.397 | 0.184 |
| Forward-year prediction | F2 | Random forest | Seen cultivars | 6 | 1001 | 0.053 | 0.256 | 795.972 | 57.816 | 620.446 | 60.137 | 0.105 | 0.008 | 0.367 | 0.187 | 0.493 | 0.234 |
| Forward-year prediction | F2 | Random forest | Unseen cultivars | 6 | 1659 | 0.075 | 0.266 | 819.457 | 111.126 | 640.717 | 60.819 | 0.11 | 0.016 | 0.366 | 0.122 | 0.465 | 0.143 |
| Forward-year prediction | F2 | Ridge | Seen cultivars | 6 | 1001 | 0.012 | 0.555 | 783.566 | 157.466 | 609.019 | 122.785 | 0.103 | 0.023 | 0.398 | 0.164 | 0.507 | 0.235 |
| Forward-year prediction | F2 | Ridge | Unseen cultivars | 6 | 1659 | -0.1 | 0.596 | 868.829 | 197.332 | 670.837 | 142.924 | 0.117 | 0.028 | 0.343 | 0.18 | 0.441 | 0.178 |
| Forward-year prediction | F2 | XGBoost | Seen cultivars | 6 | 1001 | -0.02 | 0.303 | 825.017 | 105.642 | 643.183 | 86.459 | 0.108 | 0.014 | 0.341 | 0.192 | 0.447 | 0.209 |
| Forward-year prediction | F2 | XGBoost | Unseen cultivars | 6 | 1659 | -0.036 | 0.331 | 862.448 | 126.826 | 676.417 | 78.091 | 0.116 | 0.018 | 0.307 | 0.175 | 0.399 | 0.125 |
| Forward-year prediction | F3 | CatBoost | Seen cultivars | 6 | 1001 | 0.037 | 0.307 | 797.902 | 85.929 | 615.953 | 66.421 | 0.105 | 0.011 | 0.386 | 0.217 | 0.508 | 0.184 |
| Forward-year prediction | F3 | CatBoost | Unseen cultivars | 6 | 1659 | 0.061 | 0.254 | 826.562 | 103.797 | 650.102 | 65.259 | 0.111 | 0.015 | 0.302 | 0.144 | 0.443 | 0.133 |
| Forward-year prediction | F3 | ElasticNet | Seen cultivars | 6 | 1001 | 0.057 | 0.503 | 768.139 | 143.345 | 581.703 | 113.364 | 0.101 | 0.021 | 0.431 | 0.171 | 0.516 | 0.218 |
| Forward-year prediction | F3 | ElasticNet | Unseen cultivars | 6 | 1659 | -0.113 | 0.511 | 883.576 | 167.643 | 687.485 | 118.379 | 0.119 | 0.024 | 0.312 | 0.198 | 0.436 | 0.174 |
| Forward-year prediction | F3 | LightGBM | Seen cultivars | 6 | 1001 | -0.124 | 0.408 | 857.873 | 137.117 | 664.762 | 99.931 | 0.113 | 0.018 | 0.3 | 0.245 | 0.438 | 0.19 |
| Forward-year prediction | F3 | LightGBM | Unseen cultivars | 6 | 1659 | -0.011 | 0.262 | 856.143 | 100.178 | 682.08 | 77.892 | 0.115 | 0.014 | 0.306 | 0.18 | 0.431 | 0.145 |
| Forward-year prediction | F3 | LinearRegression | Seen cultivars | 6 | 1001 | -0.363 | 0.589 | 943.515 | 202.542 | 729.432 | 158.767 | 0.124 | 0.025 | 0.375 | 0.168 | 0.412 | 0.222 |
| Forward-year prediction | F3 | LinearRegression | Unseen cultivars | 6 | 1659 | -0.448 | 0.594 | 1010.853 | 188.462 | 787.27 | 139.45 | 0.136 | 0.026 | 0.311 | 0.173 | 0.397 | 0.167 |
| Forward-year prediction | F3 | Random forest | Seen cultivars | 6 | 1001 | 0.055 | 0.276 | 792.709 | 57.151 | 619.623 | 50.463 | 0.104 | 0.009 | 0.395 | 0.184 | 0.516 | 0.189 |
| Forward-year prediction | F3 | Random forest | Unseen cultivars | 6 | 1659 | 0.081 | 0.261 | 816.523 | 106.831 | 640.896 | 58.389 | 0.11 | 0.016 | 0.347 | 0.111 | 0.456 | 0.147 |
| Forward-year prediction | F3 | Ridge | Seen cultivars | 6 | 1001 | 0.035 | 0.501 | 778.953 | 137.395 | 591.419 | 105.065 | 0.103 | 0.02 | 0.419 | 0.16 | 0.512 | 0.217 |
| Forward-year prediction | F3 | Ridge | Unseen cultivars | 6 | 1659 | -0.127 | 0.511 | 889.687 | 166.729 | 693.411 | 113.957 | 0.12 | 0.024 | 0.303 | 0.192 | 0.444 | 0.184 |
| Forward-year prediction | F3 | XGBoost | Seen cultivars | 6 | 1001 | -0.036 | 0.343 | 826.261 | 95.399 | 641.706 | 57.134 | 0.109 | 0.013 | 0.325 | 0.222 | 0.413 | 0.21 |
| Forward-year prediction | F3 | XGBoost | Unseen cultivars | 6 | 1659 | 0.007 | 0.258 | 848.096 | 93.12 | 671.416 | 60.014 | 0.114 | 0.014 | 0.276 | 0.175 | 0.416 | 0.095 |
| Forward-year prediction | F4 | CatBoost | Seen cultivars | 6 | 1001 | 0.281 | 0.261 | 689.615 | 100.83 | 538.489 | 76.305 | 0.091 | 0.014 | 0.563 | 0.15 | 0.539 | 0.209 |
| Forward-year prediction | F4 | CatBoost | Unseen cultivars | 6 | 1659 | 0.317 | 0.204 | 703.215 | 95.204 | 552.784 | 52.361 | 0.095 | 0.014 | 0.558 | 0.142 | 0.513 | 0.112 |
| Forward-year prediction | F4 | ElasticNet | Seen cultivars | 6 | 1001 | 0.43 | 0.222 | 611.524 | 74.228 | 485.162 | 51.897 | 0.081 | 0.012 | 0.628 | 0.12 | 0.581 | 0.117 |
| Forward-year prediction | F4 | ElasticNet | Unseen cultivars | 6 | 1659 | 0.304 | 0.258 | 704.462 | 115.594 | 548.304 | 88.501 | 0.095 | 0.016 | 0.593 | 0.146 | 0.586 | 0.11 |
| Forward-year prediction | F4 | LightGBM | Seen cultivars | 6 | 1001 | 0.181 | 0.36 | 733.597 | 170.206 | 573.294 | 132.199 | 0.096 | 0.022 | 0.509 | 0.176 | 0.541 | 0.168 |
| Forward-year prediction | F4 | LightGBM | Unseen cultivars | 6 | 1659 | 0.22 | 0.295 | 745.225 | 119.999 | 590.299 | 102.025 | 0.1 | 0.016 | 0.539 | 0.152 | 0.509 | 0.055 |
| Forward-year prediction | F4 | LinearRegression | Seen cultivars | 6 | 1001 | -0.027 | 0.652 | 818.508 | 276.5 | 644.756 | 188.19 | 0.107 | 0.034 | 0.509 | 0.176 | 0.435 | 0.189 |
| Forward-year prediction | F4 | LinearRegression | Unseen cultivars | 6 | 1659 | -0.076 | 0.616 | 861.579 | 225.773 | 685.807 | 197.493 | 0.116 | 0.029 | 0.521 | 0.149 | 0.45 | 0.154 |
| Forward-year prediction | F4 | Random forest | Seen cultivars | 6 | 1001 | 0.167 | 0.239 | 745.782 | 64.746 | 586.805 | 57.072 | 0.098 | 0.01 | 0.472 | 0.155 | 0.52 | 0.208 |
| Forward-year prediction | F4 | Random forest | Unseen cultivars | 6 | 1659 | 0.223 | 0.173 | 752.697 | 64.013 | 592.226 | 27.936 | 0.101 | 0.01 | 0.494 | 0.107 | 0.506 | 0.1 |
| Forward-year prediction | F4 | Ridge | Seen cultivars | 6 | 1001 | 0.416 | 0.22 | 619.34 | 69.398 | 493.676 | 46.258 | 0.082 | 0.012 | 0.615 | 0.118 | 0.56 | 0.127 |
| Forward-year prediction | F4 | Ridge | Unseen cultivars | 6 | 1659 | 0.295 | 0.257 | 709.453 | 111.207 | 552.43 | 86.268 | 0.095 | 0.015 | 0.585 | 0.142 | 0.562 | 0.105 |
| Forward-year prediction | F4 | XGBoost | Seen cultivars | 6 | 1001 | 0.286 | 0.248 | 688.235 | 97.908 | 542.152 | 77.22 | 0.091 | 0.013 | 0.551 | 0.139 | 0.557 | 0.202 |
| Forward-year prediction | F4 | XGBoost | Unseen cultivars | 6 | 1659 | 0.311 | 0.183 | 706.2 | 74.097 | 557.893 | 52.573 | 0.095 | 0.011 | 0.552 | 0.141 | 0.523 | 0.11 |
| Forward-year prediction | F5 | CatBoost | Seen cultivars | 6 | 1001 | -0.132 | 0.401 | 861.328 | 82.491 | 663.928 | 60.381 | 0.113 | 0.012 | 0.351 | 0.203 | 0.466 | 0.106 |
| Forward-year prediction | F5 | CatBoost | Unseen cultivars | 6 | 1659 | -0.054 | 0.28 | 875.409 | 105.092 | 682.892 | 59.645 | 0.118 | 0.016 | 0.273 | 0.157 | 0.456 | 0.134 |
| Forward-year prediction | F5 | ElasticNet | Seen cultivars | 6 | 1001 | 0.062 | 0.428 | 773.248 | 119.095 | 587.726 | 76.294 | 0.102 | 0.017 | 0.442 | 0.217 | 0.525 | 0.207 |
| Forward-year prediction | F5 | ElasticNet | Unseen cultivars | 6 | 1659 | -0.008 | 0.372 | 850.872 | 142.987 | 663.064 | 94.007 | 0.115 | 0.021 | 0.326 | 0.218 | 0.446 | 0.182 |
| Forward-year prediction | F5 | LightGBM | Seen cultivars | 6 | 1001 | -0.188 | 0.583 | 865.805 | 146.336 | 673.349 | 91.372 | 0.114 | 0.021 | 0.304 | 0.272 | 0.428 | 0.211 |
| Forward-year prediction | F5 | LightGBM | Unseen cultivars | 6 | 1659 | -0.101 | 0.362 | 889.62 | 134.143 | 693.393 | 73.624 | 0.12 | 0.02 | 0.277 | 0.19 | 0.392 | 0.166 |
| Forward-year prediction | F5 | LinearRegression | Seen cultivars | 6 | 1001 | -0.4 | 0.676 | 950.668 | 245.192 | 710.62 | 152.547 | 0.125 | 0.031 | 0.333 | 0.206 | 0.423 | 0.24 |
| Forward-year prediction | F5 | LinearRegression | Unseen cultivars | 6 | 1659 | -0.347 | 0.562 | 975.391 | 192.952 | 749.978 | 129.969 | 0.131 | 0.026 | 0.285 | 0.184 | 0.422 | 0.162 |
| Forward-year prediction | F5 | Random forest | Seen cultivars | 6 | 1001 | -0.077 | 0.397 | 839.236 | 84.441 | 655.24 | 69.177 | 0.11 | 0.012 | 0.345 | 0.252 | 0.474 | 0.177 |
| Forward-year prediction | F5 | Random forest | Unseen cultivars | 6 | 1659 | -0.013 | 0.289 | 858.104 | 117.122 | 671.975 | 72.245 | 0.116 | 0.017 | 0.29 | 0.181 | 0.423 | 0.135 |
| Forward-year prediction | F5 | Ridge | Seen cultivars | 6 | 1001 | 0.046 | 0.441 | 778.742 | 123.06 | 593.61 | 77.34 | 0.103 | 0.018 | 0.439 | 0.221 | 0.532 | 0.21 |
| Forward-year prediction | F5 | Ridge | Unseen cultivars | 6 | 1659 | -0.016 | 0.383 | 853.686 | 147.061 | 666.455 | 94.649 | 0.115 | 0.022 | 0.32 | 0.215 | 0.446 | 0.182 |
| Forward-year prediction | F5 | XGBoost | Seen cultivars | 6 | 1001 | -0.222 | 0.611 | 877.625 | 154.777 | 675.379 | 112.91 | 0.116 | 0.022 | 0.309 | 0.262 | 0.456 | 0.135 |
| Forward-year prediction | F5 | XGBoost | Unseen cultivars | 6 | 1659 | -0.107 | 0.377 | 889.591 | 133.149 | 689.538 | 71.26 | 0.12 | 0.02 | 0.26 | 0.185 | 0.415 | 0.137 |
| Leave-one-site-out | F0 | Environment mean | Seen cultivars | 10 | 3204 | -0.245 | 0.315 | 841.013 | 284.018 | 683.639 | 253.088 | 0.113 | 0.036 |  |  | 0.189 | 0.123 |
| Leave-one-site-out | F0 | Environment random-effect LMM | Seen cultivars | 10 | 3204 | -0.236 | 0.316 | 837.268 | 281.358 | 681.342 | 251.204 | 0.112 | 0.035 |  |  | 0.189 | 0.123 |
| Leave-one-site-out | F0 | Overall mean | Seen cultivars | 10 | 3204 | -0.245 | 0.315 | 841.013 | 284.018 | 683.639 | 253.088 | 0.113 | 0.036 |  |  | 0.189 | 0.123 |
| Leave-one-site-out | F1 | CatBoost | Seen cultivars | 10 | 3204 | -0.189 | 0.271 | 813.49 | 240.355 | 672.816 | 218.625 | 0.109 | 0.029 | 0.236 | 0.189 | 0.258 | 0.143 |
| Leave-one-site-out | F1 | ElasticNet | Seen cultivars | 10 | 3204 | -0.03 | 0.13 | 753.898 | 175.592 | 606.42 | 146.974 | 0.101 | 0.022 | 0.297 | 0.145 | 0.299 | 0.142 |
| Leave-one-site-out | F1 | LightGBM | Seen cultivars | 10 | 3204 | -0.331 | 0.29 | 857.117 | 234.203 | 704.066 | 221.831 | 0.114 | 0.027 | 0.179 | 0.101 | 0.276 | 0.132 |
| Leave-one-site-out | F1 | LinearRegression | Seen cultivars | 10 | 3204 | -0.078 | 0.151 | 769.608 | 176.032 | 618.546 | 145.633 | 0.103 | 0.021 | 0.262 | 0.164 | 0.303 | 0.141 |
| Leave-one-site-out | F1 | Random forest | Seen cultivars | 10 | 3204 | -0.154 | 0.266 | 800.996 | 234.784 | 657.55 | 211.64 | 0.107 | 0.028 | 0.227 | 0.152 | 0.299 | 0.123 |
| Leave-one-site-out | F1 | Ridge | Seen cultivars | 10 | 3204 | -0.04 | 0.129 | 756.565 | 171.986 | 607.927 | 142.676 | 0.101 | 0.021 | 0.289 | 0.138 | 0.3 | 0.143 |
| Leave-one-site-out | F1 | XGBoost | Seen cultivars | 10 | 3204 | -0.236 | 0.231 | 828.289 | 223.959 | 681.354 | 203.575 | 0.111 | 0.028 | 0.183 | 0.17 | 0.311 | 0.176 |
| Leave-one-site-out | F2 | CatBoost | Seen cultivars | 10 | 3204 | -0.179 | 0.321 | 803.26 | 229.552 | 659.11 | 214.273 | 0.107 | 0.027 | 0.285 | 0.179 | 0.345 | 0.152 |
| Leave-one-site-out | F2 | ElasticNet | Seen cultivars | 10 | 3204 | -0.075 | 0.125 | 772.97 | 189.597 | 621.638 | 154.464 | 0.104 | 0.024 | 0.278 | 0.173 | 0.265 | 0.112 |
| Leave-one-site-out | F2 | LightGBM | Seen cultivars | 10 | 3204 | -0.211 | 0.267 | 815.486 | 215.925 | 667.835 | 200.082 | 0.109 | 0.026 | 0.25 | 0.17 | 0.312 | 0.135 |
| Leave-one-site-out | F2 | LinearRegression | Seen cultivars | 10 | 3204 | -0.146 | 0.099 | 800.119 | 197.245 | 640.414 | 154.642 | 0.107 | 0.026 | 0.23 | 0.212 | 0.239 | 0.143 |
| Leave-one-site-out | F2 | Random forest | Seen cultivars | 10 | 3204 | -0.119 | 0.259 | 789.188 | 234.932 | 646.627 | 212.911 | 0.105 | 0.028 | 0.266 | 0.173 | 0.315 | 0.123 |
| Leave-one-site-out | F2 | Ridge | Seen cultivars | 10 | 3204 | -0.093 | 0.122 | 779.262 | 187.359 | 625.295 | 150.515 | 0.104 | 0.024 | 0.268 | 0.177 | 0.266 | 0.111 |
| Leave-one-site-out | F2 | XGBoost | Seen cultivars | 10 | 3204 | -0.236 | 0.297 | 829.262 | 251.976 | 683.704 | 233.014 | 0.111 | 0.03 | 0.253 | 0.158 | 0.314 | 0.117 |
| Leave-one-site-out | F3 | CatBoost | Seen cultivars | 10 | 3204 | -0.093 | 0.293 | 778.829 | 241.257 | 639.848 | 221.764 | 0.104 | 0.029 | 0.384 | 0.142 | 0.334 | 0.11 |
| Leave-one-site-out | F3 | ElasticNet | Seen cultivars | 10 | 3204 | -0.017 | 0.141 | 753.715 | 199.443 | 610.501 | 167.442 | 0.101 | 0.025 | 0.39 | 0.157 | 0.326 | 0.126 |
| Leave-one-site-out | F3 | LightGBM | Seen cultivars | 10 | 3204 | -0.156 | 0.315 | 797.603 | 235.253 | 654.164 | 215.805 | 0.107 | 0.029 | 0.346 | 0.155 | 0.334 | 0.134 |
| Leave-one-site-out | F3 | LinearRegression | Seen cultivars | 10 | 3204 | -0.083 | 0.093 | 779.117 | 199.792 | 628.109 | 162.438 | 0.104 | 0.026 | 0.352 | 0.181 | 0.313 | 0.134 |
| Leave-one-site-out | F3 | Random forest | Seen cultivars | 10 | 3204 | -0.063 | 0.271 | 771.759 | 246.519 | 632.516 | 223.309 | 0.103 | 0.03 | 0.361 | 0.16 | 0.32 | 0.087 |
| Leave-one-site-out | F3 | Ridge | Seen cultivars | 10 | 3204 | -0.036 | 0.13 | 760.503 | 197.259 | 615.075 | 163.864 | 0.102 | 0.025 | 0.378 | 0.159 | 0.313 | 0.129 |
| Leave-one-site-out | F3 | XGBoost | Seen cultivars | 10 | 3204 | -0.15 | 0.292 | 796.679 | 234.813 | 655.772 | 216.289 | 0.106 | 0.028 | 0.379 | 0.148 | 0.354 | 0.111 |
| Leave-one-site-out | F4 | CatBoost | Seen cultivars | 10 | 3204 | 0.265 | 0.227 | 634.215 | 180.671 | 521.188 | 155.692 | 0.085 | 0.022 | 0.615 | 0.099 | 0.469 | 0.092 |
| Leave-one-site-out | F4 | ElasticNet | Seen cultivars | 10 | 3204 | 0.29 | 0.228 | 618.026 | 150.081 | 493.874 | 117.849 | 0.083 | 0.019 | 0.648 | 0.076 | 0.506 | 0.13 |
| Leave-one-site-out | F4 | LightGBM | Seen cultivars | 10 | 3204 | 0.25 | 0.2 | 641.792 | 168.919 | 524.456 | 143.547 | 0.086 | 0.022 | 0.61 | 0.082 | 0.474 | 0.117 |
| Leave-one-site-out | F4 | LinearRegression | Seen cultivars | 10 | 3204 | 0.236 | 0.226 | 642.458 | 155.769 | 515.963 | 120.883 | 0.086 | 0.02 | 0.619 | 0.084 | 0.484 | 0.138 |
| Leave-one-site-out | F4 | Random forest | Seen cultivars | 10 | 3204 | 0.12 | 0.264 | 702.036 | 233.33 | 578.346 | 210.162 | 0.094 | 0.028 | 0.504 | 0.117 | 0.405 | 0.118 |
| Leave-one-site-out | F4 | Ridge | Seen cultivars | 10 | 3204 | 0.278 | 0.233 | 623.053 | 151.264 | 498.777 | 119.032 | 0.083 | 0.019 | 0.642 | 0.078 | 0.497 | 0.128 |
| Leave-one-site-out | F4 | XGBoost | Seen cultivars | 10 | 3204 | 0.257 | 0.201 | 640.621 | 181.284 | 525.572 | 156.974 | 0.086 | 0.023 | 0.614 | 0.08 | 0.495 | 0.108 |
| Leave-one-site-out | F5 | CatBoost | Seen cultivars | 10 | 3204 | -0.114 | 0.295 | 789.546 | 254.75 | 643.111 | 227.881 | 0.105 | 0.031 | 0.371 | 0.151 | 0.343 | 0.101 |
| Leave-one-site-out | F5 | ElasticNet | Seen cultivars | 10 | 3204 | -0.033 | 0.213 | 765.295 | 246.257 | 618.03 | 210.545 | 0.102 | 0.032 | 0.407 | 0.162 | 0.362 | 0.138 |
| Leave-one-site-out | F5 | LightGBM | Seen cultivars | 10 | 3204 | -0.219 | 0.347 | 827.561 | 276.68 | 679.429 | 254.409 | 0.111 | 0.034 | 0.334 | 0.18 | 0.33 | 0.109 |
| Leave-one-site-out | F5 | LinearRegression | Seen cultivars | 10 | 3204 | -0.154 | 0.312 | 812.797 | 295.738 | 659.996 | 258.285 | 0.109 | 0.038 | 0.37 | 0.179 | 0.325 | 0.12 |
| Leave-one-site-out | F5 | Random forest | Seen cultivars | 10 | 3204 | -0.121 | 0.31 | 793.196 | 262.152 | 648.005 | 235.263 | 0.106 | 0.032 | 0.352 | 0.167 | 0.347 | 0.118 |
| Leave-one-site-out | F5 | Ridge | Seen cultivars | 10 | 3204 | -0.047 | 0.211 | 770.87 | 247.771 | 622.111 | 211.083 | 0.103 | 0.032 | 0.401 | 0.166 | 0.344 | 0.138 |
| Leave-one-site-out | F5 | XGBoost | Seen cultivars | 10 | 3204 | -0.184 | 0.344 | 817.618 | 284.023 | 666.392 | 256.937 | 0.109 | 0.035 | 0.335 | 0.164 | 0.338 | 0.083 |

**Supplementary Table 18.** Extreme-environment sensitivity tests

| Risk definition | Metric | Flagged environments | Unflagged environments | Flagged mean | Unflagged mean | Difference flagged minus unflagged | Welch t p | Mann whitney p |
| --- | --- | --- | --- | --- | --- | --- | --- | --- |
| At least one risk | r2 | 24 | 43 | -0.046 | -0.072 | 0.026 | 0.921 | 0.891 |
| At least one risk | rmse | 24 | 43 | 563.588 | 542.441 | 21.147 | 0.709 | 0.68 |
| At least one risk | nrmse | 24 | 43 | 0.076 | 0.073 | 0.003 | 0.7 | 0.911 |
| At least one risk | top20 recall | 24 | 43 | 0.611 | 0.441 | 0.17 | 0.001 | 0.002 |
| Two or more risks | r2 | 7 | 60 | -0.116 | -0.056 | -0.06 | 0.855 | 0.681 |
| Two or more risks | rmse | 7 | 60 | 478.211 | 558.393 | -80.182 | 0.309 | 0.392 |
| Two or more risks | nrmse | 7 | 60 | 0.063 | 0.075 | -0.012 | 0.24 | 0.359 |
| Two or more risks | top20 recall | 7 | 60 | 0.566 | 0.495 | 0.071 | 0.333 | 0.35 |
| high temperature | r2 | 7 | 60 | -0.108 | -0.057 | -0.051 | 0.862 | 0.438 |
| high temperature | rmse | 7 | 60 | 638.602 | 539.681 | 98.921 | 0.466 | 0.415 |
| high temperature | nrmse | 7 | 60 | 0.083 | 0.073 | 0.01 | 0.585 | 0.741 |
| high temperature | top20 recall | 7 | 60 | 0.591 | 0.492 | 0.099 | 0.179 | 0.267 |
| heavy rainfall | r2 | 7 | 60 | -0.103 | -0.058 | -0.045 | 0.882 | 0.488 |
| heavy rainfall | rmse | 7 | 60 | 521.094 | 553.39 | -32.296 | 0.692 | 0.96 |
| heavy rainfall | nrmse | 7 | 60 | 0.07 | 0.074 | -0.005 | 0.68 | 0.912 |
| heavy rainfall | top20 recall | 7 | 60 | 0.722 | 0.477 | 0.246 | 0.004 | 0.003 |
| high humidity | r2 | 7 | 60 | -0.285 | -0.037 | -0.248 | 0.712 | 0.527 |
| high humidity | rmse | 7 | 60 | 490.097 | 557.007 | -66.91 | 0.515 | 0.279 |
| high humidity | nrmse | 7 | 60 | 0.07 | 0.074 | -0.005 | 0.764 | 0.392 |
| high humidity | top20 recall | 7 | 60 | 0.537 | 0.498 | 0.039 | 0.669 | 0.91 |
| low radiation | r2 | 7 | 60 | -0.045 | -0.065 | 0.02 | 0.942 | 0.594 |
| low radiation | rmse | 7 | 60 | 512.326 | 554.413 | -42.087 | 0.53 | 0.681 |
| low radiation | nrmse | 7 | 60 | 0.068 | 0.075 | -0.006 | 0.486 | 0.696 |
| low radiation | top20 recall | 7 | 60 | 0.534 | 0.498 | 0.036 | 0.559 | 0.593 |
| low sunshine | r2 | 7 | 60 | 0.196 | -0.093 | 0.288 | 0.241 | 0.553 |
| low sunshine | rmse | 7 | 60 | 469.897 | 559.363 | -89.467 | 0.143 | 0.438 |
| low sunshine | nrmse | 7 | 60 | 0.062 | 0.075 | -0.013 | 0.122 | 0.475 |
| low sunshine | top20 recall | 7 | 60 | 0.591 | 0.492 | 0.099 | 0.249 | 0.246 |

**Supplementary Table 19.** Additional baseline model comparisons

| Validation scheme | Model | Splits | Records total | Mean R2 | SD of R2 | Mean RMSE | SD of RMSE | Mean MAE | SD of MAE | Mean normalized RMSE | SD of normalized RMSE | Mean Spearman correlation | SD of Spearman correlation | Mean top-20% recall | SD of top-20% recall |
| --- | --- | --- | --- | --- | --- | --- | --- | --- | --- | --- | --- | --- | --- | --- | --- |
| Forward-year prediction | Simple climate linear regression | 6 | 2660 | -0.017 | 0.405 | 845.134 | 138.349 | 650.608 | 95.945 | 0.113 | 0.02 | 0.301 | 0.135 | 0.49 | 0.176 |
| Forward-year prediction | Cultivar BLUP-only | 6 | 2660 | -0.04 | 0.105 | 871.591 | 19.714 | 677.754 | 35.029 | 0.116 | 0.002 | 0.209 | 0.134 | 0.29 | 0.079 |
| Leave-one-site-out | Cultivar BLUP-only | 10 | 3204 | -0.09 | 0.299 | 787.054 | 273.724 | 638.42 | 238.444 | 0.105 | 0.035 | 0.413 | 0.113 | 0.366 | 0.07 |
| Leave-one-site-out | Simple climate linear regression | 10 | 3204 | -0.095 | 0.181 | 785.28 | 229.749 | 634.584 | 195.845 | 0.105 | 0.03 | 0.268 | 0.145 | 0.29 | 0.131 |
| Leave-one-site-year-out | Simple climate linear regression | 67 | 3204 | -0.707 | 1.359 | 728.628 | 294.899 | 606.713 | 259.694 | 0.098 | 0.041 | 0.221 | 0.401 | 0.238 | 0.178 |
| Leave-one-site-year-out | Cultivar BLUP-only | 67 | 3204 | -0.879 | 1.845 | 732.266 | 307.798 | 614.303 | 288.339 | 0.098 | 0.041 | 0.45 | 0.302 | 0.372 | 0.2 |
| Leave-one-year-out | Simple climate linear regression | 7 | 3204 | 0.124 | 0.378 | 776.529 | 156.887 | 607.285 | 112.267 | 0.104 | 0.022 | 0.39 | 0.191 | 0.505 | 0.161 |
| Leave-one-year-out | Cultivar BLUP-only | 7 | 3204 | -0.018 | 0.075 | 854.691 | 55.572 | 663.491 | 61.453 | 0.114 | 0.007 | 0.223 | 0.122 | 0.288 | 0.066 |
| Random five-fold CV | Simple climate linear regression | 5 | 3204 | 0.331 | 0.029 | 705.834 | 9.477 | 545.93 | 13.197 | 0.094 | 0.002 | 0.51 | 0.009 | 0.53 | 0.036 |
| Random five-fold CV | Cultivar BLUP-only | 5 | 3204 | 0.147 | 0.05 | 796.676 | 12.304 | 614.307 | 7.897 | 0.106 | 0.002 | 0.393 | 0.077 | 0.342 | 0.035 |

**Supplementary Table 20.** Micro-averaged additional baseline model metrics

| Validation scheme | Model | Aggregation | Records | R2 | Rmse | Mae | Nrmse | Spearman | Top20 recall |
| --- | --- | --- | --- | --- | --- | --- | --- | --- | --- |
| Forward-year prediction | Simple climate linear regression | micro | 2660 | 0.071 | 847.126 | 646.761 | 0.113 | 0.325 | 0.489 |
| Forward-year prediction | Cultivar BLUP-only | micro | 2660 | 0.014 | 872.502 | 679.995 | 0.116 | 0.153 | 0.297 |
| Leave-one-site-out | Simple climate linear regression | micro | 3204 | 0.112 | 814.515 | 633.608 | 0.109 | 0.333 | 0.353 |
| Leave-one-site-out | Cultivar BLUP-only | micro | 3204 | 0.065 | 836.115 | 642.876 | 0.112 | 0.289 | 0.24 |
| Leave-one-site-year-out | Simple climate linear regression | micro | 3204 | 0.168 | 788.431 | 606.24 | 0.105 | 0.392 | 0.524 |
| Leave-one-site-year-out | Cultivar BLUP-only | micro | 3204 | 0.111 | 815.297 | 627.454 | 0.109 | 0.341 | 0.306 |
| Leave-one-year-out | Simple climate linear regression | micro | 3204 | 0.191 | 777.509 | 598.455 | 0.104 | 0.408 | 0.524 |
| Leave-one-year-out | Cultivar BLUP-only | micro | 3204 | 0.021 | 855.54 | 663.463 | 0.114 | 0.16 | 0.265 |
| Random five-fold CV | Simple climate linear regression | micro | 3204 | 0.333 | 705.885 | 545.928 | 0.094 | 0.511 | 0.526 |
| Random five-fold CV | Cultivar BLUP-only | micro | 3204 | 0.151 | 796.752 | 614.308 | 0.106 | 0.393 | 0.343 |

**Supplementary Table 21.** Nested tuning sensitivity check

| Validation scheme | Model | Mode | Splits | Records total | Mean R2 | SD of R2 | Mean RMSE | SD of RMSE | Mean MAE | SD of MAE | Mean normalized RMSE | SD of normalized RMSE | Mean Spearman correlation | SD of Spearman correlation | Mean top-20% recall | SD of top-20% recall |
| --- | --- | --- | --- | --- | --- | --- | --- | --- | --- | --- | --- | --- | --- | --- | --- | --- |
| Forward-year prediction | ElasticNet | Fixed default | 6 | 2660 | 0.358 | 0.229 | 674.353 | 92.205 | 525.963 | 66.578 | 0.09 | 0.013 | 0.607 | 0.136 | 0.58 | 0.13 |
| Forward-year prediction | LightGBM | Fixed default | 6 | 2660 | 0.224 | 0.335 | 736.913 | 144.86 | 580.675 | 113.547 | 0.098 | 0.019 | 0.543 | 0.162 | 0.518 | 0.114 |
| Forward-year prediction | ElasticNet | Inner-CV tuned | 6 | 2660 | 0.281 | 0.277 | 711.602 | 88.08 | 562.887 | 71.208 | 0.095 | 0.013 | 0.576 | 0.14 | 0.53 | 0.195 |
| Forward-year prediction | LightGBM | Inner-CV tuned | 6 | 2660 | 0.234 | 0.271 | 736.528 | 108.717 | 575.58 | 79.623 | 0.098 | 0.015 | 0.522 | 0.145 | 0.499 | 0.131 |
| Leave-one-site-out | ElasticNet | Fixed default | 10 | 3204 | 0.289 | 0.228 | 618.552 | 150.856 | 494.297 | 118.384 | 0.083 | 0.019 | 0.647 | 0.076 | 0.507 | 0.132 |
| Leave-one-site-out | LightGBM | Fixed default | 10 | 3204 | 0.235 | 0.195 | 648.664 | 169.015 | 533.467 | 145.509 | 0.087 | 0.022 | 0.603 | 0.074 | 0.469 | 0.099 |
| Leave-one-site-out | ElasticNet | Inner-CV tuned | 10 | 3204 | 0.251 | 0.228 | 636.048 | 155.101 | 509.533 | 121.285 | 0.085 | 0.02 | 0.628 | 0.083 | 0.485 | 0.135 |
| Leave-one-site-out | LightGBM | Inner-CV tuned | 10 | 3204 | 0.238 | 0.176 | 647.768 | 165.062 | 532.651 | 142.078 | 0.087 | 0.022 | 0.605 | 0.071 | 0.46 | 0.125 |

**Supplementary Table 22.** Cross-algorithm SHAP ranking stability

| Feature set | Comparison | Common features | Spearman rank correlation | Top5 overlap count | Top5 overlap features | Top10 overlap count | Top10 overlap features |
| --- | --- | --- | --- | --- | --- | --- | --- |
| F3 | LightGBM vs RandomForest | 24 | 0.684 | 3 | TestSite; Yield BLUP value; tp sum sow heading | 8 | RH mean sow heading; Sowing DOY; TestSite; Yield BLUP value; ssrd sum heading maturity; tmin mean heading maturity; tmin mean sow heading; tp sum sow heading |
| F4 | LightGBM vs RandomForest | 34 | 0.728 | 3 | FGPan; SSR; Yield BLUP value | 8 | EP; FGPan; PH; SSR; Sowing DOY; TGPan; TestSite; Yield BLUP value |
| F5 | LightGBM vs RandomForest | 15 | 0.918 | 4 | Sowing DOY; TestSite; Yield BLUP value; tmin mean sow heading | 8 | RH mean sow heading; Sowing DOY; TestSite; Yield BLUP value; t2m mean sow heading; tmax mean sow heading; tmin mean sow heading; tp sum sow heading |

**Supplementary Table 23.** Climate-feature correlation clusters

| Cluster id | Features | Feature count | Max within cluster abs spearman |
| --- | --- | --- | --- |
| 12 | t2m mean sow heading; t2m mean sow maturity; tmax mean sow heading; tmax mean sow maturity; tmin mean sow heading; tmin mean sow maturity | 6 | 0.981 |
| 11 | t2m mean heading maturity; tmax mean heading maturity; tmin mean heading maturity | 3 | 0.945 |
| 3 | active GDD sum heading maturity; effective GDD sum heading maturity | 2 | 0.893 |
| 6 | RH mean sow heading; RH mean sow maturity | 2 | 0.874 |
| 7 | ssd sum sow heading; ssd sum sow maturity | 2 | 0.951 |
| 8 | ssrd sum sow heading; ssrd sum sow maturity | 2 | 0.904 |
| 9 | effective GDD sum sow heading; effective GDD sum sow maturity | 2 | 0.869 |
| 10 | active GDD sum sow heading; active GDD sum sow maturity | 2 | 0.852 |
| 1 | Total precipitation: sowing to heading | 1 | 1 |
| 2 | tp sum sow maturity | 1 | 1 |
| 4 | ssd sum heading maturity | 1 | 1 |
| 5 | ssrd sum heading maturity | 1 | 1 |
| 13 | RH mean heading maturity | 1 | 1 |
| 14 | tp sum heading maturity | 1 | 1 |
